# Synthesis and Assessment of the In Vitro and Ex Vivo Activity of Salicylate Synthase (Mbti) Inhibitors as New Candidates for the Treatment of Mycobacterial Infections

**DOI:** 10.3390/ph15080992

**Published:** 2022-08-11

**Authors:** Matteo Mori, Giovanni Stelitano, Anna Griego, Laurent R. Chiarelli, Giulia Cazzaniga, Arianna Gelain, Elena Pini, Marina Camera, Paola Canzano, Andrea Fumagalli, Edoardo Scarpa, Chiara Cordiglieri, Loris Rizzello, Stefania Villa, Fiorella Meneghetti

**Affiliations:** 1 Department of Pharmaceutical Sciences, University of Milan, Via L. Mangiagalli 25, 20133 Milano, Italy; 2 Department of Biology and Biotechnology “Lazzaro Spallanzani”, University of Pavia, Via A. Ferrata 9, 27100 Pavia, Italy; 3 National Institute of Molecular Genetic (INGM), Via F. Sforza 35, 20122 Milano, Italy; 4 Centro Cardiologico Monzino, Istituto di Ricerca e Cura a Carattere Scientifico, Via C. Parea 4, 20138 Milano, Italy

**Keywords:** tuberculosis, mycobactins, furan, siderophores, drug development, drug resistance, murine alveolar-like macrophages

## Abstract

Tuberculosis (TB) causes millions of deaths every year, ranking as one of the most dangerous infectious diseases worldwide. Because several pathogenic strains of *M. tuberculosis* (*Mtb*) have developed resistance against most of the established anti-TB drugs, new therapeutic options are urgently needed. An attractive target for the development of new anti-TB agents is the salicylate synthase MbtI, the first enzyme of the mycobacterial siderophore biochemical machinery, absent in human cells. In this work, a set of analogues of 5-(3-cyanophenyl)furan-2-carboxylic acid (**I**), the most potent MbtI inhibitor identified to date, was synthesized, characterized, and tested to further elucidate the structural requirements for achieving an efficient MbtI inhibition and potent antitubercular activity. The structure–activity relationships (SAR) discussed herein evidenced the importance of the side chain linked to the phenyl moiety to improve the in vitro antimycobacterial activity. In detail, **1f** emerged as the most effective analogue against the pathogen, acting without cytotoxicity issues. To deepen the understanding of its mechanism of action, we established a fluorescence-based screening test to quantify the pathogen infectivity within host cells, using MPI-2 murine cells, a robust surrogate for alveolar macrophages. The set-up of the new assay demonstrates significant potential to accelerate the discovery of new anti-TB drugs.

## 1. Introduction

Tuberculosis (TB), an infectious disease caused by *M. tuberculosis* (*Mtb*), is one of the top 10 causes of death worldwide and the second leading cause from a single infectious agent, after COVID-19. In 2020, TB caused 1.3 million deaths, and about 11 million new infections. Almost three million people diagnosed with bacteriologically confirmed pulmonary TB were tested for rifampicin resistance (RR-TB). Among these, ≈130,000 cases of multi-drug-resistant (MDR)-TB and ≈25,000 cases of extensively drug-resistant (XDR)-TB were detected [1,2]. The spread of resistant strains is sustained by the continued use of traditional anti-TB drugs, which target biomolecules involved in *Mtb* growth and replication, thus inducing mutations in the bacterial targets [3,4]. This phenomenon has significantly hampered the perspectives of TB control and elimination. Nevertheless, many efforts have been made by the scientific community, as demonstrated by the new anti-TB drug candidates that have reached clinical trials in the last ten years [5,6]. Unfortunately, TB is often not alone in threatening the life of the patients. Individuals suffering from primary *Mtb* infections and lung morbidities are predisposed to be also affected by non-tubercular mycobacteria (NTM), especially by the highly virulent *M. abscessus* (*Mab*), which establishes chronic and drug-recalcitrant conditions that are difficult to treat with the available antimicrobials [7,8].

For these reasons, there is an urgent need for the identification of compounds with different mechanisms of action that can be used in combination with the existing drugs to shorten the treatment and improve its efficacy [9]. A promising strategy is to target pathogen virulence factors to prevent the establishment of the infection and the survival of the mycobacteria in the host. This approach does not exert a selective pressure on the microorganism, thereby reducing the likelihood of resistance phenomena. Because these anti-virulence agents are designed to complement and, possibly, enhance the effect of established anti-TB drugs, they could perfectly fit into the current therapeutic regimens, which are based on the combination of antimicrobials [10,11]. Hence, efforts should also be dedicated to the development of novel pharmaceutical forms to simplify and improve the co-administration of drugs [12,13].

**Figure 1:**
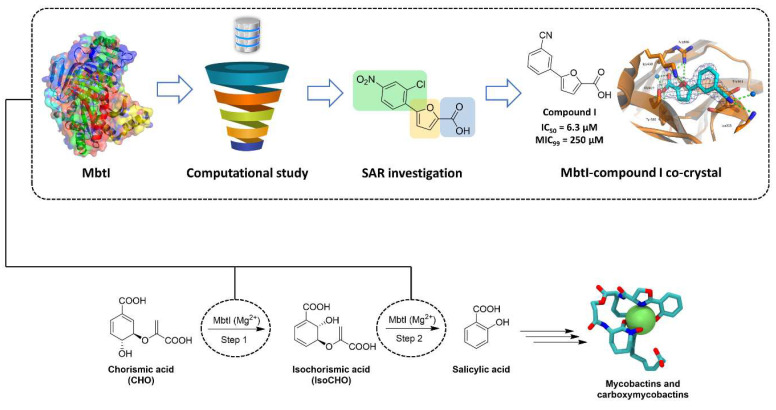
Schematic overview summarizing the contribution of our group to the discovery of new MbtI inhibitors.

In this context, iron acquisition offers a very promising avenue for therapeutic development against mycobacteria [14,15,16] because this metal acts as an essential cofactor to establish the infection and sustain the proliferation of the bacilli. Therefore, iron deprivation, which can be conveniently achieved by targeting enzymes involved in iron uptake, has been suggested as an attractive and alternative approach to treating mycobacterial infections [17]. Indeed, the introduction of mutations in these enzymes resulted in a reduction of the growth of the microorganisms in macrophages and a decreased virulence in mice [15]. Among the strategies evolved by mycobacteria to counteract host-mediated iron deprivation, the release of high-affinity siderophores (mycobactins and carboxymycobactins) represents the most efficient system. Notably, this biochemical machinery is significantly upregulated under iron-deficient conditions, suggesting its relevance in enhancing mycobacterial virulence and endurance during the infection process. Considering that siderophores are absent in host mammalian cells and that their depletion results in anti-tubercular effects, we can conclude that they represent a promising target for the development of innovative therapies against mycobacterial infections. In this context, our group has successfully identified the most potent inhibitors of the salicylate synthase (MbtI) from *Mtb*, the first enzyme of the siderophore biosynthetic pathway [9,18,19]. A summary of our efforts in the field is illustrated in Figure 1.

Although the promising effects of MbtI inhibitors on mycobacteria were previously demonstrated, the role of these compounds in a simulated physiological environment has never been investigated. Understanding the mechanisms of the pathogen–macrophage interaction is critical to shedding light on the pathways connecting both bacterial virulence and host immunity. For this purpose, the murine alveolar-like macrophage (mAMs) model can be conveniently used, considering that AMs, together with mucosal cells, are the first line of defense against pathogenic mycobacteria [20]. Hence, we decided to study the effects of MbtI inhibitors on Max Planck Institute cells (MPI-2) [21]. These cells were previously demonstrated to closely reproduce the natural niche of *Mtb* in vitro and may represent the future benchmark for the determination of anti-TB drug activity [22].

Starting from the recently reported crystal structure of MbtI complexed with 5-(3-cyanophenyl)furan-2-carboxylic acid (**I**) [9], which highlighted exploitable ligand features for the development of improved candidates, we focused our studies on the enhancement of its antimycobacterial potential through an increase in the cell wall permeability. To this end, we chose to explore the chemical space around the 5-position of the phenyl ring by adding a set of lipophilic moieties, considering both the crystallographic data and the observation that the *meta*-substitution with different groups was not detrimental for the activity [19].

In this work, we report the discovery of a new MbtI inhibitor (Table 1, compound **1f**), which displayed a very good potency against the target MbtI (IC_50_ = 12 μM), no cytotoxicity against human cells, and an improved in vitro activity on mycobacterial growth (MIC_99_ = 63 μM) in an iron-limiting medium. The Universal Chrome Azurol S (CAS) assay confirmed the connection between the antimycobacterial effect of **1f** and the disruption of mycobactin biosynthesis. In addition, we applied the newly developed model to assess the intracellular activity of **1f** in a setting that closely reproduces an in vivo-like scenario. The set-up of the assay, ex-novo infection and selection protocol performed on genetically engineered MPI-2 cells is also described.

## 2. Results

### 2.1. Chemistry

**Scheme 1:**
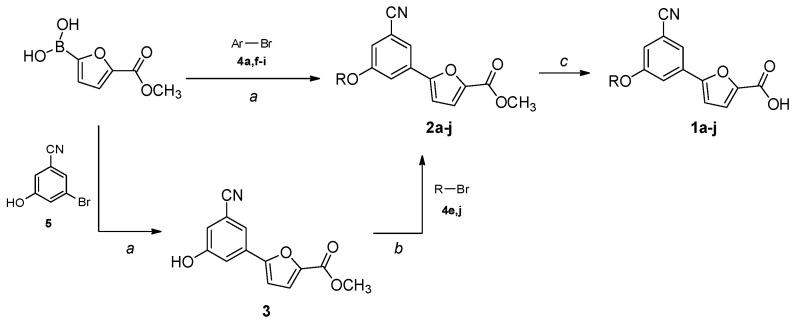
Synthetic procedure for the preparation of **1a**–**j**. Ar- and R-groups are listed in Table 1. **Reagents and conditions:** (***a***) Pd(PPh_3_)_2_Cl_2_, 2 M Na_2_CO_3_, 1,4-dioxane, 60 °C, 80 min, MW, N_2_ atm.; (***b***) K_2_CO_3_, acetone, reflux, 3–72 h, N_2_ atm.; (***c***) LiOH·H_2_O, THF/H_2_O 2:1, r.t., 1–6 h.

The compounds were synthesized by two similar approaches starting from (5-(methoxycarbonyl)furan-2-yl)boronic acid, obtained as previously described [18]. A Suzuki–Miyaura cross-coupling with a properly substituted 3-bromobenzonitrile afforded the key intermediates **2a,f–i**, which were hydrolyzed to the final carboxylic acids **1a,f–i** in basic conditions (Scheme 1). Alternatively, the boronic acid was reacted with 3-bromo-5-hydroxybenzonitrile in a Suzuki reaction, and then the hydroxyl group was substituted with different lipophilic moieties to give the ester intermediates **2b–e,j**. The desired acids **1b–e,j** were again obtained by a traditional basic hydrolysis. The bromo-derivatives used in the coupling reactions were either purchased or prepared by nucleophilic substitution of 3-bromo-5-fluorobenzonitrile, or by bromination of the appropriate starting material; more details are provided in the Supplementary Materials.

### 2.2. Biological Studies

#### 2.2.1. Enzymatic Assays, Antimycobacterial Activity and Siderophore Activity Assays

The in vitro activity of compounds **1a–j** against MbtI and *M. bovis BCG* were determined as previously reported and are listed in Table 1 [19]. Notably, the MIC_99_ of derivatives **1a–j** was determined against the nonpathogenic *M. bovis BCG* in iron-limiting conditions (chelated Sauton’s Medium) using the REMA method. Moreover, siderophore activity in the supernatants of the treated cell cultures was determined.

**Figure 2:**
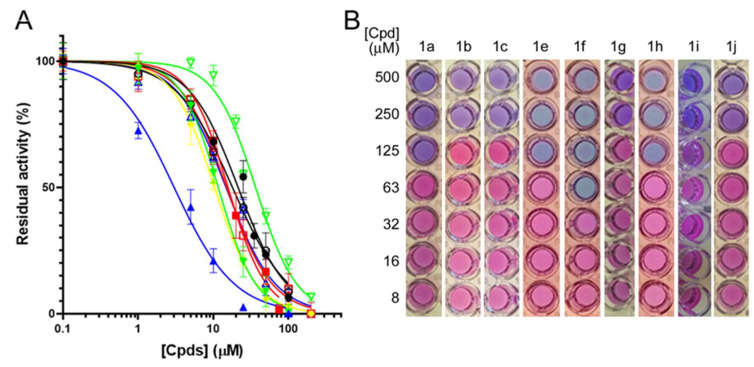
Biological characterization of **1a–j**. (**A**) IC_50_ determination against MbtI activity: ●, 1a; ▪, 1b; ▲, 1c; ▼, 1e; ♦ 1f; ◯, 1g; □, 1h; △, 1i; ▽, 1j. (**B**) MIC_99_ determination against *M. bovis BCG* growth.

With the aim of improving the permeability of the parent compound (**I**), we began by synthesizing **1a**, characterized by the presence of a phenoxy substituent; the inhibitor reduced the enzymatic activity (%RA, residual activity) to 14% at 100 μM, corresponding to an IC_50_ ≈ 23 μM (Figure 2A). Despite being less active than our lead compound (**I**), **1a** displayed an encouraging MIC_99_ (125 μM vs. 250 μM) against *M. bovis BCG* (Figure 2B).

To enhance the antimycobacterial potential of **1a**, we inserted an aliphatic linker with one, two, or three carbon atoms between the ethereal bridge and the phenyl moiety to move the aromatic ring away from the furan core. The inhibitory activity of **1b** increased with respect to **1a** (IC_50_ ≈ 15 μM vs. 23 µM). Most notably, while compound **1c**, carrying a phenethyl chain, proved to be very effective against MbtI with an IC_50_ of ≈ 3 μM (Figure 2A), **1d**, carrying a phenyl propyl chain, evidenced only a modest activity (≈25% RA at 100 μM). Unfortunately, these three analogs did not show better antimycobacterial activities with respect **1a**. Conversely, compound **1e**, functionalized with a cinnamic moiety, showed comparable inhibitory properties to **1b** (IC_50_ ≈ 13 μM vs. 15 µM) but a better antitubercular activity (MIC_99_ ≈ 125 μM). Therefore, starting from **1e**, we designed and synthesized two derivatives, in which the distal carbon atom of the aliphatic chain was replaced by an oxygen (**1f**) or a nitrogen (**1g**). The analogs **1f** and **1g** showed similar IC_50_ values (≈12 μM and ≈18 μM, respectively) but the former displayed the best antimycobacterial activity achieved with this class of compounds to date, with a MIC_99_ of 63 µM (Figure 1B). The Universal CAS assay confirmed that the antimycobacterial activity displayed by **1f** and the other derivatives was correlated to the inhibition of iron uptake (Figure S1). Further efforts to increase the lipophilic properties of the compounds using a naphthalene (**1h**), a quinoline (**1i**), or a methylnaphthalen moiety (**1j**) did not lead to an improvement in the antimycobacterial activity. Overall, **1f** emerged as the most promising candidate, characterized by halved IC_50_ and MIC_99_ values compared to **1a**, which highlighted its better druggability with respect to the previous analogs.

#### 2.2.2. Antimycobacterial Effect in AMs Infection Model

Encouraged by the enhanced antimycobacterial activity in vitro of **1f**, we explored its efficacy in preventing the progression of both tubercular (*M. bovis BCG*) and nontubercular (*Mab*) mycobacterial infections. To evaluate these parameters, we employed MPI-2 cells, which have been shown to be a better model for mycobacterial infections with respect to other cell lines [22]. To assess and visualize **1f**-mediated mycobacterial clearance, we preliminarily generated stable fluorescent *M. abscessus* (*Mab*) and *M. bovis BCG* reporter strains expressing tdTomato, respectively referred to as *Mab_tdTomato* and *BCG_tdTomato*. We also genetically modified MPI-2 cells with viral pseudoparticles to obtain mAMs stably expressing a fluorescent reporter, useful for the next segmentation-based imaging quantification of the infection. To generate this mAM reporter line, we transduced wild-type MPI-2 cells with viral pseudoparticles containing a transfer plasmid, inducing a post-integration eGFP constitutive expression (See Section 4). We confirmed the successful transduction, and the consequent expression of eGFP, by fluorescence-activated cell sorting (FACS), which was also used to sort and isolate the cells with the brightest expression of the fluorophore (Figure S2A). The collected population was further expanded in vitro and renamed as MPI-2-eGFP. Finally, we confirmed that the introduced genetic modification did not alter either MPI-2 cell morphology (Figure S2B) or the expression of specific immunogenic-related genes (Figure S2C).

Before testing the **1f**-mediated mycobacterial clearance, we evaluated the cytotoxicity of the compound on MPI-2_eGFP mAMs. After seeding, we treated the cells with decreasing concentrations of **1f** (starting at 250 mM) and measured the eGFP fluorescence after 24 h. Notably, we did not observe a significant decrease in MPI-2_eGFP fluorescence, even at high concentrations (Figure S2D), thus suggesting that the compound did not affect cell viability.

**Figure 3:**
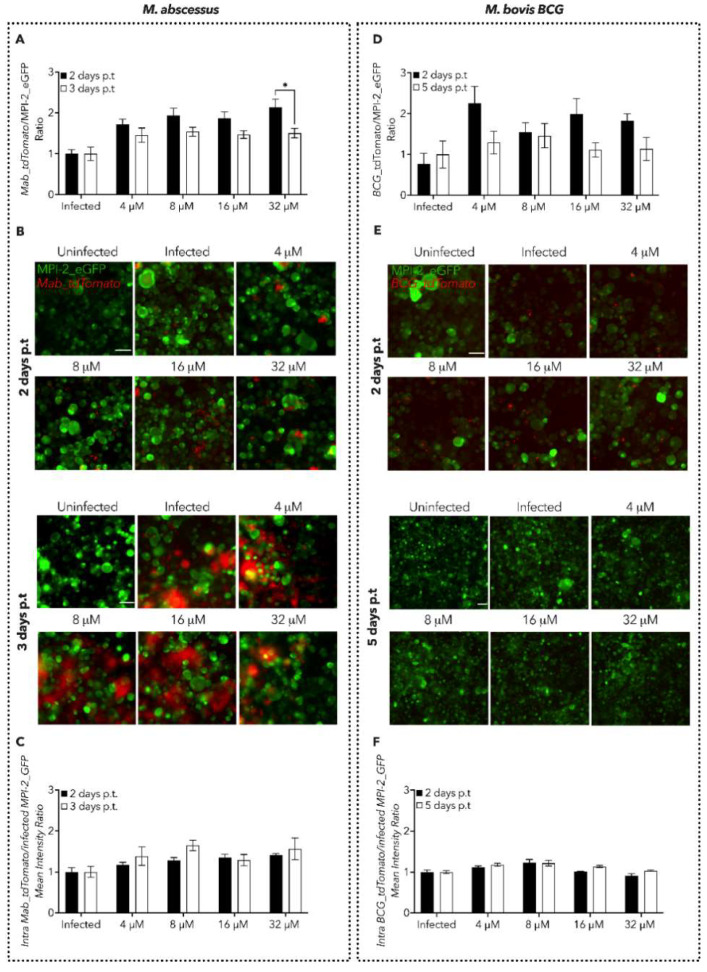
Characterization of **1f** antimycobacterial activity in mAM cells. (**A**,**D**) Spectrofluorometer (plate reader) measure of *Mab* and *M. bovis BCG* dissemination expressed as a ratio of either *Mab_tdTomato* (**A**) or *M. bovis BCG_tdTomato* (**D**) fluorescence over MPI-2_eGFP one. The measured time points are indicated in the legend. Black lines indicate the mean ± SEM. Significance by two-way ANOVA followed by Tukey’s multiple comparison test: * *p* = 0.0327. Data are from five independent experiments. (**B**,**E**) Representative images of mAMs infected with either *Mab_tdTomato* (**B**) or *M. bovis BCG_tdTomato* (**E**). eGFP and tdTomato fluorescence are merged. Scale bar: 50 µm. (**C**,**F**) Measure of *Mab* and *M. bovis BCG* intracellular dissemination at the single-cell level, expressed as a ratio obtained by dividing intracellular *Mab_tdTomato* (**A**) and *M. bovis BCG_tdTomato* (**D**) fluorescence over infected MPI-2_eGFP fluorescence. The measured time points are indicated in the legend. Black lines indicate the mean ± SEM. Data are from five independent experiments.

Next, we set up an in vitro plate assay where we used both bacilli and MPI-2 fluorescence as a proxy to examine either the progression and/or clearance of *Mab* and *M. bovis BCG* infection in its early and late stages (Figure 3). In detail, we infected MPI-2_eGFP cells with either *Mab_tdTomato* or *BCG_tdTomato* for 1 and 4 h, respectively, to allow pathogen phagocytosis by the mAMs. We then evaluated the antimycobacterial properties of **1f** by treating the infected MPI-2_eGFP mAMs with increasing concentrations of the compound, followed by both tdTomato and eGFP fluorescence quantification upon cell fixation.

To rapidly obtain enough data to analyze the feasibility of the new experimental protocol, we took advantage of the fast-growing *Mab*, which has a duplication time of around 5 h; hence, we decided to analyze the activity of **1f** at 2- and 3-days post-treatment (p.t) (Figure 3A–C). Spectrofluorometer-based quantifications suggested that the treatment with **1f** did not resolve *Mab* infection, as demonstrated by the level of fluorescence of both *Mab_tdTomato* (Figure S2A) and MPI-2_eGFP (Figure S2B). However, **1f** significantly reduced the *Mab_tdTomato* load 3-days p.t at the highest concentration tested (32 μM), with respect to the prior time point (Figure 2A). To add a further layer of precision to our quantification, the same samples were also imaged by microscopy to specifically collect information from infected cells only. Therefore, we quantified the intensity of the fluorescence of both eGFP and tdTomato inside the infected cells by using an ad hoc script for image analysis able to segment the individual cells and bacilli. Also, this approach did not display a decrease in the *Mab_tdTomato burden* (Figure 3A,C and Figure S2C,D).

Then, we treated MPI-2_eGFP cells infected with *M. bovis BCG_tdTomato* with **1f**. Differently from *Mab*, the tubercular *BCG* is a slow-growing mycobacterium (duplication time of ≈24 h); hence, we decided to assess the clearing efficiency of **1f** at 2 and 5 days p.t (Figure 3D–F and Figure S2E–H). Similarly to *Mab*, we did not detect a reduction in Tdtomato fluorescence nor an increase in eGFP following treatment with **1f** (Figure 3D and Figure S2E–H). This observation was further confirmed by spinning disk confocal microscopy analysis (Figure 3E,F).

Overall, despite the improved MIC_99_ of **1f**, we did not detect a significant efficacy in killing intracellular bacteria in in vitro macrophage infection models.

#### 2.2.3. Effect of 1f on the Viability of Blood Cells

**Figure 4:**
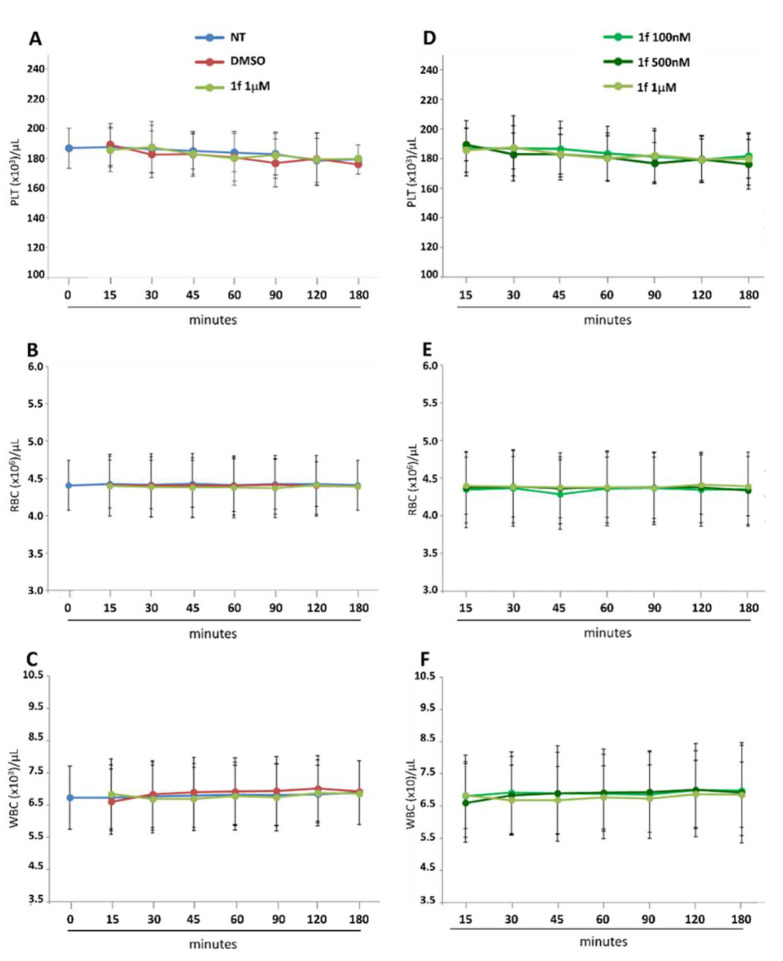
Effect of **1f** on blood cell count. Whole blood from five healthy volunteers was incubated with different concentrations of **1f** (100 nM, 500 nM and 1 µM) or with solvent (DMSO) up to 3 h at room temperature. Platelet (PLT; (**A**,**D**)), red blood cell (RBC; (**B**,**E**)) and white blood cell (WBC; (**C**,**F**)) count was evaluated at 0, 15, 30, 45, 60, 90, 120 min and 3 h after the addition of **1f** or solvent, using an automated hematology analyzer. All the data obtained were compared to untreated (NT) blood. Data are reported as count ± SD of each blood cell population.

To further confirm the negligible toxicity of **1f**, already demonstrated on mAMs, the effects of the compound against blood cells from healthy volunteers were assessed. For this purpose, whole blood was incubated with different concentrations of **1f**; then, cell count and platelet activation were analysed. We tested the cytotoxicity of **1f** against platelet (PLT), red blood cells (RBC), and white blood cells (WBC). PLT, RBC, and WBC counts, assessed in a 3 h timeframe after the addition of **1f** (1 µM), were like untreated (NT) and DMSO-treated whole blood (Figure 4A–C), indicating the absence of any specific effect of the compound on these parameters. No differences among the three concentrations of drug tested in the cell counts, at any time point, were observed (Figure 4D–F).

**Figure 5:**
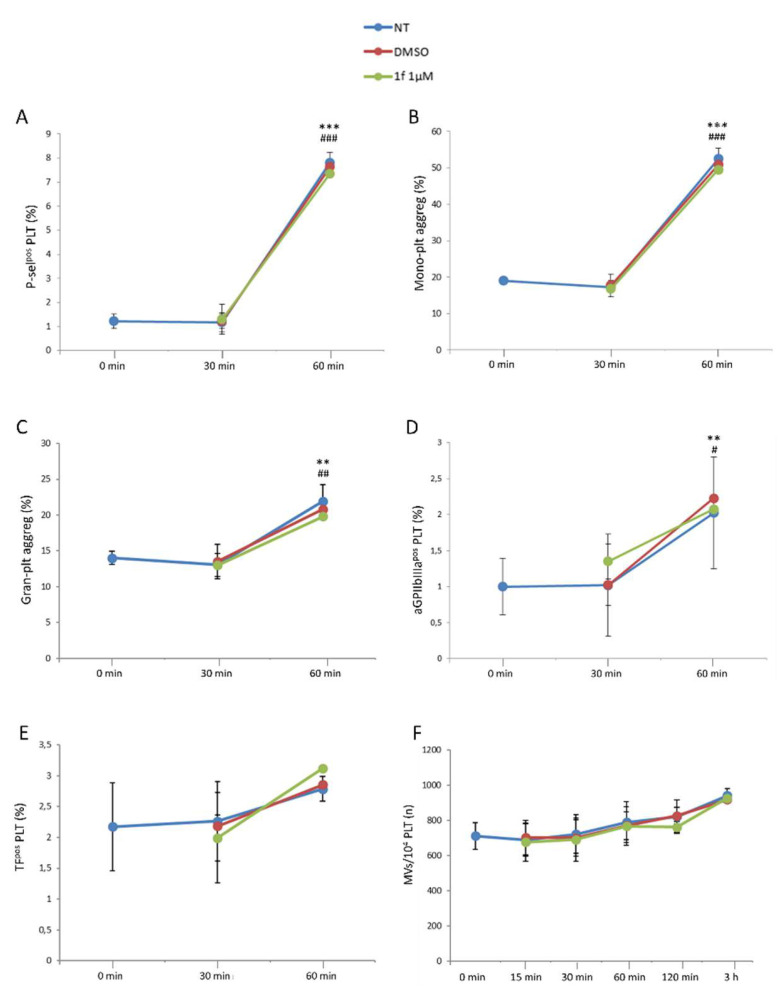
Effect of **1f** on platelet activation. Platelet-associated P-selectin expression (**A**) and the formation of monocyte-platelet (**B**) and granulocyte-platelet aggregates (**C**), as well as aGPIIbIIIa^pos^ (**D**) and Tissue Factor^pos^ (TF; (**E**)) platelets and the number of microvesicles/10,000 platelets (**F**) were assessed by flow cytometry in **1f**—(1 µM) or DMSO-treated whole blood. All the data obtained were compared to untreated (NT) whole blood. Data are reported as percentage ± SD of positive cells or number ± SD of microvesicles. Comparison between 0 min vs. 60 min: ** *p* < 0.01; *** *p* < 0.001. Comparison between 30 min vs. 60 min: ^#^ *p* < 0.05; ^##^ *p* < 0.01; ^###^ *p* < 0.001.

The expression of the conventional platelet activation markers, P-selectin (Figure 5A), monocyte-platelet and granulocyte-platelet aggregate formation (Figure 5B,C, respectively), as well as activated glycoprotein IIbIIIa (aGPIIbIIIa; Figure 5D) was not affected over the time by the drug at the highest concentration used. Similarly, platelet-associated Tissue Factor expression (TF; Figure 5E), reflecting that the platelet procoagulant phenotype, microvesicle formation (Figure 5F), and index of cell activation were not influenced by **1f**. As expected, the expression of all these markers increased over time but similarly in untreated, DMSO-, and drug-treated whole blood. Overall, these results highlight that **1f** does not affect blood cell count or ex vivo platelet activation, at the tested concentrations.

## 3. Discussion

Among our series of furan-based inhibitors of MbtI, *m*-cyano derivatives emerged as the most potent candidates. However, their activity was not optimal against mycobacteria in vitro, with MICs higher than 200 µM against the nonpathogenic *M. bovis BCG*. To address the potency issue, efforts were focused on the insertion of a side chain linked to the 5-position of the phenyl ring. A set of lipophilic moieties were connected by an ethereal bridge to improve the hydrophobicity of the compounds (cLogP values, calculated with DataWarrior [23], are provided in the SM). Indeed, excessive hydrophilicity was probably the cause of the lack of a solid correlation between the remarkable enzymatic activity and the antimycobacterial efficacy in vitro. Therefore, we maintained the cyano group in the *meta* position of the phenyl ring to ensure the efficiency in terms of enzymatic activity, while examining the effects of various lipophilic decorations on the antimycobacterial potency. The final goal was to identify the structural requirements needed to achieve both an efficient MbtI inhibition and potent antitubercular effect with this class of compounds. Hence, we set out to insert a phenoxy substituent (**1a**), and we were delighted to discover a promising MIC_99_ of 125 μM. This substituent led to an increase in the clogP from 1.9192 of the unsubstituted **I** to 3.3148 of **1a** (as calculated by OSIRIS DataWarrior v.5.5.0 [23]). Encouraged by the higher potency of **1a**, we decided to evaluate additional modifications of the side chain. With this compound as the starting point, we further explored the insertion of two or three carbon atoms on the lateral chain and obtained compounds **1b–d**. The gradual movement of the phenyl moiety away from the furan ring did not lead to an improvement in terms of antimycobacterial potential, while **1e**, functionalized with a cinnamic moiety, showed a comparable antitubercular activity (≈125 μM). Then, we explored the introduction of an oxygen, instead of the distal carbon of the alkyl chain, leading to **1f**, which displayed the best antimycobacterial activity detected so far for this class of compounds, with a MIC_99_ of 63 µM. This effect was positively related to the inhibition of iron uptake, as confirmed by the Universal CAS assay. The comparison between **1a** and **1f** suggested that further expansion of the side chain and introduction of a heteroatom might be beneficial for the activity: **1f** halved both its IC_50_ and MIC_99_ values, highlighting the better druggability of this compound with respect to our previous candidates.

Since the inhibition of mycobactin biosynthesis impairs mycobacterial growth in iron-limiting conditions, MbtI inhibitors should prevent the establishment of macrophage infection. Commonly used in vitro models for mycobacterial infection rely on the use of either immortalized cell lines (e.g., THP1 and Raw 264.7) or blood-derived primary macrophages (e.g., bone-marrow-derived macrophages). 

Nevertheless, despite the common biological function shared among these cell types, it has been shown that their diverse ontogeny may not appropriately reproduce tissue-specific cellular functions [24]. In this regard, *Mtb* infection occurs in the alveolar niche, where AMs are the primary cells encountering the pathogen and dictating the outcome of the infection. AMs exhibit a very specific and distinct phenotype, which depends on the unique lung microenvironment. Hence, the immunogenic response of AMs to pathogenic mycobacteria also diverges from that of other immune cells [25]. However, the reduced availability of AMs and their purity are two major limiting factors in their use for the investigation of mycobacteria–host interaction. To overcome such limitations, a recent murine embryonic-derived, self-renewing alveolar-like macrophage cellular model, namely the MPI-2 cell line, has been developed and characterized [21,22]. MPI-2 closely reproduce both the mAM-like phenotype and the immune response to the pathogen invasion, and they can be expanded and cultured in virtually unlimited amount. Thus, MPI-2 cells accurately replicate the mycobacterial–host crosstalk, representing the first choice for antimycobacterial drug-screening. For this reason, having established its improved inhibitory activity, **1f** was selected for further ex vivo studies using MPI-2 cells.

We first checked for the potential toxicity of this class of compounds, which had already been shown to be safe against MRC5 human fibroblast lung cells [18]. Here, we tested the cytotoxicity of **1f** against PLT, RBC, and WBC to ensure its biocompatibility with blood, considering that the alveolar area is heavily vascularized. Despite the tested concentration having to be reduced due to the sensitivity of blood cells to DMSO, our preliminary data show that this class of compounds is nontoxic and may be suitable for further testing in vivo. Similarly, **1f** did not show significant effects on mAMs up to 250 μM.

As these compounds are nontoxic against eukaryotic cells, we investigated the effects of **1f** in mAM infection models using *M. abscessus* and *M. bovis BCG*. The compound was assayed up to 32 μM, the minimal concentration that prevented the production of siderophores, according to the CAS assay. Unfortunately, despite its improved in vitro activity, **1f** showed only a modest effect in reducing the mycobacterial burden in mAMs at the highest concentration. There may be several reasons that could explain the lack of in vitro effectiveness of **1f** and that could help to further improve the efficiency of the compound. For instance, **1f** (i) could have a low permeability across the macrophage membrane, (ii) it may be inactivated or degraded within the host cells, (iii) or it could have issues in permeating the membrane of the endosome, where the bacilli are engulfed from the environment. It is also tempting to speculate that the limited activity of **1f** might depend on the uptake by the host-escaped extracellular bacterial load. In such a scenario, the limited extracellular iron-depletion pressure imposed by the AMs might only partially promote MbtI expression and/or activation, hence limiting **1f** antimicrobial activity.

Besides maintaining mycobacterial homeostasis, microenvironmental concentrations of iron are also known to govern innate immune responses, including macrophage M1- (pro-inflammatory) and M2- (anti-inflammatory) polarization. Although more in-depth studies are still needed, to date it has been shown that M1-like polarization appears to be associated with an increased expression of diverse Fe-regulated genes (e.g., Hamo, FtH/FtL [26,27]). Conversely, iron depletion could promote M2-like phenotype [28]. Hence, accounting for the role of iron in regulating macrophage function and pathogen dissemination, we could hypothesize that AM-like cells and mycobacteria might compete for the available iron. AMs could induce iron depletion in the mycobacteria to eliminate the intracellular bacterial burden; at the same time, the bacilli could sequester the iron through siderophore synthesis to induce iron depletion in the host, thus pushing mAM cells towards an M2-like phenotype, which is known to be more permissive. Nonetheless, further studies are required to evaluate a putative anti-inflammatory effect of **1f** on the host cells.

## 4. Material and Methods

### 4.1. Chemistry

All starting reagents and solvents were purchased from commercial suppliers (Sigma-Aldrich, St. Louis, MI, USA; FluoroChem, Hadfield, UK) and used as received. Anhydrous solvents were utilized without further drying. The course of the reactions was followed by thin-layer chromatography (TLC) using aluminum-backed Silica Gel 60 plates (0.2 mm; Merck, Darmstadt, Germany). Microwave-assisted reactions were carried out with a Biotage^®^ Initiator Classic (Biotage, Uppsala, Sweden). Crude products were purified by flash column chromatography on silica gel 60 (40–63 μm; Merck, Darmstadt, Germany) using the indicated solvent system. Melting points were determined in open capillary tubes with a Stuart SMP30 Melting Point Apparatus (Cole-Parmer Stuart, Stone, UK). All tested compounds were characterized by means of mono- and bi-dimensional NMR techniques, FT-IR, and ESI-MS. ^1^H and ^13^C NMR spectra were acquired at ambient temperature with a Varian Oxford 300 MHz instrument (Varian, Palo Alto, CA, USA) or a Bruker Avance 300 MHz instrument (Bruker, Billerica, MA, USA), operating at 300 MHz for ^1^H and 75 MHz for ^13^C. 2D NMR experiments were performed on a Bruker Avance Neo 400 MHz spectrometer. Chemical shifts are expressed in ppm (δ), and *J*-couplings are given in Hertz. The full decoupling mode was employed for ^13^C spectra when the relaxation times of the carbons did not allow for a sufficient resolution using the APT sequence. When necessary, the 2D-COSY and 2D-NOESY sequences were employed to unambiguously assign the hydrogen signals, while HSQC-DEPT experiments were performed to aid the assignment of ^13^C NMR signals. ATR-FT-IR spectra were acquired with a Perkin Elmer Spectrum One FT-IR (Perkin Elmer, Waltham, MA, USA), equipped with a Perkin Elmer Universal ATR sampling accessory consisting of a diamond crystal. Analyses were performed in a spectral region between 4000 and 650 cm^−1^ and analyzed by transmittance technique with 28 scansions and 4 cm^−1^ resolutions. MS analyses were carried out with a Thermo Fisher (Waltham, MA, USA) LCQ Fleet system, equipped with an ESI electrospray ionization source and an Ion Trap mass analyzer; ionization: ESI positive or ESI negative; capillary temperature: 250 °C; source voltage: 5.50 kV; source current: 4.00 μA; multipole 1 and 2 offset: −5.50 V and −7.50 V, respectively; intermultipole lens voltage: −16.00 V; trap DC offset voltage: −10.00 V. The purity (≥95%) of the tested compounds was assessed by means of reversed-phase HPLC (Waters, Milford, MA, USA) on a Phenomenex Luna^®^ 3 µm C18(2) 100 Å, 100 mm × 4.6 mm column (Phenomenex, Torrance, CA, USA) using the following operative conditions: mobile phase: 80:20 methanol/water + 0.05% TFA (isocratic mode); flow rate: 1 mL/min; detector λ: 254 nm; time: 20 min; temperature: 23 °C. The synthesis of the final compounds (**1a–j**) and ester intermediates (**2a–j**) is described in the following paragraphs. Proton and carbon assignments are based on 1D and 2D NMR data and are given for all final compounds; atom numbering is detailed in the SM. The preparation of the remaining synthetic precursors (**3**, **4a,e–j**, **5**) is reported in the SM. Compounds **2b–d** were obtained from commercially available bromo-derivatives.

***5-(3-Cyano-5-phenoxyphenyl)furan-2-carboxylic acid (1a).* General Procedure A.** The appropriate ester (43 mg, 0.135 mmol) was dissolved in a solution of THF/H_2_O 2:1 (1.0 mL). LiOH·H_2_O (17 mg, 0.407 mmol) was added while the solution was kept in an ice bath. The reaction mixture was stirred at r.t. for a variable time (1–6 h). THF was evaporated under reduced pressure, and the aqueous phase was brought to an acidic pH with 3 M HCl; the precipitated solid was filtered in vacuo and washed with small portions of a cyclohexane/EtOAc 6:4 mixture. Further purification strategies were adopted, when needed to afford the desired product. Starting compound: methyl 5-(3-benzyl-5-cyanophenyl)furan-2-carboxylate (**2a**). Time: 1 h. Yield: 80%. Aspect: white solid. Mp: 228 °C (dec.). TLC (DCM–MeOH 8:2): R_f_ = 0.41. ^1^H NMR (300 MHz, DMSO-*d*_6_) δ (ppm): 13.55–12.93 (br s exch. D_2_O, 1H, COOH), 8.05 (d, *J* = 1.5 Hz, 1H, H_7_), 7.64 (t, *J* = 1.9 Hz, 1H, H_11_), 7.47 (t, *J* = 1.7 Hz, 1H, H_9_), 7.43 (dd, *J* = 7.5, 1.7 Hz, 2H, H_14_, H_14′_), 7.36 (d, *J* = 3.7 Hz, 1H, H_3_), 7.32 (d, *J* = 3.7 Hz, 1 H, H_4_), 7.23 (t, *J* = 7.4 Hz, 1H, H_15_), 7.13 (dd, *J* = 7.5, 1.7 Hz, 2H, H_13_, H_13′_).^13^C NMR (75 MHz, DMSO-*d*_6_) δ (ppm): 159.47 C_10_, 158.32 COOH, 155.87 C_5_, 153.63 C_12_, 145.75 C_10_, 132.69 C_6_, 130.87 C_14_, C_14′_, 125.13 C_15_, 123.45 C_7_, 121.87 C_3_, 120.07 C_9_, 119.73 C_13_, C_13′_, 118.51 C_11_, 118.11 CN, 114.29 C_8_, 110.94 C_4_. FTIR (ATR): ν = 3116, 3086, 3063, 2956, 2924, 2919, 2852, 2233, 1732, 1693, 1592, 1569, 1519, 1438, 1417, 1363, 1298, 1216, 1030, 803 cm^−1^. ESI-MS (*m*/*z*) calcd. for C_18_H_11_NO_4_ 305.07, found 305.02 [M–H]^−^, 609.57 = [2M–H]^−^. HPLC purity: 96.7%.

***5-(3-(Benzyloxy)-5-cyanophenyl)furan-2-carboxylic acid (1b).*** The compound was obtained according to General Procedure A. Starting compound: methyl 5-(3-(benzyloxy)-5-cyanophenyl)furan-2-carboxylate (**2b**). Time: 90 min. Purification: trituration in cold DCM. Yield: 80%. Aspect: yellowish solid. Mp: 187 °C. TLC (DCM–MeOH 9:1): R_f_ = 0.20. ^1^H NMR (300 MHz, DMSO-*d*_6_) δ (ppm): 13.50–12.90 (br s exch. D_2_O, 1H, COOH), 7.84 (t, *J* = 1.4 Hz, 1H, H_7_), 7.71 (dd, *J* = 2.5, 1.4 Hz, 1H, H_11_), 7.55 (dd, *J* = 2.5, 1.4 Hz, 1H, H_9_), 7.50–7.45 (m, 2H, H_13_, H_13′_), 7.43–7.36 (m, 3H, H_14_, H_14′_, H_15_), 7.35 (d, *J* = 3.6 Hz, 1H, H_3_), 7.32 (d, *J* = 3.6 Hz, 1H, H_4_), 5.20 (s, 1H, CH_2_). ^13^C NMR (75 MHz, DMSO-*d*_6_) δ (ppm): 159.62 C_10_, 159.51 COOH, 154.00 C_5_, 145.81 C_2_, 136.58 C_6_, 132.19 C_12_, 128.98 C_14_, C_14′_, 128.61 C_15_, 128.45 C_13_, C_13′_, 120.88 C_7_, 119.96 C_3_, 118.61 CN, 118.29 C_9_, 115.97 C_11_, 113.71C_8_, 110.56 C_4_, 70.47 CH_2_. FTIR (ATR): ν = 3489, 3405, 3121, 3087, 2968, 2935, 2882, 2232, 1679, 1594, 1517, 1436, 1366, 1329, 1302, 1288, 1216, 1167, 1048, 1025, 963, 761 cm^−1^. ESI-MS (*m*/*z*) calcd. for C_19_H_13_NO_4_ 319.08, found 318.4 [M-H]^−^. HPLC purity: 97.7%.

***5-(3-Cyano-5-phenethoxyphenyl)furan-2-carboxylic acid (1c).*** The compound was obtained according to General Procedure A. Starting compound: methyl 5-(3-cyano-5-phenethoxyphenyl)furan-2-carboxylate (**2c**). Time: 4 h. Purification: recrystallization from DCM/hexane. Yield: 85%. Aspect: white solid. Mp: 202 °C. TLC (DCM–MeOH 9:1): R_f_ = 0.23. ^1^H NMR (300 MHz, DMSO-*d*_6_) δ (ppm): 13.95–12.64 (br s exch. D_2_O, 1H, COOH), 7.80 (t, *J* = 1.4 Hz, 1H, H_7_), 7.58 (dd, *J* = 2.5, 1.5 Hz, 1H, H_11_), 7.46 (dd, *J* = 2.5, 1.5 Hz, 1H, H_9_), 7.38–7.26 (m, 6H, H_14_, H_14′_, H_13_, H_13′_, H_4_, H_3_), 7.25–7.17 (m, 1H, H_15_), 4.33 (t, *J* = 6.8 Hz, 2H, CH_2_O), 3.06 (t, *J* = 6.8 Hz, 2H, CH_2_). ^13^C NMR (75 MHz, DMSO-*d*_6_) δ (ppm): 159.65 C_10_, 159.60 COOH, 153.98 C_5_, 145.80 C_2_, 138.48 C_12_, 132.14 C_6_, 129.45 C_14_, C_14′_, 128.80 C_13_, C_13′_, 126.82 C_15_, 120.69 C_7_, 119.89 C_3_, 118.60 C_9_, 115.55 C_11_, 118.60 CN, 113.73 C_8_, 110.54 C_4_, 69.40 CH_2_O, 35.14 CH_2_. FTIR (ATR): ν = 3087, 3059, 3026, 2976, 2947, 2932, 2841, 2235, 1696, 1679, 1607, 1593, 1522, 1436, 1365, 1334, 1304, 1272, 1219, 1163, 1062, 1030, 959, 754 cm^−1^. ESI-MS (*m*/*z*) calcd. for C_20_H_15_NO_4_ 333.10, found 333.01 [M-H]^−^, 665.66 [2M-H]^−^. HPLC purity: 98.5%.

***5-(3-Cyano-5-(3-phenylpropoxy)phenyl)furan-2-carboxylic acid (1d).*** The compound was obtained according to General Procedure A. Starting compound: methyl 5-(3-cyano-5-(3-phenylpropoxy)phenyl)furan-2-carboxylate (**2d**). Time: 2 h. Purification: flash column chromatography (DCM–MeOH 9:1). Yield: 80%. Aspect: white solid. Mp: 186 °C. TLC (DCM–MeOH 9:1): R_f_ = 0.20. ^1^H NMR (300 MHz, DMSO-*d*_6_) δ (ppm): 13.70–12.70 (br s exch. D_2_O, 1H, COOH), 7.81 (t, *J* = 1.4 Hz, H_7_), 7.60 (dd, *J* = 2.5, 1.4 Hz, 1H, H_11_), 7.44 (dd, *J* = 2.5, 1.4 Hz, 1H, H_9_,), 7.34 (d, *J* = 3.6 Hz, 1H, H_3_)_,_ 7.30 (d, *J* = 3.6 Hz, 1H, H_4_), 7.30–7.12 (m, 5H, H_16_, H_16__′_, H_17_, H_17__′_, H_18_), 4.09 (t, *J* = 6.2, 2H, H_12_), 2.74 (dd, *J* = 8.6, 6.5 Hz, 2H, H_14_), 2.02 (m, 2H, H_13_). ^13^C NMR (75 MHz, DMSO-*d*_6_) δ (ppm): 159.79 C_10_, 159.68 COOH, 153.97 C_5_, 145.89 C_2_, 141.69 C_15_, 132.17 C_6_, 128.81 C_17_, C_17__′_ C_16_, C_16__′_, 126.33 C_18_, 120.68 C_7_, 119.84 C_3_, 118.64 CN, 117.79 C_9_, 115.63 C_11_, 113.74 C_8_, 110.51 C_4_, 68.12 C_12_, 31.81 C_14_, 30.62 C_13_. FTIR (ATR): ν = 3403, 3120, 3084, 3026, 2951, 2884, 2228, 1680, 1592, 1517, 1429, 1363, 1333, 1215, 1164, 1039, 958, 869, 797, 699 cm^−1^. ESI-MS (*m*/*z*) calcd. for C_21_H_17_NO_4_ 347.12, found 346.61 [M-H]^−^, 693.37 [2M-H]^−^. HPLC purity: 95.1%.

***(E)-5-(3-(Cinnamyloxy)-5-cyanophenyl)furan-2-carboxylic acid (1e).*** The compound was obtained according to General Procedure A. Starting compound: (*E*)-methyl 5-(3-(cinnamyloxy)-5-cyanophenyl)furan-2-carboxylate (**2e**). Time: 1 h. Yield: 82%. Aspect: white solid. Mp: 170 °C. TLC (DCM–MeOH 9:1): R_f_ = 0.14. ^1^H NMR (300 MHz, DMSO-*d*_6_) δ (ppm): 13.88–12.65 (br s exch. D_2_O, 1H, COOH), 7.83 (t, *J* = 1.4 Hz, H_7_), 7.67 (dd, *J* = 2.1, 1.5 Hz, 1H, H_11_), 7.55–7.44 (m, 3H, H_9_, H_16_, H_16′_), 7.41–7.16 (m, 5H, H_3_, H_4_, H_17_, H_17′_, H_18_), 6.81 (d, *J* = 16.0 Hz, 1H, H_14_), 6.51 (dt, *J* = 16.0, 5.8 Hz, 1H, H_13_), 4.86 (dd, *J* = 5.9, 1.4 Hz, 2H, H_12_). ^13^C NMR (75 MHz, DMSO-*d*_6_) δ (ppm): 159.16 C_10_, 158.89 COOH, 153.31 C_5_, 145.70 C_2_, 135.96 C_15_, 133.05 C_14_, 131.76 C_6_, 128.60 C_17_, C_17′_, 127.93 C_18_, 126.46 C_16_, C_16′_, 123.92 C_13_, 120.28 C_7_, 119.08 C_3_, 118.09 CN, 117.73 C_9_, 115.34 C_11_, 113.21 C_8_, 109.95 C_4_, 68.80 C_12_. FTIR (ATR): ν = 3391, 3019, 3080, 3027, 2929, 2667, 2576, 2232, 1678, 1593, 1569, 1519, 1436, 1364, 1331, 1269, 1168, 1146, 1030, 961, 862, 806, 692 cm^−1^. ESI-MS (*m*/*z*) calcd. for C_21_H_15_NO_4_ 345.35, found 344.83 [M-H]^−^, 689.40 [2M-H]^−^. HPLC purity: 95.6%.

***5-(3-Cyano-5-(2-phenoxyethoxy)phenyl)furan-2-carboxylic acid (1f).*** The compound was obtained according to General Procedure A. Starting compound: methyl 5-(3-cyano-5-(2-phenoxyethoxy)phenyl)furan-2-carboxylate (**2f**). Time: 3 h. Yield: 92%. Aspect: white solid. Mp: 195 °C. TLC (DCM–MeOH 9:1): R_f_ = 0.18. ^1^H NMR (300 MHz, DMSO-*d*_6_) δ (ppm): 13.07–12.92 (br s exch. D_2_O, 1H, COOH), 7.83 (t, *J* = 1.4 Hz, 1H, H_7_), 7.69 (dd, *J* = 2.5, 1.5 Hz, 1H, H_11_), 7.52 (dd, *J* = 2.5, 1.5 Hz, 1H, H_9_), 7.35 (d, *J* = 3.6 Hz, 1H, H_3_), 7.32 (d, *J* = 3.6 Hz, 1H, H_4_), 7.30–7.24 (m, 2H, H_16, 16__′_), 7.02 − 6.89 (m, 3H, H_15_, H_15__′_, H_17_), 4.46 (m, 2H, H_12_ or H_13_), 4.32 (m, 2H, H_13_ or H_12_). ^13^C NMR (75 MHz, DMSO-*d*_6_) δ (ppm): 159.56 C_10_, 159.54 C_14_, 158.66 COOH, 154.07 C_5_, 145.65 C_2_, 132.19 C_6_, 129.97 C_16_, C_16__′_, 121.28 C_7_, 120.89 C_3_, 119.99 C_9_, 118.55 CN, 118.18 C_11_, 115.80 C_17_, 114.98 C_15_, C_15__′_, 113.77 C_8_, 110.54 C_4_, 67.77 CH_2_, 66.50 CH_2_. FTIR (ATR): ν = 3115, 3082, 2973, 2941, 2878, 2695, 2571, 2521, 2234, 1690, 1675, 1609, 1594, 1520, 1488, 1447, 1417, 1339, 1300, 1209, 1167, 1055, 862, 750 cm^−1^. ESI-MS (*m*/*z*) calcd. for C_20_H_15_NO_5_ 349.34, found 348.40 [M-H]^−^, 697.22 [2M-H]^−^. HPLC purity: 98.4%.

***5-(3-Cyano-5-(2-(phenylamino)ethoxy)phenyl)furan-2-carboxylic acid (1g).*** The compound was obtained according to General Procedure A. Starting compound: methyl 5-(3-cyano-5-(2- (phenylamino)ethoxy)phenyl)furan-2-carboxylate (**2g**). Time: 4 h. Yield: 81%. Aspect: white solid. Mp: 140 °C. TLC (DCM–MeOH 9:1): R_f_ = 0.44. ^1^H NMR (300 MHz, DMSO-*d*_6_) δ (ppm): 7.80 (t, *J* = 1.4 Hz, 1H, H_7_), 7.62 (t, *J* = 2.0 Hz, 1H, H_11_), 7.43 (dd, *J* = 2.0, 1.4 Hz 1H, H_9_), 7.28 (d, 1H, *J* = 3.6 Hz, H_3_), 7.18 (d, *J* = 3.6 Hz, 1H, H_4_), 7.11–7.01 (m, 2H, H_16_, H_16′_), 6.62 (m, 2H, H_15_, H_15′_) 6.53 (t, *J* = 7.2 Hz, 1H, H_17_), 5.78 (br s exch. D_2_O, 1H, NH), 4.24 (t, *J* = 5.4 Hz, 2H, H_12_), 3.43 (t, partially overlapped with DMSO water peak, *J* = 5.4 Hz, 2H, H_13_). ^13^C NMR (75 MHz, DMSO-*d*_6_) δ (ppm): 160.07 C_10_, 159.70 COOH, 158.97 C_14_, 153.14 C_5_, 148.90 C_2_, 132.44 C_6_, 129.38 C_16_, C_16′_, 120.60 C_7_, 118.65 CN, 118.61 C_3_, 117.68 C_9_, 116.33 C_11_, 115.44 C_17_, 113.62 C_8_, 112.53 C_15_, C_15′_, 110.37 C_4_, 67.71 C_12_, 42.46 C_13_. FTIR (ATR): ν = 3385, 3115, 3055, 3027, 2962, 2928, 2853, 2647, 2554, 2231, 1643, 1594, 1517, 1435, 1367, 1326, 1259, 1154, 1092, 1056, 1023, 962, 868, 798, 692 cm^−1^. ESI-MS (*m*/*z*) calcd. for C_20_H_16_N_2_O_4_ 348.11, found 347.98 [M-H]^−^, 695.67 [2M-H]^−^. HPLC purity: 97.6%.

***5-(3-Cyano-5-(naphthalen-2-yloxy)phenyl)furan-2-carboxylic acid (1h).*** The compound was obtained according to General Procedure A. Starting compound: methyl 4-(3-cyano-5-(naphthalen-2-yloxy)phenyl)cyclopenta-1,3-dienecarboxylate (**2h**). Time: 1 h. Yield: 79%. Aspect: white solid. Mp: 226 °C. TLC (DCM–MeOH 9:1): R_f_ = 0.16. ^1^H NMR (300 MHz, DMSO-*d*_6_) δ (ppm): 13.30 (s exch. D_2_O, 1H, COOH), 8.08–8.07 (m, 1H, H_7_), 8.02 (d, *J* = 8.9, 1H, H_18_), 7.99–7.91 (m, 1H, H_15_), 7.90–7.84 (m, 1H, H_15_), 7.75 (t, *J* = 2.0 Hz, 1H, H_11_), 7.58 (t, *J* = 1.9 Hz, 1H, H_9_), 7.56 (d, *J* = 2.5 Hz, 1H, H_19_), 7.55–7.45 (m, 2H, H_16_, H_17_), 7.38–7.34 (m, 2H, H_13_, H_3_), 7.30 (d, *J* = 3.5 Hz, 1H, H_4_). ^13^C NMR (75 MHz, DMSO-*d*_6_) δ (ppm): 159.95 C_10_, 158.62 COOH, 154.22 C_12_, 153.94 C_5_, 146.41 C_2_, 134.78 C_20_, 133.21 C_6_, 131.41 C_14_, 131.16 C_21_, 128.63 C_18_, 128.22 C_5_, 127.72 C_16_, 126.23 C_17_, 124.07 C_7_, 122.68 C_9_, 120.51 C_13_, 120.34 C_3_, 119.38 C_11_, 118.56 CN, 115.70 C_19_, 114.77 C_8_, 111.40 C_4_. FTIR (ATR): ν = 3117, 3057, 2962, 2919, 2232, 1697, 1591, 1570, 1520, 1418, 1362, 1296, 1214, 1029, 806 cm^−1^. ESI-MS (*m*/*z*) calcd. for C_22_H_13_NO_4_ 355.35, found 354.92 [M-H]^−^, 709.41 [2M-H]^−^. HPLC purity: 97.9%.

***5-(3-Cyano-5-(quinolin-7-yloxy)phenyl)furan-2-carboxylic acid (1i).*** The compound was obtained according to General Procedure A. Starting compound: methyl 5-(3-cyano-5-(quinolin-7-yloxy)phenyl)furan-2-carboxylate (**2i**). Time: 3 h. Yield: 89%. Aspect: yellow solid. Mp: 263 °C (dec.). TLC (DCM–MeOH 9:1): R_f_ = 0.12. ^1^H NMR (300 MHz, DMSO-*d*_6_) δ (ppm): 13.26 (s exch. D_2_O, 1H, COOH), 8.87 (dd, *J* = 4.3, 1.7 Hz, 1H, H_17_), 8.39 (dd, *J* = 8.3, 1.8 Hz, 1H, H_15_), 8.12 (d, *J* = 1.5 Hz, 1H, H_7_), 8.08 (d, *J* = 8.7 Hz, 1H, H_14_), 7.83 (t, *J* = 1.9 Hz, 1H, H_11_), 7.69 (t, *J =* 1.8 Hz, 1H, H_9_), 7.53–7.43 (m, 3H, H_13_, H_16_, H_19_), 7.39 (d, *J* = 3.7 Hz, 1H, H_3_), 7.32 (d, *J* = 3.7 Hz, 1H, H_4_). ^13^C NMR (75 MHz, DMSO-*d*_6_) δ (ppm): 159.45 C_10_, 157.41 COOH, 157,16 C_12_, 153.59 C_5_, 151.80 C_17_, 149.18 C_20_, 145.76 C_2_, 136.44 C_15_, 132.87 C_6_, 131.05 C_14_, 125.48 C_21_, 124.27 C_19_, 123.05 C_13_, 121.19 C_3_, 120.41 C_7_, 120.10 C_9_, 119.79 C_16_, 118. 04 CN, 115.57 C_11_, 114. 51 C_8_, 111.06 C_4_. FTIR (ATR): ν = 3268, 3118, 3078, 2919, 2852, 2232, 1733, 1692, 1657, 1593, 1571, 1520, 1505, 1441, 1416, 1363, 1294, 1257, 1213, 1164, 1093, 1015, 798 cm^−1^. ESI-MS (*m*/*z*) calcd. for C_21_H_12_N_2_O_4_ 356.08, found 357.37 [M+H]^+^. HPLC purity: 96.5%.

***5-(3-Cyano-5-(naphthalen-2-ylmethoxy)phenyl)furan-2-carboxylic acid (1j).*** The compound was obtained according to General Procedure A. Starting compound: methyl 5-(3-cyano-5-(naphthalen-2-ylmethoxy)phenyl)furan-2-carboxylate (**2j**). Time: 6 h. Yield: 85%. Aspect: white solid. Mp: 209 °C (dec.). TLC (DCM–MeOH 7:3): R_f_ = 0.26. ^1^H NMR (300 MHz, DMSO-*d*_6_) δ (ppm): 8.08–8.01 (m, 1H, H_19_), 8.00–7.89 (m, 3H, H_14_, H_15_, H_18_), 7.85 (t, *J* = 1.4 Hz, 1H, H_7_), 7.79 (dd, *J* = 2.4, 1.4, Hz, 1H, H_11_), 7.62 (dd, *J* = 8.4, 1.7 Hz, 1H, H_13_), 7.59 (dd, *J* = 2.5, 1.3 Hz, 1H, H_9_), 7.58–7.51 (m, 2H, H_17_, H_16_), 7.36 (d, *J* = 3.6 Hz, 1H, H_4_), 7.28 (d, *J* = 3.5 Hz, 1H, H_3_). ^13^C NMR (75 MHz, DMSO-*d*_6_) δ (ppm): 159.82 C_10_, 159.53 COOH, 153.56 C_5_, 146.87 C_2_, 134.22 C_20_, 133.22 C_6_, 133.10 C_12_, 132.40 C_21_, 128.61 C_14_, 128.30 C_15/18_, 128.07 C_18/15_, 127.07 C_19_, 126.84 C_16/17_, 126.72 C_17/16_, 126.22 C_13_, 120.89 C_7_, 119.24 C_3_, 118.62 CN, 118.20 C_9_, 116.01 C_11_, 113.70 C_8_, 111.50 C_4_, 70.60 CH_2_. FTIR (ATR): ν = 3383, 3262, 3120, 3060, 2923, 2232, 1692, 1658, 1593, 1572, 1520, 1441, 1363, 1296, 1214, 1029, 803 cm^−1^. ESI-MS (*m*/*z*) calcd. for C_23_H_15_NO_4_ 369.37, found 368.65 [M-H]^−^, 737.06 [2M-H]^−^. HPLC purity: 96.1%.

***Methyl 5-(3-benzyl-5-cyanophenyl)furan-2-carboxylate (2a).*General Procedure B.** The appropriate bromo-derivative (70 mg, 0.255 mmol), (5-(methoxycarbonyl)furan-2-yl)boronic acid (56 mg, 0.330 mmol) and bis(triphenylphosphine)palladium dichloride (9 mg, 0.013 mmol) were dissolved in dry 1,4-dioxane (1.5 mL) under N_2_ atmosphere. A 2M Na_2_CO_3_ solution (0.26 mL, 0.255 mmol) was added, and the resulting mixture was stirred in a microwave synthesizer at 60 °C for 80 min. The reaction mixture was filtered on a pad of celite, diluted with water (2 mL), and extracted with EtOAc (3 × 3 mL). The organic layers were washed with brine, dried over anhydrous Na_2_SO_4_, filtered, and concentrated under reduced pressure. The crude product was purified by flash column chromatography (cyclohexane–EtOAc 8:2) to afford pure **2a** as a white solid. Starting compound: 3-bromo-5-phenoxybenzonitrile (**4a**). Yield: 55%. Mp: 131 °C. TLC (cyclohexane–EtOAc 8:2): R_f_ = 0.39. ^1^H NMR (300 MHz, CDCl_3_) δ (ppm): 7.78–7.74 (m, 1H, H_Ar_), 7.65–7.60 (m, 1H, H_Ar_), 7.47–7.37 (m, 3H, H_Ar_), 7.28–7.19 (m, partially overlapped with solvent peak, 4H, H_Ar_), 7.14–7.11 (m, 1H, H_Ar_), 7.09–7.02 (m, 2H, H_Ar_), 6.79 (d, *J* = 3.7 Hz, 1H, H_Ar_), 3.92 (s, 3H, OCH_3_).

***Methyl 5-(3-(benzyloxy)-5-cyanophenyl)furan-2-carboxylate (2b).* General Procedure C.** Methyl 5-(3-cyano-5-hydroxyphenyl)furan-2-carboxylate (**3**, 150 mg, 0.620 mmol) was suspended in dry acetone (3.05 mL) under a nitrogen atmosphere. Oven-dried K_2_CO_3_ (343 mg, 2.48 mmol) was added, and the mixture was stirred for 10 min. Then, a solution of the suitable bromo-derivative (192 mg, 1.12 mmol) in dry acetone (0.76 mL) was added, and the reaction mixture was heated at reflux for a variable time for each substrate (3–72 h). Then, the suspension was filtered in vacuo to remove K_2_CO_3_, and acetone was evaporated under reduced pressure. The crude product was purified to afford the desired product. Starting compound: (bromomethyl)-benzene. Time: 3 h. Purification: crystallization from DCM/hexane. Yield: 75%. Aspect: off-white solid. Mp: 144 °C. TLC (cyclohexane–EtOAc 8:2): R_f_ = 0.41. ^1^H NMR (300 MHz, CDCl_3_) δ (ppm): 7.64 (t, *J* = 1.4 Hz, 1H, H_Ar_), 7.60 (dd, *J* = 2.5, 1.4 Hz, 1H, H_Ar_), 7.49–7.32 (m, 5H, H_Ar_), 7.29–7.23 (m, partially overlapped with solvent peak, 1H, H_Ar_), 7.18 (dd, *J* = 2.5, 1.4 Hz, 1H, H_Ar_), 6.79 (d, *J* = 3.6 Hz, 1H, H_Ar_), 5.14 (s, 2H, OCH_2_), 3.93 (s, 3H, OCH_3_).

***Methyl 5-(3-cyano-5-phenethoxyphenyl)furan-2-carboxylate (2c).*** The compound was obtained according to General Procedure C. Starting compound: (2-bromoethyl)-benzene. Time: 72 h. Purification: flash column chromatography (cyclohexane–EtOAc 8:2). Yield: 35%. Aspect: pearl-white solid. Mp: 150 °C. TLC (cyclohexane–EtOAc 8:2): R_f_ = 0.40. ^1^H NMR (300 MHz, CDCl_3_) δ (ppm): 7.60 (t, *J* = 1.4 Hz, 1H, H_Ar_), 7.49 (dd, *J* = 2.5, 1.4 Hz, 1H, H_Ar_), 7.39–7.27 (m, partially overlapped with solvent peak, 5H, H_Ar_), 7.25 (d, *J* = 3.7 Hz, 1H, H_Ar_), 7.09 (dd, *J* = 2.5, 1.4 Hz, 1H, H_Ar_), 6.79 (d, *J* = 3.7 Hz, 1H, H_Ar_), 4.25 (t, *J* = 6.9 Hz, 2H, OCH_2_), 3.93 (s, 3H, OCH_3_), 3.14 (t, *J* = 6.9 Hz, 2H, CH_2_).

***Methyl 5-(3-cyano-5-(3-phenylpropoxy)phenyl)furan-2-carboxylate (2d).*** The compound was obtained according to General Procedure C. Starting compound: (3-propyl)benzene. Time: 16 h. Purification: flash column chromatography (cyclohexane–EtOAc 8:2). Yield: 35%. Aspect: yellowish solid. Mp: 90 °C. TLC (cyclohexane–EtOAc 8:2): R_f_ = 0.39. ^1^H NMR (300 MHz, CDCl_3_) δ (ppm): 7.61 (t, *J* = 1.4 Hz, 1H, H_Ar_), 7.50 (dd, *J* = 2.4, 1.4 Hz, 1H, H_Ar_), 7.38–7.16 (m, partially overlapped with solvent peak, 6H, H_Ar_), 7.08 (dd, *J* = 2.4, 1.4 Hz, 1H, H_Ar_), 6.79 (d, *J* = 3.6 Hz, 1H, H_Ar_), 4.03 (t, *J* = 6.2 Hz, 2H, OCH_2_), 3.93 (s, 3H, OCH_3_), 2.83 (t, *J* = 7.5 Hz, 2H, CH_2_), 2.23–2.07 (m, 2H, CH_2_).

***(E)-Methyl 5-(3-(cinnamyloxy)-5-cyanophenyl)furan-2-carboxylate (2e).*** The compound was obtained according to General Procedure C. Starting compound: (*E*)-(3-bromoprop-1-en-1-yl)benzene (**4e**). Time: 4 h. Purification: trituration in cold Et_2_O. Yield: 40%. Aspect: white solid. Mp: 129 °C. TLC (cyclohexane–EtOAc 8:2): R_f_ = 0.33. ^1^H NMR (300 MHz, CDCl_3_) δ (ppm): 7.64 (t, *J* = 1.4 Hz, 1H, H_Ar_), 7.58 (dd, *J* = 2.5, 1.4 Hz, 1H, H_Ar_), 7.47–7.39 (m, 2H, H_Ar_), 7.40–7.22 (m, partially overlapped with solvent peak, 4H, H_Ar_), 7.17 (dd, *J* = 2.5, 1.4 Hz, 1H, H_Ar_), 6.83–6.73 (m, 2H, H_Ar_), 6.39 (dt, *J* = 16.0, 5.8 Hz, 1H, CH), 4.78 (dd, *J* = 5.8, 1.5 Hz, 2H, CH_2_), 3.93 (s, 3H, OCH_3_).

***Methyl 5-(3-cyano-5-(2-phenoxyethoxy)phenyl)furan-2-carboxylate (2f*).** The compound was obtained according to General Procedure B. Starting compound: 3-bromo-5-(2-phenoxyethoxy)benzonitrile (**4f**). Purification: flash column chromatography (cyclohexane–EtOAc 8:2). Yield: 80%. Aspect: white solid. Mp: 134 °C. TLC (cyclohexane–EtOAc 8:2): R_f_ = 0.37. ^1^H NMR (300 MHz, CDCl_3_) δ (ppm): 7.66 (t, *J* = 1.5 Hz, 1H, H_Ar_), 7.59 (dd, *J* = 2.5, 1.5 Hz, 1H, H_Ar_), 7.38–7.23 (m, partially overlapped with solvent peak, 3H, H_Ar_), 7.18 (dd, *J* = 2.5, 1.5 Hz, 1H, H_Ar_), 7.07–6.92 (m, 3H, H_Ar_), 6.81 (d, *J* = 3.6 Hz, 1H, H_Ar_), 4.44–4.31 (m, 4H, CH_2_), 3.93 (s, 3H, OCH_3_).

***Methyl 5-(3-cyano-5-(2-(phenylamino)ethoxy)phenyl)furan-2-carboxylate (2g).*** The compound was obtained according to General Procedure B. Starting compound: 3-bromo-5-(2-(phenylamino)ethoxy)benzonitrile (**4g**). Purification: flash column chromatography (cyclohexane–EtOAc 7:3), recrystallization from DCM/hexane. Yield: 40%. Aspect: brownish solid. Mp: 160 °C. TLC (cyclohexane–EtOAc 8:2): R_f_ = 0.20. ^1^H NMR (300 MHz, CDCl_3_) δ (ppm): 7.64 (t, *J* = 1.4 Hz, 1H, H_Ar_), 7.53 (dd, *J* = 2.5, 1.4 Hz, 1H, H_Ar_), 7.32–7.16 (m, partially overlapped with solvent peak, 3H, H_Ar_), 7.12 (dd, *J* = 2.5, 1.4 Hz, 1H, H_Ar_), 6.84–6.63 (m, 4H, H_Ar_), 4.24 (t, *J* = 5.2 Hz, 2H, CH_2_), 4.07 (br s exch. D_2_O, 1H, NH), 3.93 (s, 3H, OCH_3_), 3.67–3.52 (m, 2H, CH_2_).

***Methyl 5-(3-cyano-5-(naphthalen-2-yloxy)phenyl)furan-2-carboxylate (2h)***. The compound was obtained according to General Procedure B. Starting compound: 3-bromo-5-(naphthalen-2-yloxy)benzonitrile (**4h**). Purification: flash column chromatography (cyclohexane–EtOAc 8:2). Yield: 35%. Aspect: white solid. Mp: 155 °C. TLC (cyclohexane–EtOAc 8:2): R_f_ = 0.35. ^1^H NMR (300 MHz, CDCl_3_) δ (ppm): 7.96–7.83 (m, 2H, H_Ar_), 7.83–7.74 (m, 2H, H_Ar_), 7.71–7.65 (m, 1H, H_Ar_), 7.58–7.40 (m, 3H, H_Ar_), 7.30–7.17 (m, partially overlapped with solvent peak, 3H, H_Ar_), 6.79 (d, *J* = 3.6 Hz, 1H, H_Ar_), 3.91 (s, 3H, OCH_3_).

***Methyl 5-(3-cyano-5-(quinolin-7-yloxy)phenyl)furan-2-carboxylate (2i).*** The compound was obtained according to General Procedure B. Starting compound: 3-bromo-5-(quinolin-7-yloxy)benzonitrile (**4i**). Purification: flash column chromatography (cyclohexane–EtOAc 6:4). Yield: 40%. Aspect: white solid. Mp: 142 °C. TLC (cyclohexane–EtOAc 6:4): R_f_ = 0.30. ^1^H NMR (300 MHz, CDCl_3_) δ (ppm): 8.92 (dd, *J* = 4.5, 1.7 Hz, 1H, H_Ar_), 8.33 (d, *J* = 8.2 Hz, 1H, H_Ar_), 7.95 (d, *J* = 8.9 Hz, 1H, H_Ar_), 7.87 (t, *J* = 1.4 Hz, 1H, H_Ar_), 7.80–7.70 (m, 2H, H_Ar_), 7.50 (dd, *J* = 8.2, 4.5 Hz, 1H, H_Ar_), 7.42 (dd, *J* = 8.9, 2.3 Hz, 1H, H_Ar_), 7.30 (dd, *J* = 2.3, 1.4 Hz, 1H, H_Ar_), 7.28–7.22 (m, partially overlapped with solvent peak, 1H, H_Ar_), 6.84 (d, *J* = 3.6 Hz, 1H, H_Ar_), 3.91 (s, 3H, OCH_3_).

***Methyl 5-(3-cyano-5-(naphthalen-2-ylmethoxy)phenyl)furan-2-carboxylate (2j).*** The compound was obtained according to General Procedure C. Starting compound: 2-(bromomethyl)naphthalene (**4j**). Time: 5 h. Purification: trituration in cold Et_2_O. Yield: 66%. Aspect: white solid. Mp: 195 °C (dec.). TLC (cyclohexane–EtOAc 8:2): R_f_ = 0.28. ^1^H NMR (300 MHz, CDCl_3_) δ (ppm): 7.97–7.80 (m, 4H, H_Ar_), 7.70–7.60 (m, 2H, H_Ar_), 7.57–7.48 (m, 3H, H_Ar_), 7.28–7.24 (m, partially overlapped with solvent peak, 1H, H_Ar_), 7.22 (dd, *J* = 2.4, 1.4 Hz, 1H, H_Ar_), 6.80 (d, *J* = 3.6 Hz, 1H, H_Ar_), 5.31 (s, 2H, OCH_2_), 3.93 (s, 3H, OCH_3_).

### 4.2. Biological Activities

#### 4.2.1. MbtI Enzymatic Assays

Recombinant *M. tuberculosis* MbtI was produced and purified, and enzyme activity determined as previously reported [9]. All the reactions were performed at 37 °C, in a final volume of 400 μL of 50 mM Hepes pH 7.5, 5 mM MgCl_2_, containing 1–2 μM MbtI, by the addition of chorismic acid, and monitored using a Perkin Elmer LS3 fluorimeter (Ex. λ = 305 nm, Em. λ = 420 nm). For the inhibition studies, the activity was determined in the presence of the compound at 100 µM (stock solution 20 mM in DMSO) and 50 µM chorismic acid for compounds inhibited by more than 80% of the initial activity. IC_50_ values were determined by measuring the activity at different concentrations of the compound, and using the following equation with GraphPad software:

(1) $$A_{[I]\;}=A_{\left[0\right]}\;\times \;\left(1-\frac{\left[I\right]}{{[I]+\mathrm{IC}}_{50}}\right)$$

where A_[I]_ is the activity at inhibitor concentration [I] and A_[0]_ is the activity in the absence of the inhibitor.

#### 4.2.2. MIC Determination and Siderophore Activity Assay

The minimal inhibitory concentration (MIC_99_) of the most active compounds was determined against *M. bovis BCG* in low-iron Chelated Sauton’s medium, as previously reported [9]. Cells were grown in 7H9 medium, supplemented with 10% OADC, subcultured in chelated Sauton’s medium, and then diluted to an OD_600_ of 0.01 in chelated Sauton’s containing different concentrations of the test compound. After 15 days of incubation at 37 °C, the growth was evaluated by the resazurin reduction assay method (REMA).

Siderophore activity was determined using the Universal CAS liquid assay, as previously reported [29]. Briefly, *M. bovis BCG* was grown in 7H9 medium, subcultured in chelated Sauton’s medium, and then diluted 1:500 in chelated Sauton’s containing different compound concentrations. After 15 days of incubation at 37 °C, 100 μL of culture supernatant was mixed with 100 μL of CAS assay liquid solution in a 96-well plate and incubated 10 min at room temperature; the absorbance was measured at 630 nm. The siderophore units were calculated as follows:

$$\frac{A_{r}-A_{s}}{A_{r}}\times 100$$

where A_r_ is the absorbance at 630 nm of the blank medium and A_s_ is the absorbance of the culture supernatants.

#### 4.2.3. Mycobacterial Growth Conditions

All mycobacterial strains were cultured at 37 °C in Middlebrook 7H9 (Merck, Darmstadt, Germany) medium supplemented with 0.5% BSA, 0.2% glucose, 0.085% NaCl, 0.5% glycerol and Tween-80. Middlebrook 7H10 agar was enriched with 10% OADC (ThermoFisher, Waltham, MA, USA) and 0.5% glycerol. Exponentially growing cultures were started from a single colony, aliquots were enriched with 15% glycerol, stored at −80 °C, and used once to start primary cultures.

Before the infection assay, *M. bovis BCG* and *M. abscessus* ATCC19977 were grown in Middlebrook 7H9 complete media at 37 °C in shaking conditions to mid-log phase (OD_600_ 0.6–0.8). A selective antibiotic was added only for strains carrying a chromosomal integrative vector in the primary culture and removed in the secondary culture used for the final experiment.

#### 4.2.4. *M. bovis BCG* and *M. abscessus* tdTomato Strains Construction

Constitutive reporter strains used in the following study were generated using the integrative vector pTdTomato-L5 (Addgene #140994, Teddington, UK). The chromosomal integration was done via the L5 phage site *attB*. Mycobacteria were transformed with the construct by electroporation (2500 V, 25 μF, 1000 Ω, 2 mm path) and plated on Middlebrook 7H10 agar containing Streptomycin (Sigma) at a concentration of 30 μg/mL. *M. abscessus::tdTomato* and *M. bovis BCG::tdTomato* were named AFA1 and AFB1, respectively.

#### 4.2.5. Cell Culture

All cell lines were incubated at 37 °C in a humidified atmosphere containing 5% CO_2_. All media were supplemented with 10% fetal bovine serum (Thermo Fisher, Waltham, MA, USA), 100 U/mL penicillin (ThermoFisher, Waltham, MA, USA), 0.1 mg/mL streptomycin, and 0.25 μg/mL amphotericin B (ThermoFisher, Waltham, MA, USA). Max Planck Institute cell-2 (MPI-2) (kindly provided by Marina Freudenberg, Max Planck Institute of Immunobiology and Epigenetics, Freiburg, Germany) were cultured in Roswell Park Memorial Institute (RPMI) 1640 medium (ThermoFisher, Waltham, MA, USA) supplemented with 30 ng/mL of murine GM-CSF (Prepotech, London, UK). MPI-2 cells were passaged twice a week at a concentration of 2 × 10^5^ cells/mL. Cells from passage 8 (p8) to 70 (p70) were used. HEK293T (human, female, kidney, were kindly provided by Raffaele De Francesco, INGM, Milan, Italy) were cultured in Dulbecco’s modified Eagle Medium (DMEM). MPI-2 cells stably expressing eGFP were generated by transduction with viral pseudo-particles and selected by FACS. Authentication of the cell lines was performed by microscopic examination and flow cytometry. Furthermore, all cell lines were routinely tested for contamination by mycoplasma.

#### 4.2.6. eGFP viral Pseudoparticles Generation

To generate eGFP viral pseudoparticles, 5 × 10^6^ HEK-293T cells were plated in 15-cm dishes with a complete DMEM medium. The next day, 32 μg of pLenti-CMV-MCS-GFP_SV-puro (Addgene #73582, Teddington, UK), 12.5 μg of pMDLg/pRRE (Addgene #12251, Teddington, UK), 6.25 μg of pRSV-Rev (Addgene #12253, Teddington, UK), and 9 μg of VSV-G (Addegene #12259, Teddington, UK) were co-transfected following a standard procedure based on calcium phosphate transfection. After 12 h from transfection, the medium was changed with 16 mL of complete ISCOVE for each dish. Thirty hours after transfection, the supernatant was collected, clarified by filtration with a 45 μm pore-size filter, and concentrated 400× by centrifugation (2 h at 20,000 rpm) using SW32Ti. Viral pseudoparticles were aliquoted and stored at −80 °C.

#### 4.2.7. Generation of MPI-2 eGFP Cells

MPI-2-eGFP were generated by lentiviral engineering of MPI-2 cell line to stably express eGFP. Lentiviral vectors were produced following a standard procedure based on calcium phosphate cotransfection with a third-generation helper (pMD2.g/VSV.G (Addegene #12259), pRSV-Rev (Addgene #12253), and pMDLg/pRRE (Addegene #12251) and transfer plasmids. The transfer vector pLenti-CMV-MCS-GFP_SV-puro (Addgene #73582) was a kind gift from Raffaele De Francesco (National Institute of Molecular Genetics, Milan, Italy). The expression of eGFP in transduced cells was confirmed by flow cytometry. The expression of eGFP was observed in more than 80% of the cells and found to be stable through cell passages.

#### 4.2.8. RNA Extraction and Real-Time Quantitative PCR

The 5 × 10^5^ MPI-2 and MPI-2-eGFP cells were seeded and cultured in RPMI 1640 media supplemented with 30 ng/mL of murine GM-CSF. After 24 h, MPI-2 and MPI-2-eGFP were collected, and the RNA was extracted according to Monarch^®^ Total RNA Miniprep Kit (NEB) manufacturer’s instruction. cDNA was generated from around 150 ng of RNA using ProtoScript^®^ II First Strand cDNA Synthesis Kit (NEB) in agreement with the manufacturer’s instruction.

qRT-PCR analyses were carried out using Luna^®^ Universal qPCR Master Mix, 0.3 μM primers, and 1 μL of cDNA diluted 1:4. Relative quantification was run on QuantStudio Real-Time PCR System (ThermoFisher, Waltham, MA, USA). Each biological sample was at least measured in duplicate. The following murine gene-specific primers were used: CD68 (Forward: GGCGGTGGAATACAATGTGTCC, Reverse: AGCAGGTCAAGGTGAACAGCTG), CD206 (Forward: AGGACATGCCAGGGTCACCTTT, Reverse: GTTCACCTGGAGTGATGGTTCTC), TNFα (Forward: GGTGCCTATGTCTCAGCCTCTT, Reverse: GCCATAGAACTGATGAGAGGGAG), IL-1β (Forward: GTTCATCTCGGAGCCTGTAGTG, Reverse: TGGACCTTCCAGGATGAGGACA), Gapdh (Forward: CATCACTGCCACCCAGAAGACTG, Reverse: ATGCCAGTGAGCTTCCCGTTCAG).

#### 4.2.9. Infection Assay

**Infection with *M. abscessus***. MPI-2-eGFP cells were seeded in black clear-bottom 96-well plates at a density of 2 × 10^4^ cells per well and incubated at 37 °C with 5% CO_2_ for 24 h before infection. Cells were then infected using an MOI = 1:2 of a *M. abscessus* suspension, and after 1 h cells were extensively washed with PBS and resuspended in 100 μL of RPMI 1640 enriched with 30 ng/mL of murine GM-CSF. The infected MPI-2-eGFP cells were treated with decreasing concentrations of **1f** ranging from 32 to 4 mM. At 48 and 72 h post-infection, the cells were fixed with 50 μL of 4% PFA (Santacruz Biotechnology) at 4 °C for 30 min. The fixed cells were then washed once with PBS and finally resuspended in 100 μL of PBS. eGFP and tdTomato fluorescence was measured by using an Infinite FM200 plate reader (Tecan).

**Infection with *M. bovis BCG***. MPI-2-eGFP cells were seeded in black clear-bottom 96-well plates at a density of 2 × 10^4^ cells *per* well and infected using MOI = 1:5 of a *M. bovis BCG* suspension. After 4 h, the cells were extensively washed with PBS (ThermoFisher, Waltham, MA, USA) and resuspended in 100 μL of RPMI 1640 enriched with 30 ng/mL of murine GM-CSF. The infected MPI-2-eGFP cells were treated with decreasing concentrations of **1f** ranging from 32 to 4 mM. At 2- and 5-days post-infection, cells were then fixed with 50 μL of 4% PFA (Santacruz Biotechnology, Dallas, TX, USA) at 4 °C for 30 min, then washed once with PBS, and finally resuspended in 100 μL of PBS. eGFP and tdTomato fluorescence was measured by using an Infinite FM200 plate reader (Tecan, Männedorf, Switzerland).

For microscopy analysis, cells were imaged with an automated customized high resolution spinning disk confocal microscope based on a Nikon Ti inverted microscope adapted with a CrestOptics X-LightV2/VCS head and Andor cameras (a sCMOS Zyla 4.6 and an EM-CCD DU888) plus two excitation devices with 4 diod lasers (LDI) and 16 LEDs (CoolLED). For the specific assays spectral chroma filters were used for channel separation coupled to LED 490nm and 555nm excitations, using a set of high-NA air objectives including 4x, 10x and 20x (all from Nikon Instruments).

The image analysis and segmentation were performed using Nis-Elements v5.41, creating a binary mask on the eGFP and tdTomato channel. Then, a threshold adjustment via bright-spot detection with a mean diameter of either 20 μm and growing to eGFP intensities, or 2 μm and growing to tdTomato intensity was applied. Next, the two masks were combined to segment the infected cells, and the mean fluorescence intensity of both eGFP and tdTomato was measured.

#### 4.2.10. Statistic

Plots and statistical analysis were generated using Prism 9 (GraphPad). The Brown–Forsythe, Welch and one-way ANOVA values were computed to compare the variation of a single parameter over multiple groups. Two-way ANOVA, followed by correction for multiple comparisons, was performed to analyze the statistical significance between multiple groups. The plots pulled together datasets deriving from at least two independent replicates. Significant *p*-values, sample size and statistical tests are reported in the legends.

### 4.3. Cytotoxicity Assays

#### 4.3.1. MPI-2_eGFP Viability Assay

To test the toxicity of **1f**, MPI-2-eGFPcells were seeded in black, clear-bottom 96-well plates at a density of 2 × 10^4^ cells per well and incubated at 37 °C with 5% CO_2_. After 24 h, the cells were treated with decreasing concentrations of **1f**, starting from 250 mM. At 24 h after treatment, MPI-2-eGFP cells were fixed with 50 μL of 4% PFA (Santacruz Biotechnology) at +4 °C for 30 min, washed once with PBS, and then finally resuspended in 100 μL of PBS. eGFP fluorescence was measured by using an Infinite FM200 plate reader (Tecan, Männedorf, Switzerland).

#### 4.3.2. Blood Collection

**1f** was tested on blood from five healthy volunteers (*n* = 2 males and *n* = 3 females; mean age 27 ± 7 years), who did not take antiplatelet drugs within 10 days before blood withdrawal. Whole blood (WB), drawn with a 19-gauge needle without venous stasis into sodium citrate (0.129M, 1/10 volume/volume)-containing tubes (Vacutainer, Becton Dickinson), discarding the first 4 mL, was incubated with **1f** (100 nM, 500 nM, and 1000 nM) or with solvent (DMSO; final dilution: 0.01%) up to 3 h at room temperature. Untreated (NT) WB of each healthy volunteer was used as a comparison.

#### 4.3.3. Blood Cell Count and Platelet Activation Assessment

PLT, RBC, and WBC counts were assessed at 0, 15, 30, 45, 60, 120 min and 3 h after the addition of **1f** or solvent using Sysmex XE-2100 Automated Hematology Analyzer. Data were reported as count ± SD of each cell population. To evaluate the effect of **1f** on platelet activation, conventional platelet activation markers [P-selectin and activated glycoprotein IIbIIIa (aGPIIbIIIa)], as well as platelet-associated tissue factor (TF) expression, were analyzed by WB flow cytometry as previously described at 0, 30 and 60 min after the addition of the highest concentration of **1f** or solvent. Leukocyte–platelet aggregates were identified as double-positive events for platelet and leukocyte population markers (CD14^pos^/CD41^pos^ or CD66^pos^/CD41^pos^ events). Microvesicle formation, another well-known activation marker, was also assessed by flow cytometry at each time point after the addition of different concentrations of **1f** or solvent. Fluorochrome-conjugated isotype controls were used to quantify the background labeling. A total of 10,000 CD41^pos^ events and 3000 CD14^pos^ events per sample were acquired on a Gallios flow cytometer (Beckman Coulter, Cassina dé Pecchi (Milan), Italy) equipped with four solid-state lasers. Flow-Check Pro Fluorospheres were used daily to monitor cytometer performance. All the data were analyzed with the Kaluza analysis software v1.5 (Beckman Coulter) and reported as percentage ± SD of positive cells or number ± SD of microvesicles.

#### 4.3.4. Statistical Analysis

Results are expressed as mean ± SD and were analyzed by Student’s paired *t*-test or the Mann–Whitney U test, as appropriate. A *p*-value of 0.05 was considered statistically significant.

## 5. Conclusions

Currently, the spread of antibiotic resistance is one of the biggest issues in the management of mycobacterial infections; hence, new treatments are urgently needed to finally eradicate these persistent and recalcitrant diseases. Interestingly, iron metabolism appears to be a crucial asset for both the pathogen and the host. Indeed, the host might simultaneously use iron to promote macrophage polarization towards a pro-inflammatory phenotype and induce iron depletion in the bacteria to induce its death. Nonetheless, the bacteria can counteract such mechanisms by synthesizing siderophores to sequester iron and, consequently, limit host iron resources, thus promoting an anti-inflammatory phenotype and a more permissive environment. Hence, targeting such biosynthetic pathways might represent a promising winning approach to promote mycobacterial clearance.

In this work, a SAR study on our series of MbtI inhibitors led to the identification of new candidates endowed with potent activity against the enzyme and encouraging bactericidal properties. For the new products, we described the design, synthesis, analytical characterization, and biological activity. The best candidate **1f** showed potent MbtI inhibitory effect (IC_50_ ≈ 12 μM), comparable to that of the previous lead **I** but exhibited an enhanced antimycobacterial action (MIC_99_ = 63 μM), thus becoming one of the few potent MbtI inhibitors endowed with a promising antitubercular activity. Moreover, cytotoxicity assays demonstrated that **1f** does not affect blood cell count and platelet activation.

Currently, antimycobacterial drug development suffers from the lack of an in vitro cellular model that could closely mimic the physiologic mycobacteria–host interaction. To overcome such a limitation and to test the antimicrobial activity of **1f** in a physiological setting, we performed our study using the recently characterized MPI-2 cells. We established an in vitro screening assay based on a fluorescence measurement, able to assess both host and pathogen viability. The assay proposed herein can be readily scaled-up, does not require extensive training, is cost-effective and practical, and has the important potential to accelerate the discovery of new drugs against TB.

Overall, these findings could help to fill the gap in the understanding of the mechanism of action of these enzymatic inhibitors, especially considering that the pathogen–macrophage interaction is critical to shedding light on the pathways connecting both bacterial virulence and host immunity.

## Figures and Tables

**Table 1:** In vitro activity of compounds **1a–j**.

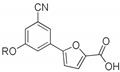				
**Entry**	**R**	**% RA ^†^**	**IC_50_ (μM) ^‡^**	**MIC_99_ (μM) ^§^**
**1a**	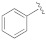	14.0 ± 4.1	23.1 ± 2.6	125
**1b**	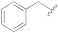	1.2 ± 0.4	15.6 ± 1.2	250
**1c**	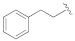	0.6 ± 0.5	3.0 ± 2.9	250
**1d**	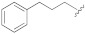	25.6 ± 5.8	−	−
**1e**	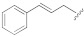	11.5 ± 1.7	13.5 ± 1.0	125
**1f**	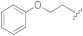	5.5 ± 2.4	12.1 ± 2.0	63
**1g**	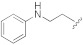	9.3 ± 2.2	18.0 ± 0.9	250
**1h**	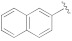	12.7 ± 2.0	16.3 ± 1.2	125
**1i**	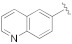	13.9 ± 3.1	16.6 ± 1.0	125
**1j**	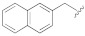	19.8 ± 6.5	34.8 ± 2.5	250

^†^ Percentage of residual enzymatic activity at 100 μM. ^‡^ IC_50_ values were calculated for compounds showing a %RA < 20. ^§^ MIC_99_ were determined against *M. bovis BCG*. Values were calculated for compounds showing a %RA < 20.

## Data Availability

Data is contained within the article and Supplementary Material.

## Supplementary Materials

The supporting information can be downloaded at: https://www.mdpi.com/article/10.3390/ph15080992/s1.
