# Supplementary material for: Synthesis and Assessment of the In Vitro and Ex Vivo Activity of Salicylate Synthase (Mbti) Inhibitors as New Candidates for the Treatment of Mycobacterial Infections
Source: Pharmaceuticals (Basel). 2022 Aug 11;15(8):992. doi: 10.3390/ph15080992 (PMC9413995; doi:10.3390/ph15080992)
Supplement: Supplementary file 1 [file pharmaceuticals-15-00992-s001.zip › pharmaceuticals-1840585-supplementary.pdf]

Supplementary Material

Synthesis and assessment of the *in vitro* and *ex vivo* activity of salicylate synthase (MbtI) inhibitors as new candidates for the treatment of mycobacterial infections

Matteo Mori, Anna Griego, Giovanni Stelitano, Laurent R. Chiarelli, Giulia Cazzaniga, Arianna Gelain, Elena Pini, Marina Camera, Paola Canzano, Andrea Fumagalli, Edoardo Scarpa, Chiara Cordiglieri, Loris Rizzello, Stefania Villa and Fiorella Meneghetti

Table of contents

1. Synthetic procedures	S2
2. Analytical data	S5
3. Calculated LogP (cLogP) values	S46
4. Siderophore production assay	S47
5. Infection assays	S48

1. Synthetic procedures

Methyl 5-(3-cyano-5-hydroxyphenyl)furan-2-carboxylate (3 SB5D)

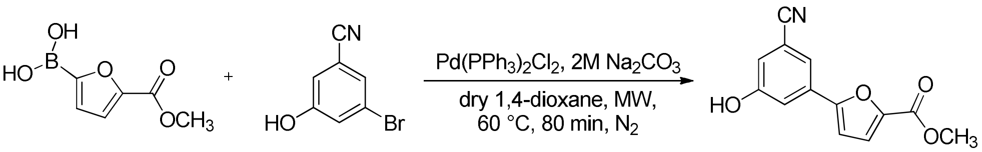

3-Bromo-5-hydroxybenzonitrile (198 mg, 1.0 mmol), (5-(methoxycarbonyl)furan-2-yl)boronic acid (222 mg, 1.3 mmol), and bis(triphenylphosphine)palladium dichloride (35 mg, 0.05 mmol) were dissolved in 1,4-dioxane (5.0 mL) under a nitrogen atmosphere. A 2M Na_2CO_3 solution (1.0 mL, 2.0 mmol) was added, and the resulting mixture was stirred in a microwave synthesizer at 60 °C for 1 h. The reaction mixture was filtered on a pad of celite, diluted with water, and extracted with EtOAc (3 x 10 mL). The organic layers were washed with brine, dried over anhydrous Na_2SO_4, filtered, and concentrated under reduced pressure. The crude product was purified by flash column chromatography on silica gel (cyclohexane–EtOAc 7:3) to afford the desired product. Yield: 97%. Aspect: greenish solid. Mp: 236 °C (dec.). TLC (cyclohexane–EtOAc 8:2): R_f = 0.16. ^{1}H NMR (300 MHz, DMSO-*d$_6$*) δ (ppm): 10.51 (br s exch. D_2O, 1H, OH), 7.74 (t, *J* = 1.5 Hz, 1H, H_{Ar}), 7.50 (dd, *J* = 2.4, 1.5 Hz, 1H, H_{Ar}), 7.43 (d, *J* = 3.7 Hz, 1H, H_{Ar}), 7.31 (d, *J* = 3.7 Hz, 1H, H_{Ar}), 7.15 (dd, *J* = 2.4, 1.5 Hz, 1H, H_{Ar}), 3.83 (s, 3H, OCH_3).

3-Bromo-5-phenoxybenzonitrile (4a AC11A)

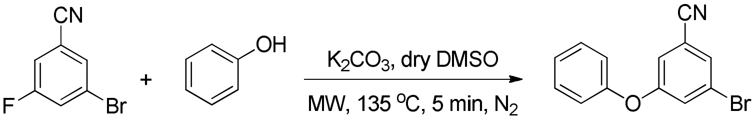

General Procedure SA. 3-Bromo-5-fluorobenzonitrile (100 mg, 0.5 mmol), the appropriate alcohol (94 mg, 0.5 mmol), and K_2CO_3 (138 mg, 1.0 mmol) were dissolved in dry DMSO (1.2 mL) under a nitrogen atmosphere. The resulting mixture was stirred in a microwave synthesizer for 5 min at 135 °C. After completion, the reaction was diluted with water and extracted with diethyl ether (3 x 3mL). The organic layer was washed with cold water, dried over anhydrous Na_2SO_4, filtered, and concentrated *in vacuo*. The product was used in the next step without further purification. Starting compound: phenol. Yield: 57%. Aspect: white solid. Mp: 90 °C. TLC (cyclohexane–EtOAc 8:2): R_f = 0.72**.** ^{1}H-NMR (300 MHz, $CDCl_3$) δ (ppm): 7.51 – 7.39 (m, 3H, H_{Ar}), 7.35 (t, *J* = 2.0 Hz, 1H, H_{Ar}), 7.30 – 7.21 (m, partially overlapped with solvent peak, 1H, H_{Ar}), 7.14 (dd, *J* = 2.4, 1.3 Hz, 1H, H_{Ar}), 7.09 – 7.00 (m, 2H, H_{Ar}).

(*E*)-(3-Bromoprop-1-en-1-yl)benzene (4e SB5E)

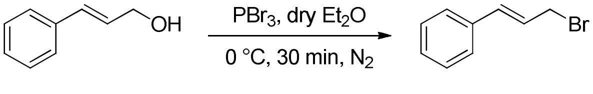

To a solution of cinnamyl alcohol (134 mg, 1.0 mmol) in dry Et_2O, PBr_3 (54 mg, 0.2 mmol) was added at 0 °C. The reaction mixture was stirred at 0 °C for 30 min under a nitrogen atmosphere. After quenching with a saturated solution of $NaHCO_3$ (10 mL), the mixture was extracted with Et_2O (3 x 10 mL). The organic layers were dried over anhydrous Na_2SO_4, filtered, and concentrated *in vacuo* to afford the desired product. Yield: 85%. Aspect: yellow solid. Mp: 35 °C. TLC (cyclohexane–EtOAc 8:2): R_f = 0.84. ^{1}H NMR (300

MHz, $CDCl_3$) δ (ppm): 7.43 – 7.22 (m, partially overlapped with solvent peak, 5H, H_{Ar}), 6.65 (d, *J* = 15.6 Hz, 1H, CH), 6.40 (dt, *J* = 15.6, 7.8 Hz, 1H, CH), 4.16 (dd, *J* = 7.8, 0.9 Hz, 2H, CH_2).

3-Bromo-5-(2-phenoxyethoxy)benzonitrile (4f SB3A)

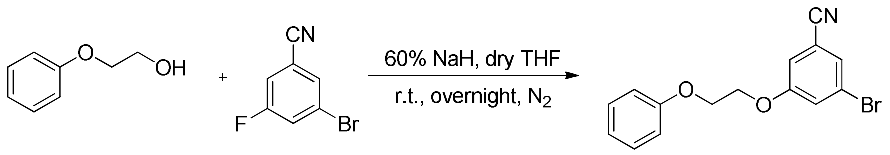

General Procedure SB. The suitable primary alcohol (138 mg, 1.0 mmol) was dissolved in dry THF (3.5 mL) under a nitrogen atmosphere, and NaH (60% suspension in paraffin oil, 60 mg, 1.5 mmol) was added at 0 °C. The mixture was stirred at 0 °C for 30 min. Then, 3-bromo-5-fluorobenzonitrile (300 mg, 1.5 mmol) was added, and the stirring was continued at r. t. overnight. After quenching with a saturated aqueous solution of NH_4Cl (1.79 mL), the mixture was extracted with EtOAc (3 x 10 mL). The organic phases were washed with brine, dried over anhydrous Na_2SO_4, filtered, and concentrated under reduced pressure. The crude product was purified to afford the desired products. Starting compound: 2-phenoxyethanol. Purification: flash column chromatography (cyclohexane–EtOAc 9:1). Yield: 70%. Aspect: white solid. Mp: 80 °C. TLC (cyclohexane–EtOAc 8:2): R_f = 0.58. ^{1}H NMR (300 MHz, $CDCl_3$) δ (ppm): 7.45 – 7.24 (m, partially overlapped with solvent peak, 4H, H_{Ar}), 7.16 (dd, *J* = 2.4, 1.3 Hz, 1H, H_{Ar}), 7.04 – 6.79 (m, 3H, H_{Ar}), 4.34 (s, 4H, CH_2).

3-Bromo-5-(2-(phenylamino)ethoxy)benzonitrile (4g DA1B)

The compound was obtained according to General Procedure **SB**. Starting compound: 2-(phenylamino)-ethanol (**6**). Purification: flash column chromatography (cyclohexane–EtOAc 75:25), recrystallization from DCM/hexane. Yield: 45%. Aspect: white solid. Mp: 75 °C. TLC (cyclohexane–EtOAc 7:3): R_f = 0.74. ^{1}H NMR (300 MHz, $CDCl_3$) δ (ppm): 7.38 (t, *J* = 1.4 Hz, 1H, H_{Ar}), 7.30 (dd, *J* = 2.5, 1.4 Hz, 1H, H_{Ar}), 7.28 – 7.16 (m, partially overlapped with solvent peak, 2H, H_{Ar}), 7.11 (dd, *J* = 2.5, 1.4 Hz, 1H, H_{Ar}), 6.77 (td, *J* = 7.3, 1.2 Hz, 1H, H_{Ar}), 6.72 – 6.64 (m, 2H, H_{Ar}), 4.16 (t, *J* = 5.2 Hz, 2H, CH_2), 4.01 (br s exch. D_2O, 1H, NH), 3.58 (br q, *J* = 5.2 Hz, 2H, CH_2).

3-Bromo-5-(naphthalen-2-yloxy)benzonitrile (4h SB2A).

The compound was obtained according to General Procedure **SA**. Starting compound: naphthalen-2-ol. Purification: flash column chromatography (cyclohexane–EtOAc 95:5). Yield: 65%. Aspect: light-yellow solid. Mp: 66 °C. TLC (cyclohexane–EtOAc 8:2): R_f = 0.86. ^{1}H NMR (300 MHz, $CDCl_3$) δ (ppm): 7.96 – 7.85 (m, 2H, H_{Ar}), 7.83 – 7.76 (m, 1H, H_{Ar}), 7.55 – 7.47 (m, 3H, H_{Ar}), 7.44 (d, *J* = 2.4 Hz, 1H, H_{Ar}), 7.41 – 7.37 (m, 1H, H_{Ar}), 7.25 – 7.15 (m, 2H, H_{Ar}).

3-Bromo-5-(quinolin-7-yloxy)benzonitrile (4i SB7A)

The compound was obtained according to General Procedure **SA**. Starting compound: quinolin-7-ol. Purification: flash column chromatography (cyclohexane–EtOAc 7:3). Yield: 40%. Aspect: white solid. Mp: 165 °C. TLC (cyclohexane–EtOAc 8:2): R_f = 0.20. ^{1}H NMR (300 MHz, $CDCl_3$) δ (ppm): 8.92 (d, *J* = 4.0 Hz, 1H, H_{Ar}), 8.22 (d, *J* = 8.2 Hz, 1H, H_{Ar}), 7.91 (d, *J* = 8.9 Hz, 1H, H_{Ar}), 7.65 (t, *J* = 1.7 Hz, 1H, H_{Ar}), 7.57 (d, *J* = 1.5 Hz, 1H, H_{Ar}), 7.52 – 7.39 (m, 2H, H_{Ar}), 7.38 – 7.23 (m, partially overlapped with solvent peak, 2H, H_{Ar}).

2-(Bromomethyl)naphthalene (4j SB10A)

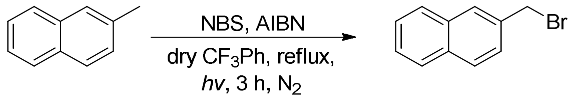

N-Bromosuccinimide (NBS, 138 mg, 0.770 mmol) and azobisisobutyronitrile (AIBN, 13 mg, 0.077 mmol) were suspended in dry trifluorotoluene (2.3 mL) under a nitrogen atmosphere. Then, a solution of 2-methylnaphthalene (100 mg, 0.700 mmol) in dry trifluorotoluene (1.8 mL) was added. The reaction mixture was irradiated with a tungsten lamp and heated at reflux for 3 h. Then, the mixture was cooled at r.t. and filtered *in vacuo*. The solvent was evaporated under reduced pressure, and the solid was taken up in DCM. The organic phase was washed twice with a saturated aqueous solution of $Na_2S_2O_3$, dried over anhydrous Na_2SO_4, and concentrated under reduced pressure. The crude product was purified *via* flash column chromatography on silica gel (cyclohexane–DCM 95:5) to afford the desired product. Yield: 55%. Aspect: white solid. Mp: 52 °C. TLC (cyclohexane–DCM 9:1): R_f = 0.50. ^{1}H NMR (300 MHz, $CDCl_3$) δ (ppm): 7.93 – 7.72 (m, 4H, H_{Ar}), 7.60 – 7.41 (m, 3H, H_{Ar}), 4.67 (s, 2H, CH_2).

3-Bromo-5-hydroxybenzonitrile (5)

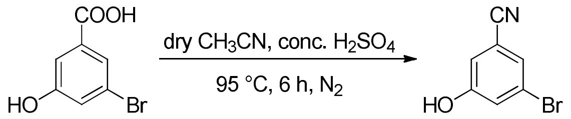

Concentrated H_2SO_4 (1.23 mL, 2.25 mmol) was added dropwise to a well-stirred suspension of 3-bromo-5-hydroxybenzoic acid (1.15 mmol) in CH_3CN (5.74 mL, 109 mmol) at room temperature; the mixture was stirred at 95 °C for 6 h under N_2 atmosphere. Then, the reaction was diluted with H_2O (17 mL) and extracted with EtOAc (3 x 17 mL). The organic layers were dried over anhydrous Na_2SO_4, filtered, and concentrated under reduced pressure. The crude product was purified *via* flash column chromatography on silica gel (cyclohexane–EtOAc 7:3) to afford the desired product as a white solid. Yield: 30%. Mp: 160 °C. TLC (cyclohexane–EtOAc 8:2): R_f = 0.36. ^{1}H-NMR (300 MHz, $CDCl_3$) δ (ppm): 7.36 – 7.33 (m, 1H, H_{Ar}), 7.26 – 7.24 (m, partially overlapped with solvent peak, 1H, H_{Ar}), 7.06 (dd, *J* = 2.4, 1.3 Hz, 1H, H_{Ar}).

2-(Phenylamino)-ethanol (6 DA1A)

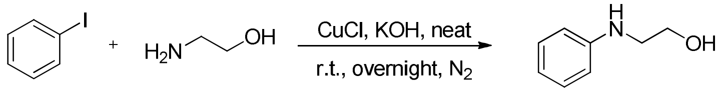

Iodobenzene (1000 mg, 4.90 mmol, 0.55 mL) and ethanolamine (898 mg, 14.7 mmol, 0.89 mL) were added to CuCl (48 mg, 0.49 mmol) and KOH (550 mg, 9.80 mmol) under a nitrogen atmosphere. The reaction mixture was stirred at r.t. overnight. Then, the solution was diluted with water (9.80 mL) and extracted with EtOAc (3 x 9.80 mL). The organic layer was dried over Na_2SO_4, filtered, and concentrated under reduced pressure. The crude product was purified *via* flash column chromatography on silica gel (cyclohexane–EtOAc 5:5) to afford the desired product. Yield: 92%. Aspect: yellow oil. TLC (cyclohexane–EtOAc 5:5): R_f = 0.38. ^{1}H NMR (300 MHz, $CDCl_3$) δ (ppm): 7.28 – 7.12 (m, partially overlapped with solvent peak, 2H, H_{Ar}), 6.81 – 6.60 (m, 3H, H_{Ar}), 3.84 (t, *J* = 5.2 Hz, 2H, CH_2), 3.32 (t, *J* = 5.2 Hz, 2H, CH_2).

2. Analytical data

5-(3-Cyano-5-phenoxyphenyl)furan-2-carboxylic acid (1a)

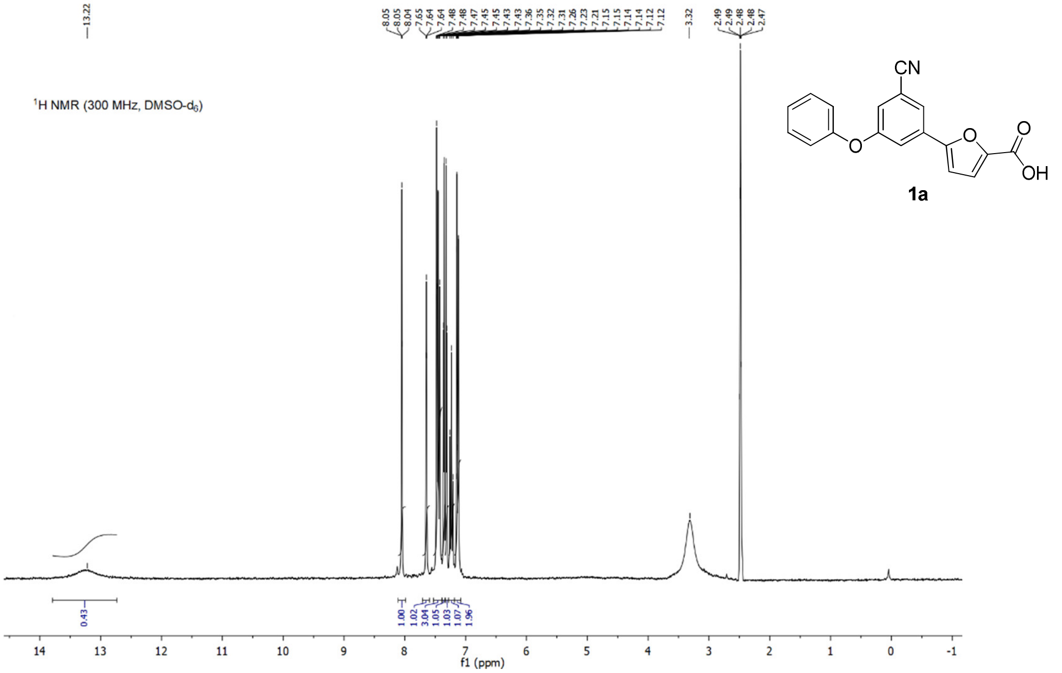

Figure S1. ^{1}H NMR spectrum of **1a**.

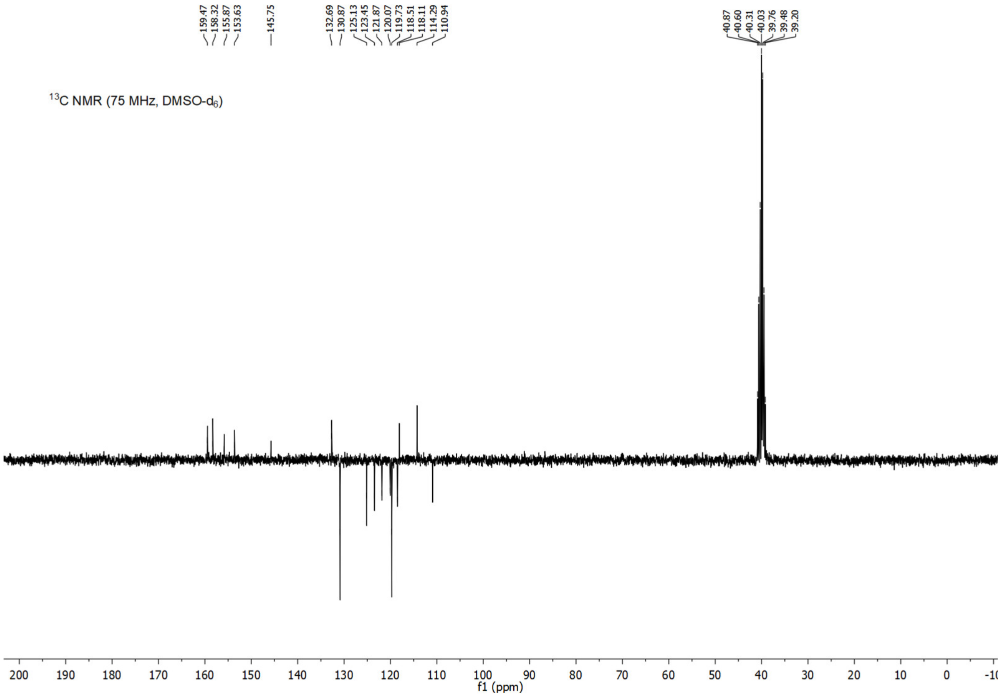

Figure S2. ^{13}C NMR spectrum of **1a**.

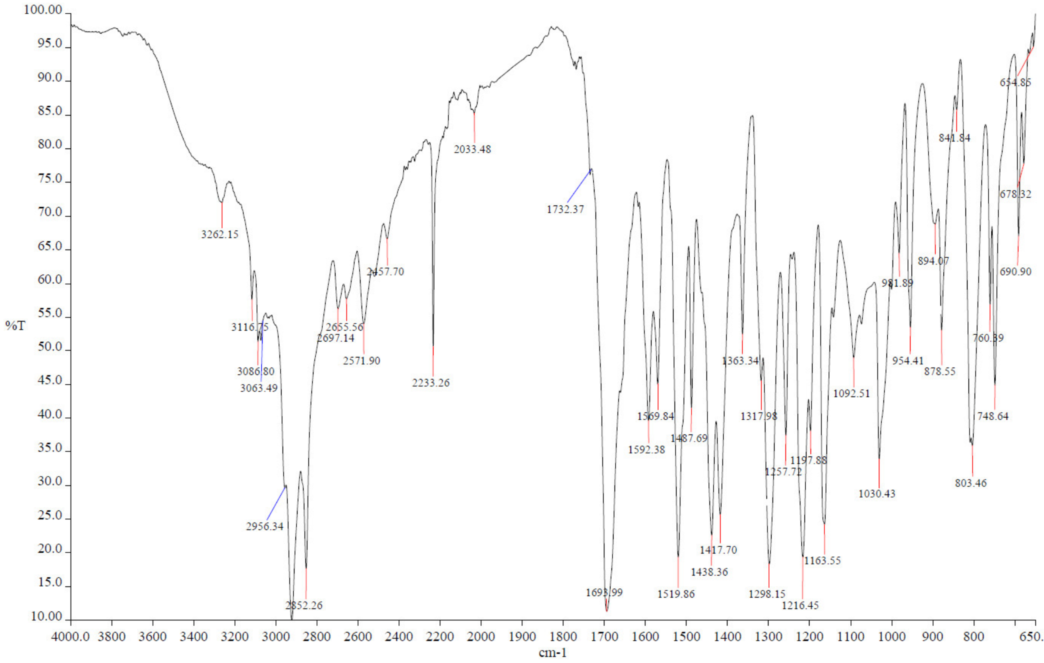

Figure S3. FT-IR spectrum of **1a**.

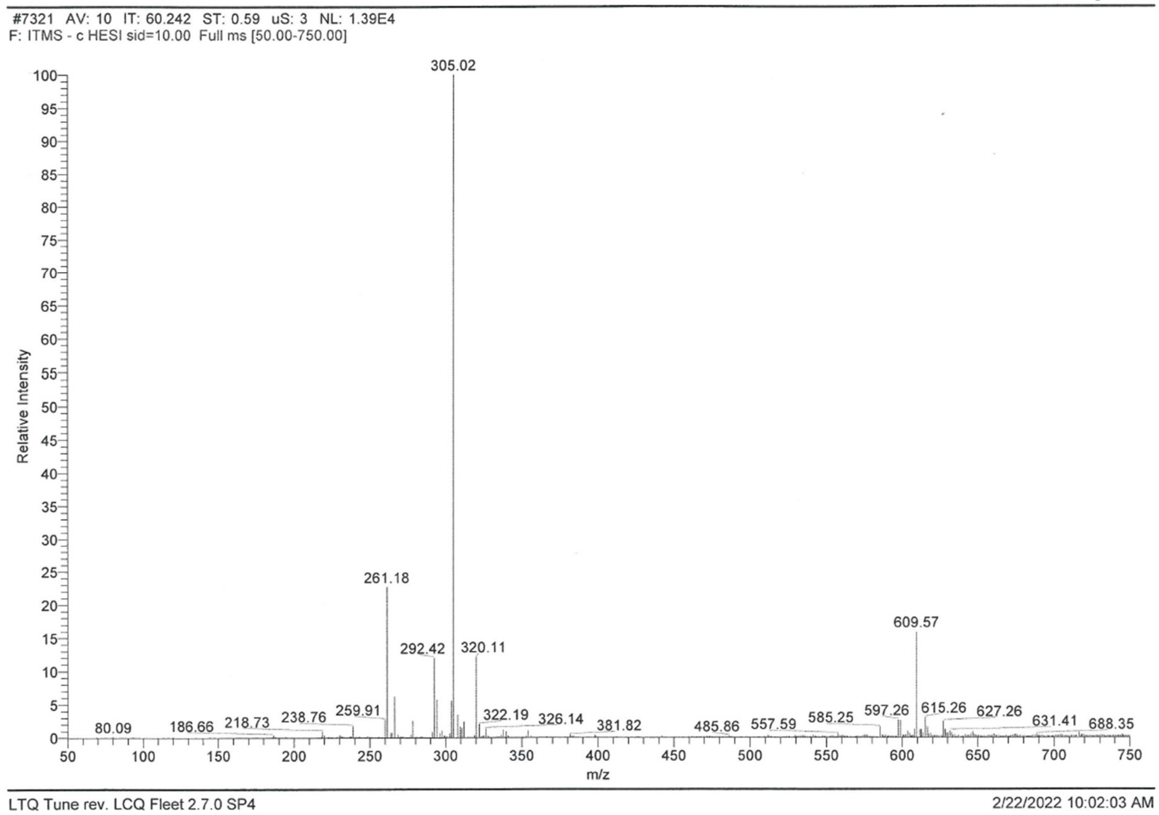

Figure S4. ESI-MS spectrum of **1a**.

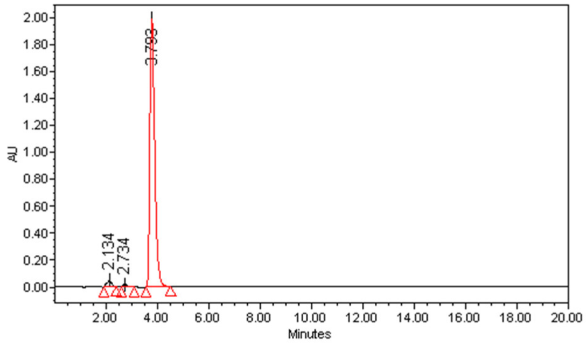

1a	
TR (min)	**A (%)**
2.134	2.43
2.734	0.86
3.793	96.71

Figure S5. HPLC chromatogram of **1a**.

5-(3-(Benzyloxy)-5-cyanophenyl)furan-2-carboxylic acid (1b)

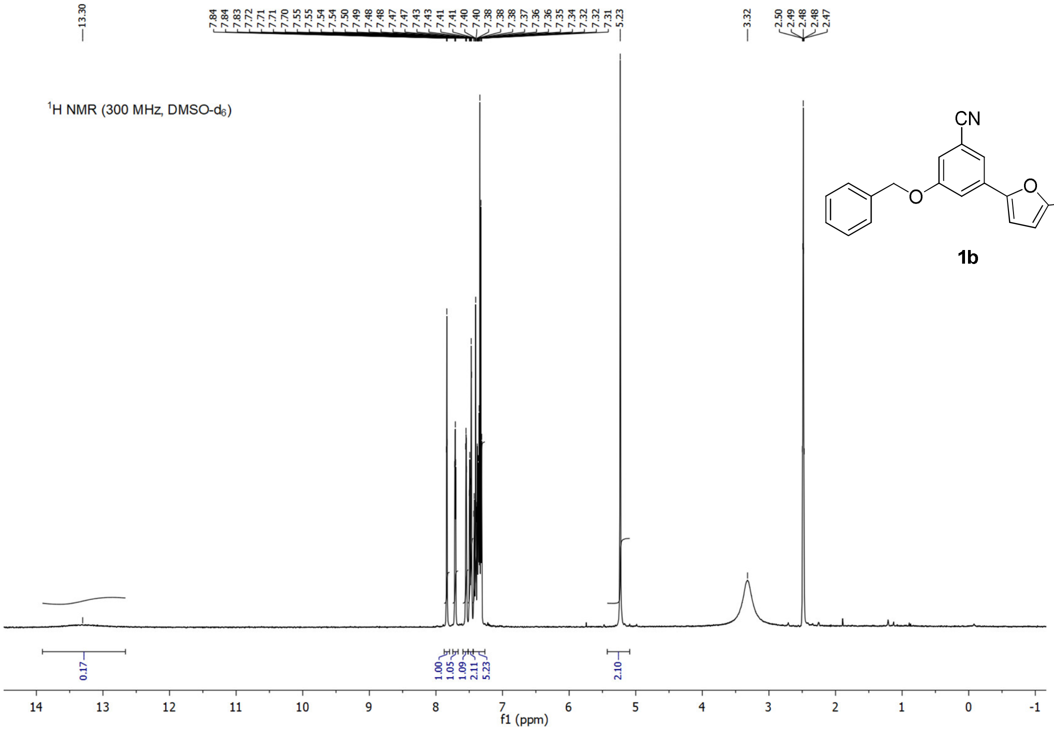

Figure S6. ^{1}H NMR spectrum of **1b**.

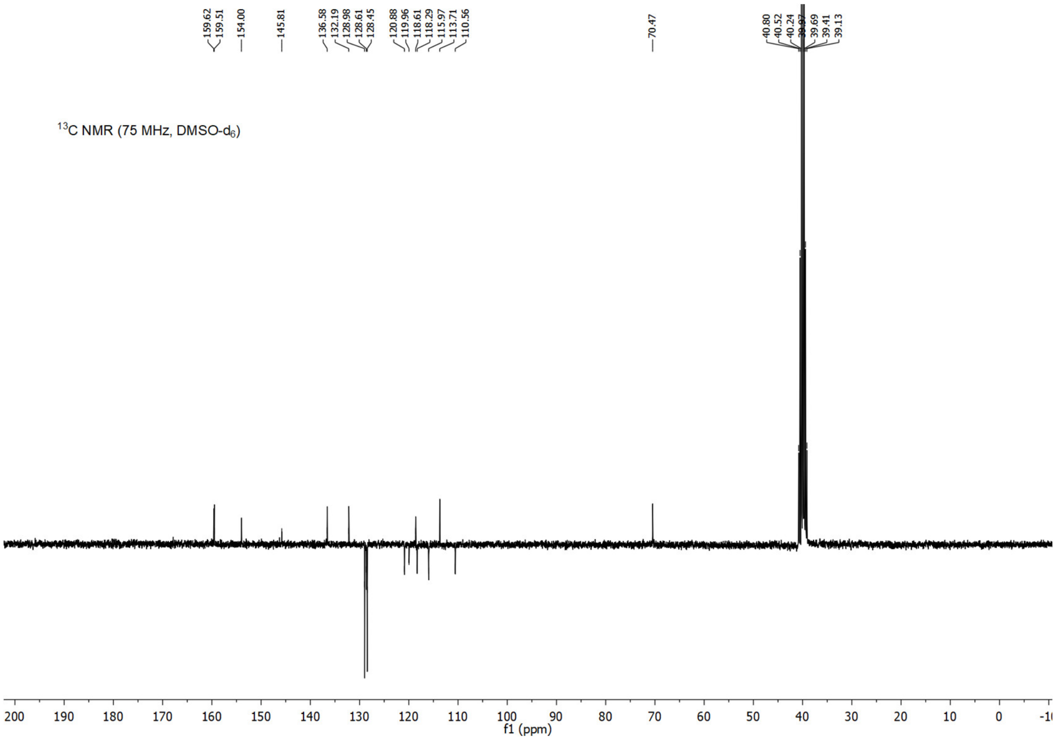

Figure S7. ^{13}C NMR spectrum of **1b**.

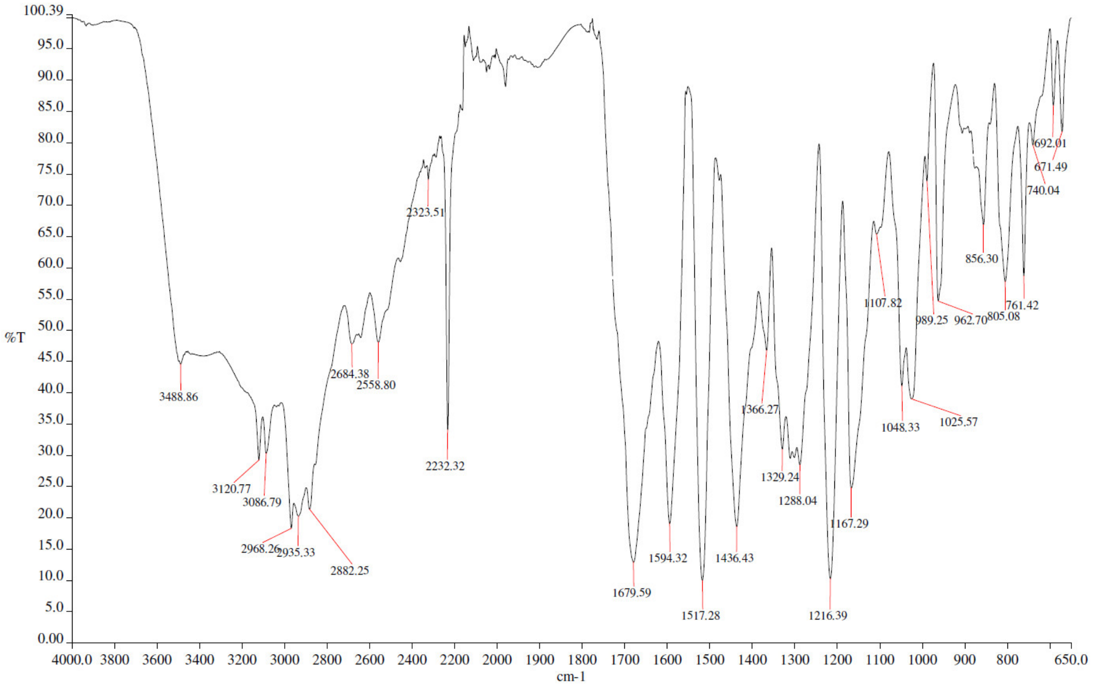

Figure S8. FT-IR spectrum of **1b**.

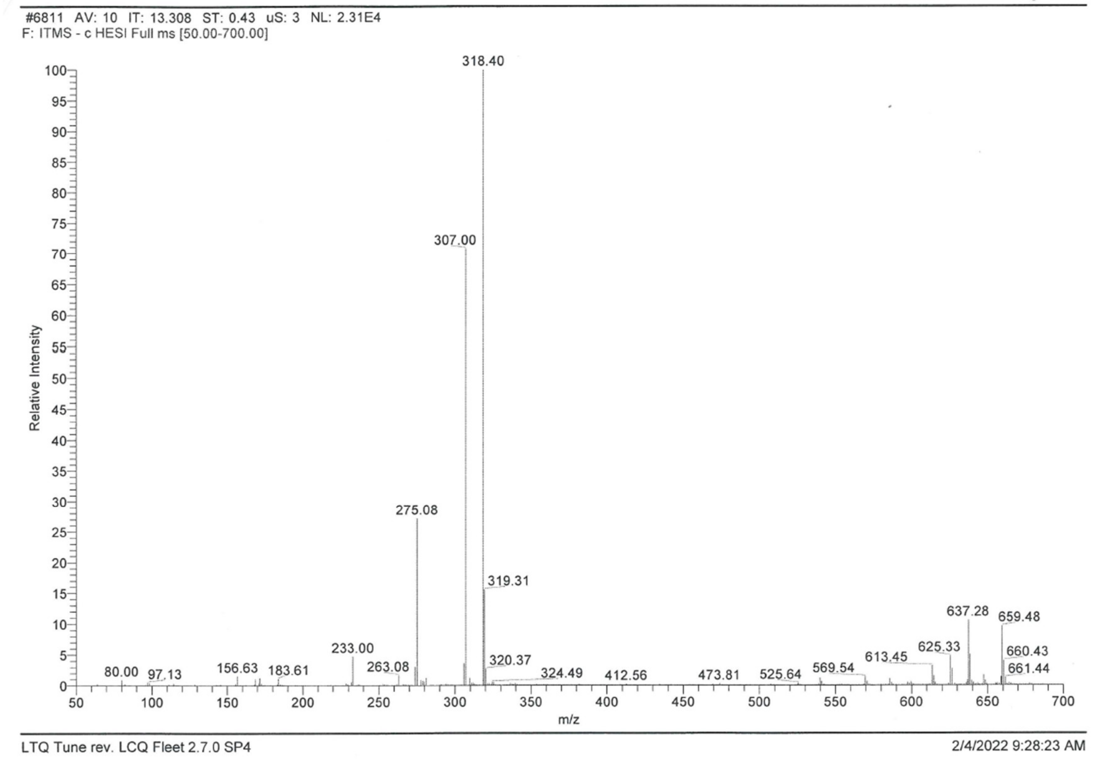

Figure S9. ESI-MS spectrum of **1b**.

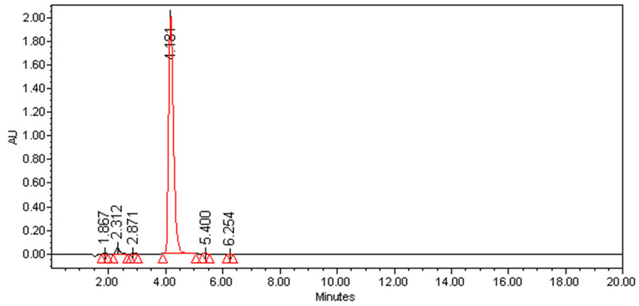

1b	
TR (min)	***A (%)***
1.867	0.13
2.312	1.90
2.871	0.20
4.181	97.69
5.400	0.04
6.254	0.03

Figure S10. HPLC chromatogram of **1b**.

5-(3-Cyano-5-phenethoxyphenyl)furan-2-carboxylic acid (1c)

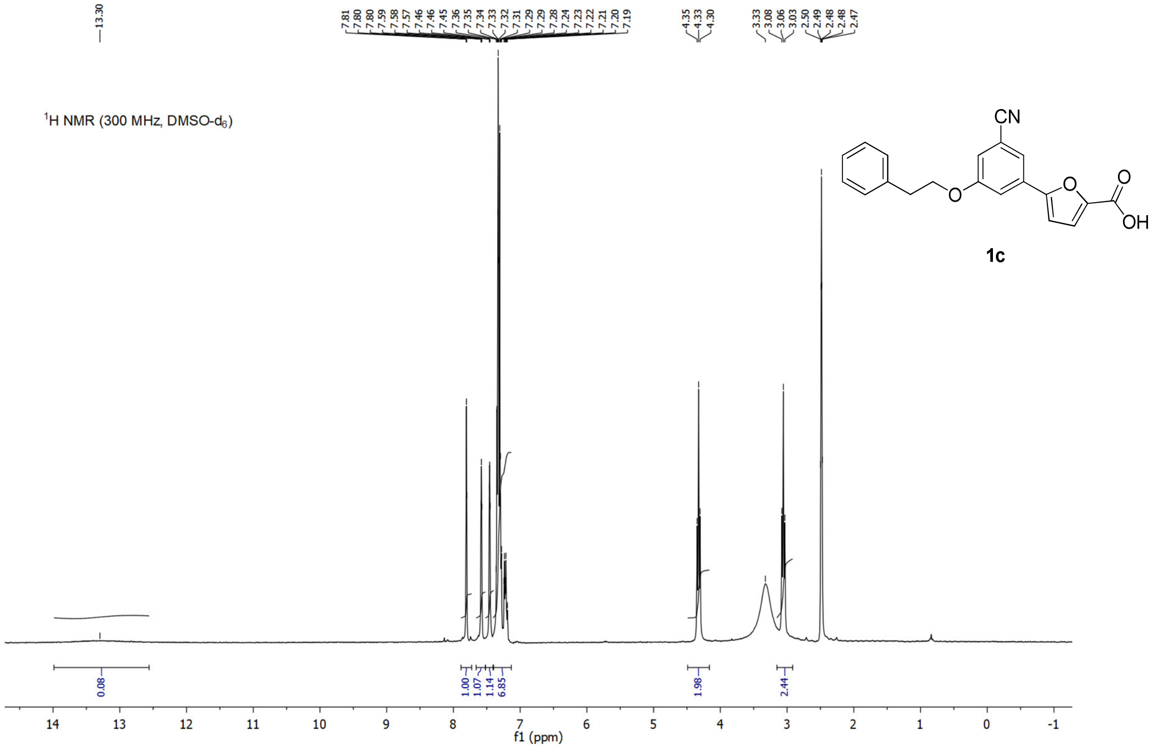

Figure S11. ^{1}H NMR spectrum of **1c**.

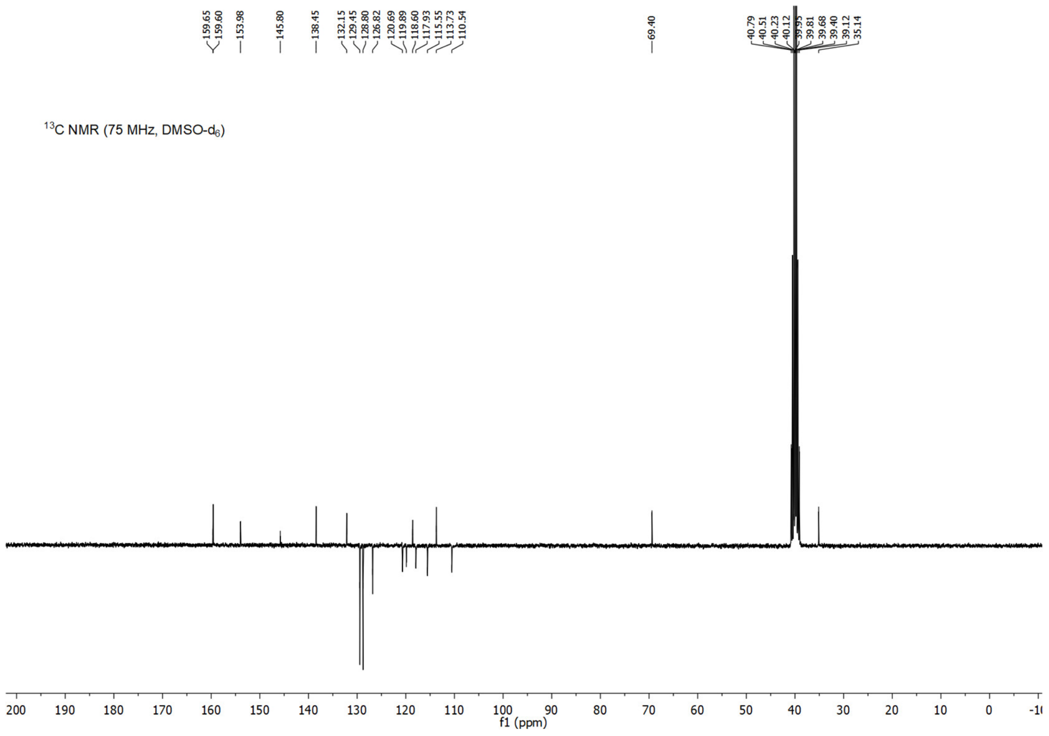

Figure S12. ^{13}C NMR spectrum of **1c**.

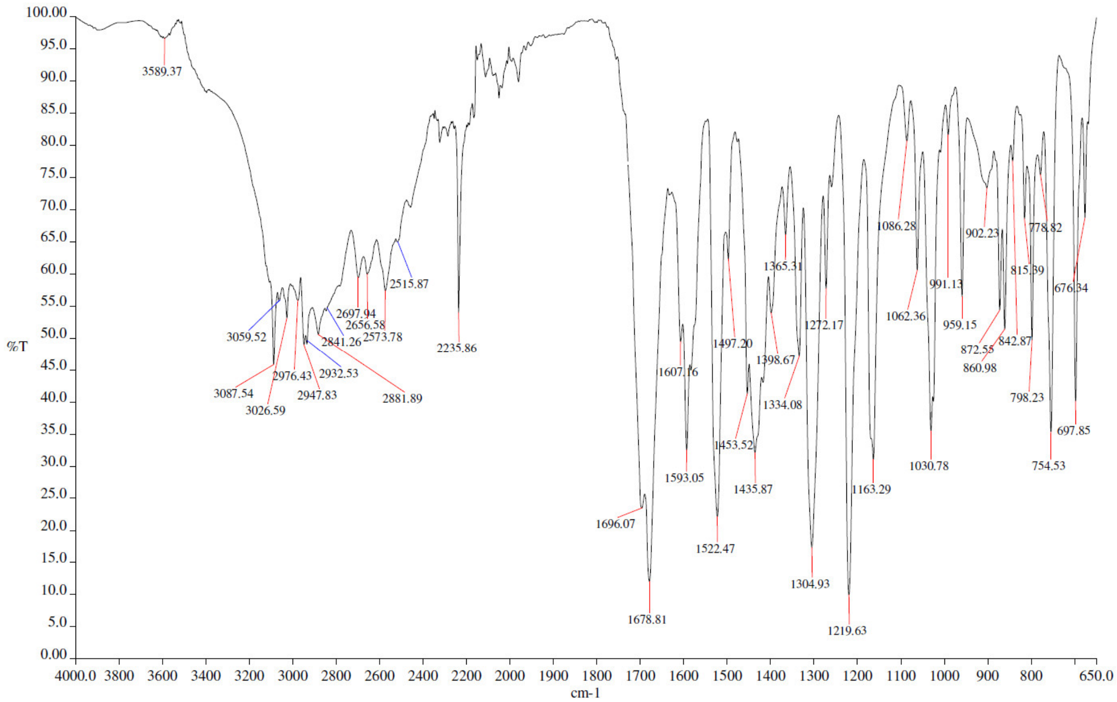

Figure S13. FT-IR spectrum of **1c**.

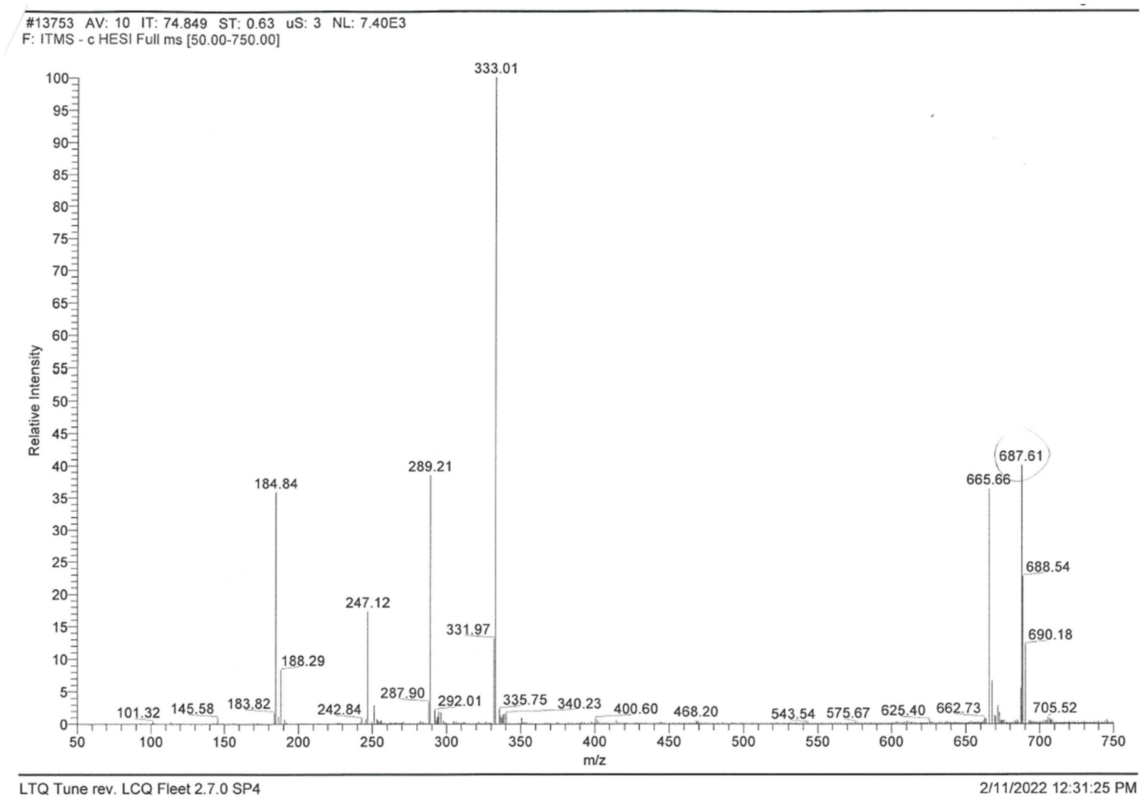

Figure S14. ESI-MS spectrum of **1c**.

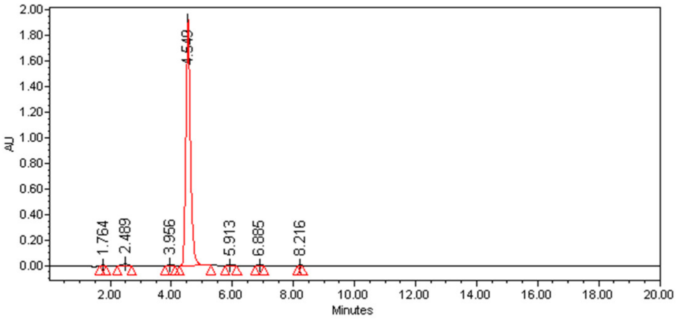

1c	
TR (min)	***A (%)***
1.764	0.07
2.489	0.80
3.956	0.43
4.549	98.45
5.913	0.11
6.885	0.11
8.216	0.03

Figure S15. HPLC chromatogram of **1c**.

5-(3-Cyano-5-(3-phenylpropoxy)phenyl)furan-2-carboxylic acid (1d)

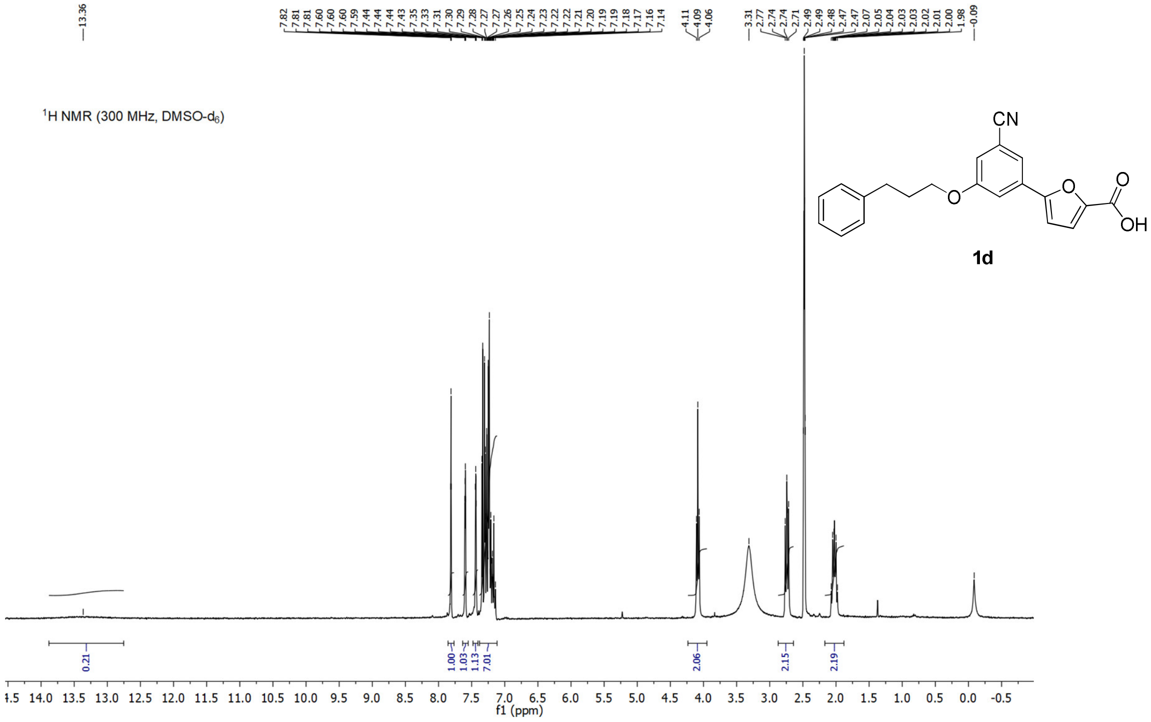

Figure S16. ^{1}H NMR spectrum of **1d**.

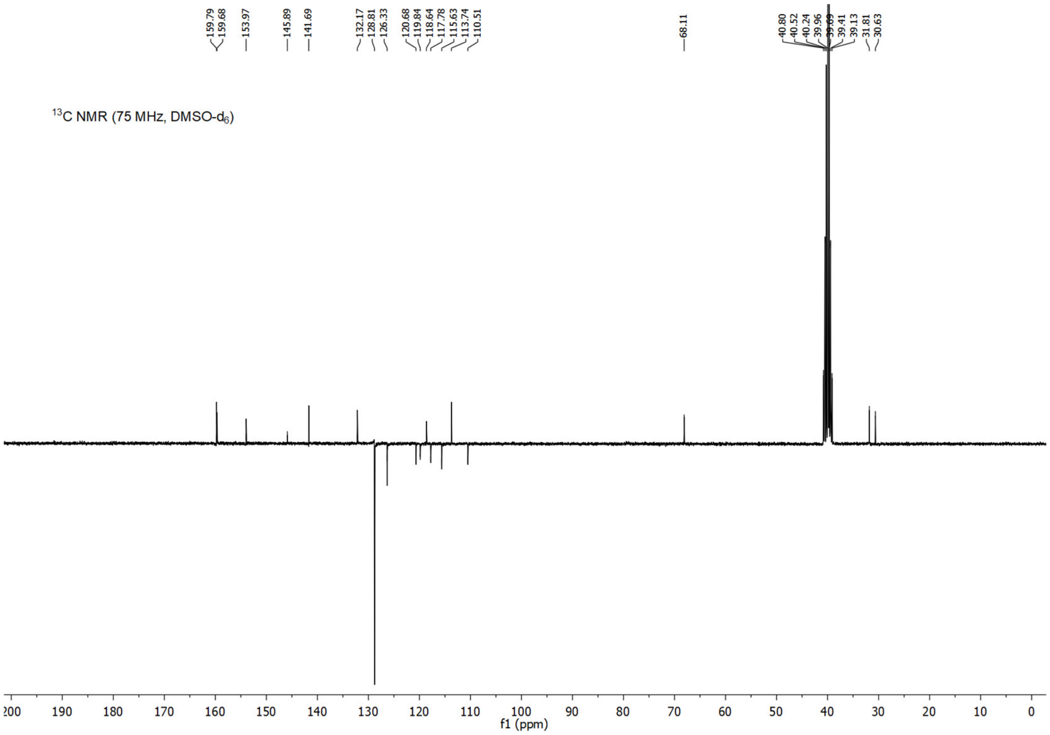

Figure S17. ^{13}C NMR spectrum of **1d**.

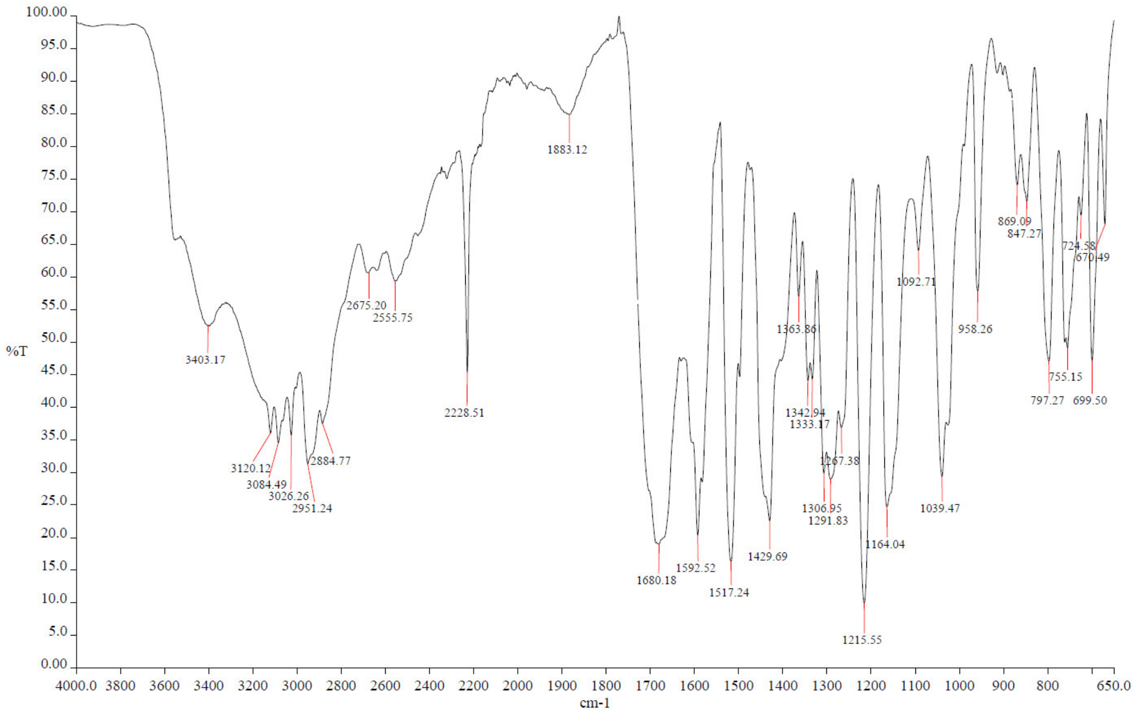

Figure S18. FT-IR spectrum of **1d**.

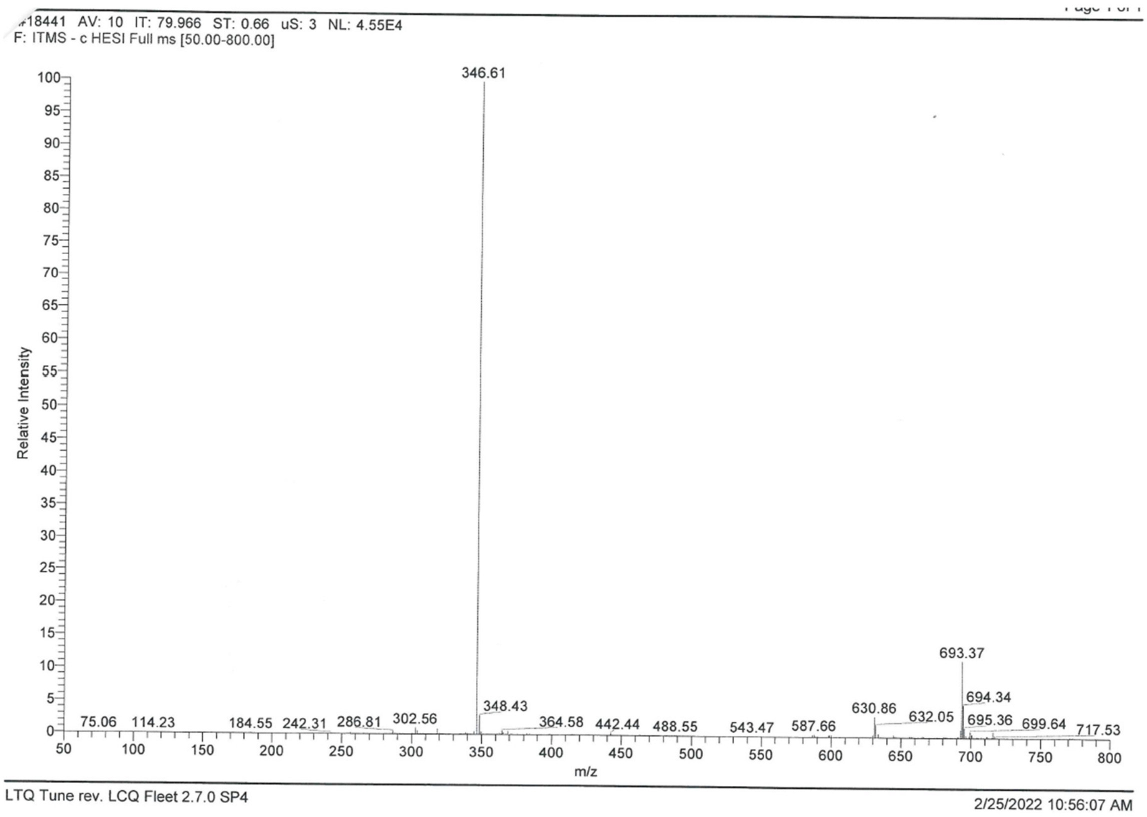

Figure S19. ESI-MS spectrum of **1d**.

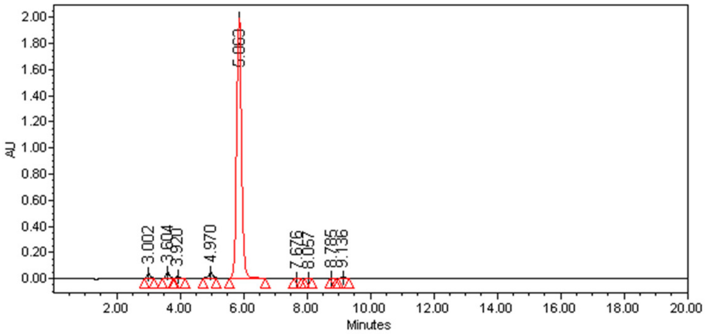

1d	
TR (min)	***A (%)***
3.002	1.06
3.604	1.51
3.920	0.36
4.970	1.72
5.863	95.05
7.676	0.03
8.057	0.03
8.785	0.02
9.136	0.21

Figure S20. HPLC chromatogram of **1d**.

(*E*)-5-(3-(Cinnamyloxy)-5-cyanophenyl)furan-2-carboxylic acid (1e)

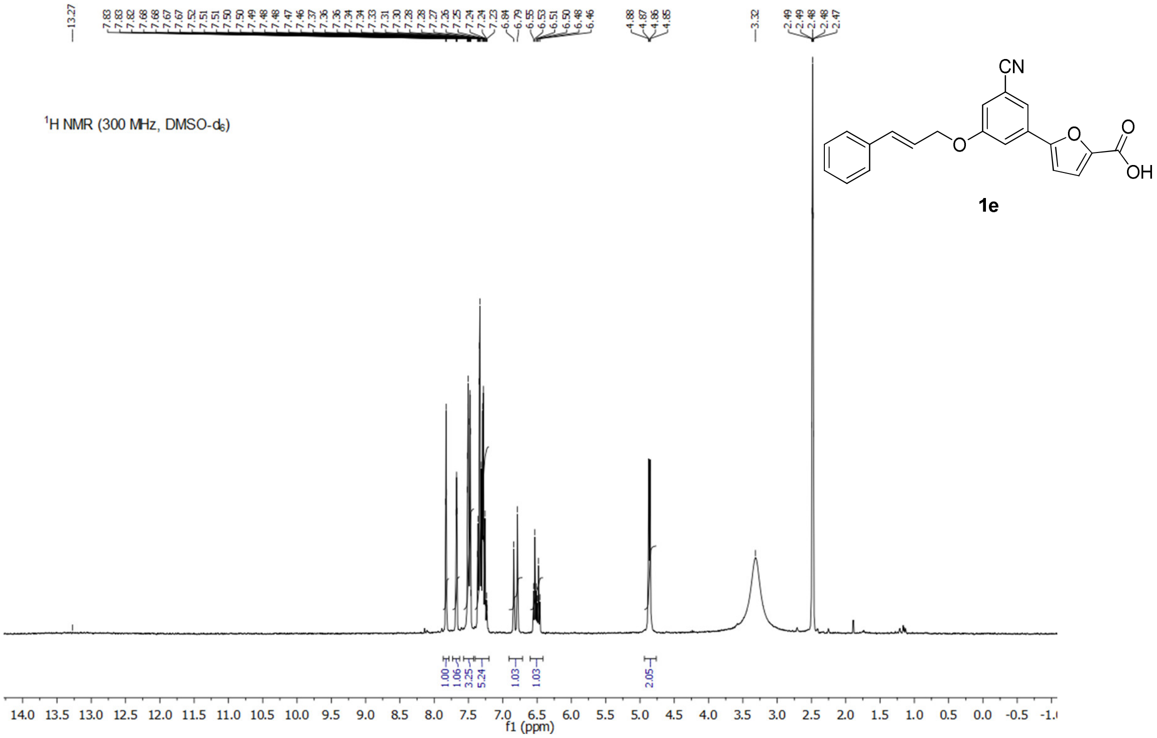

Figure S21. ^{1}H NMR spectrum of **1e**.

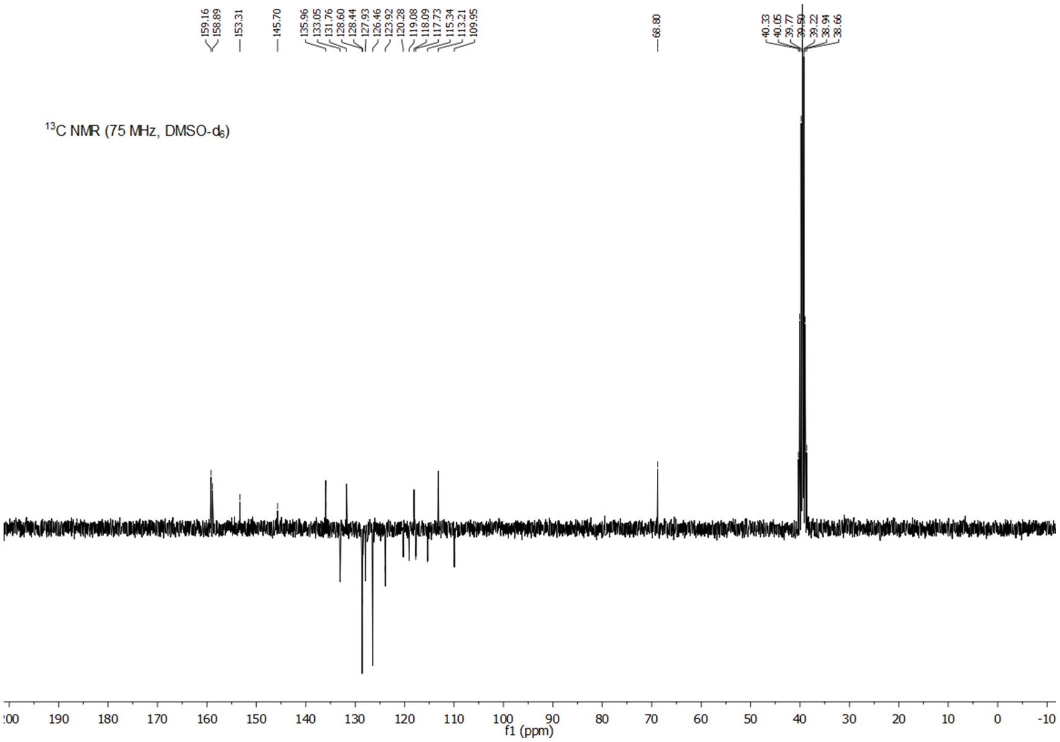

Figure S22. ^{13}C NMR spectrum of **1e**.

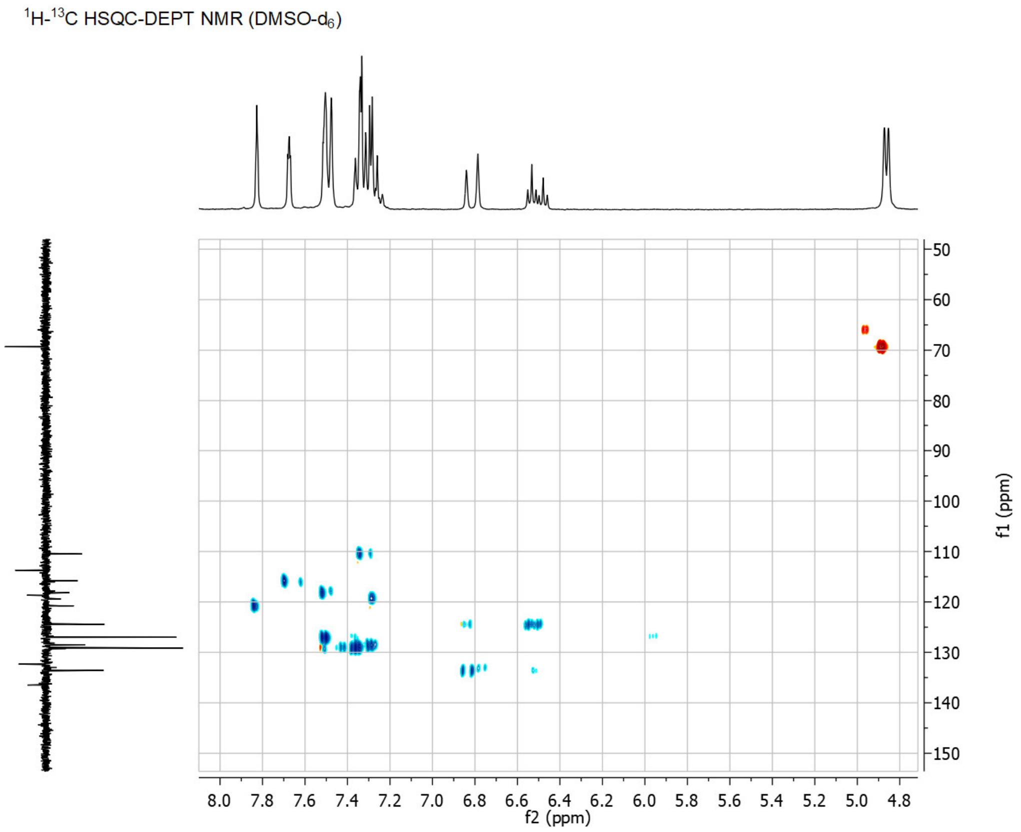

Figure S23. ^{1}H-^{13}C HSQC-DEPT NMR spectrum of **1e**.

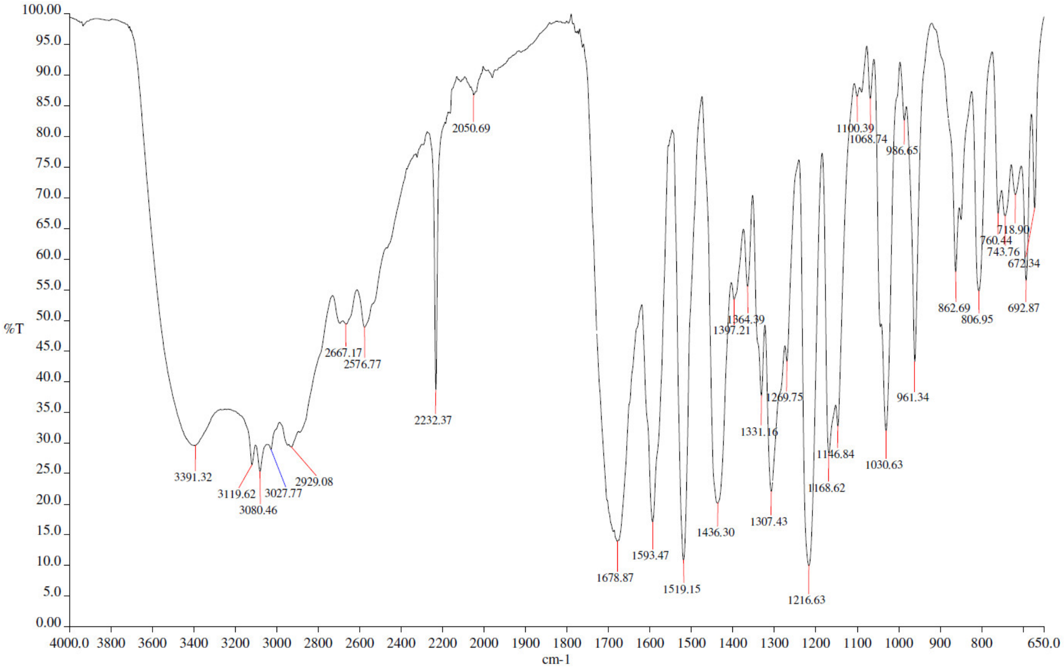

Figure S24. FT-IR spectrum of **1e**.

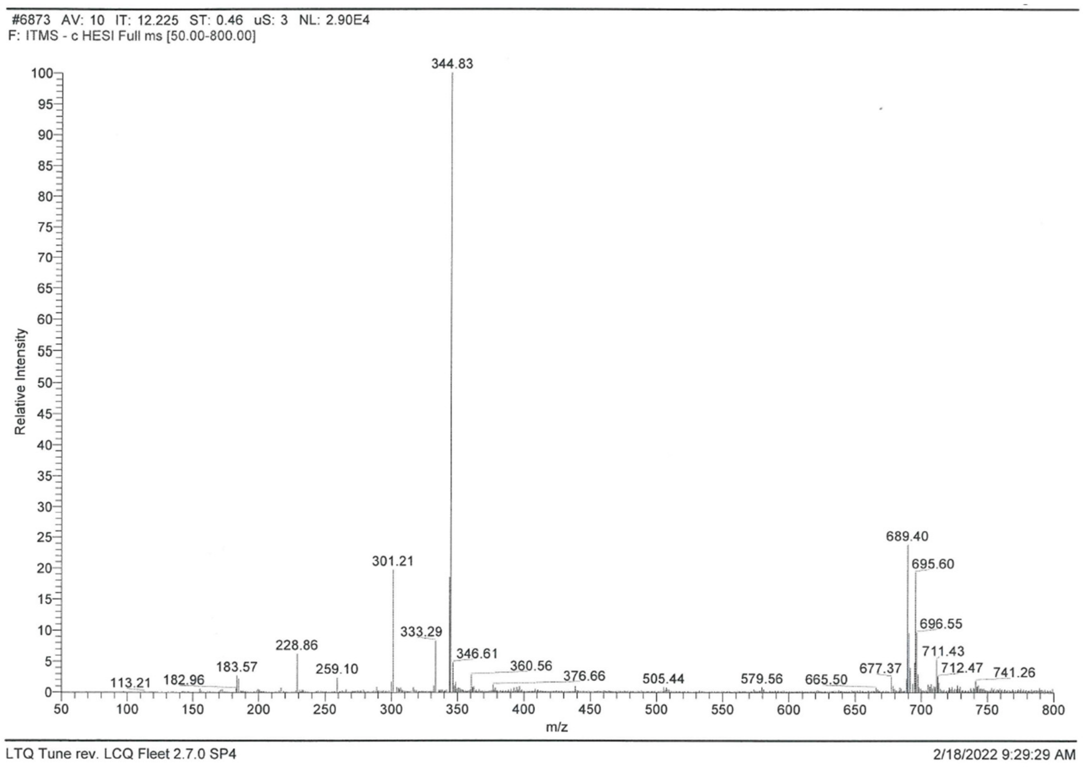

Figure S25. ESI-MS spectrum of **1e**.

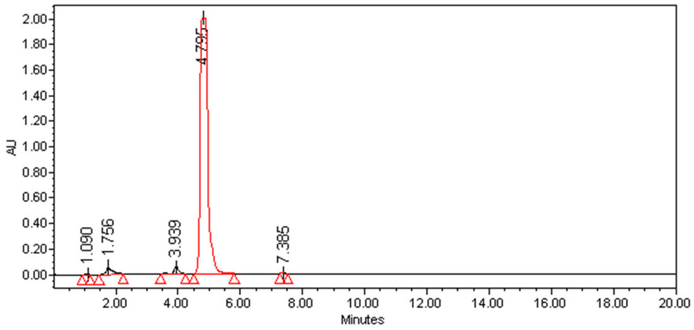

**1e**	
TR (min)	***A (%)***
1.090	0.06
1.756	2.41
3.939	1.81
4.795	95.60
7.385	0.12

Figure S26. HPLC chromatogram of **1e**.

5-(3-Cyano-5-(2-phenoxyethoxy)phenyl)furan-2-carboxylic acid (1f)

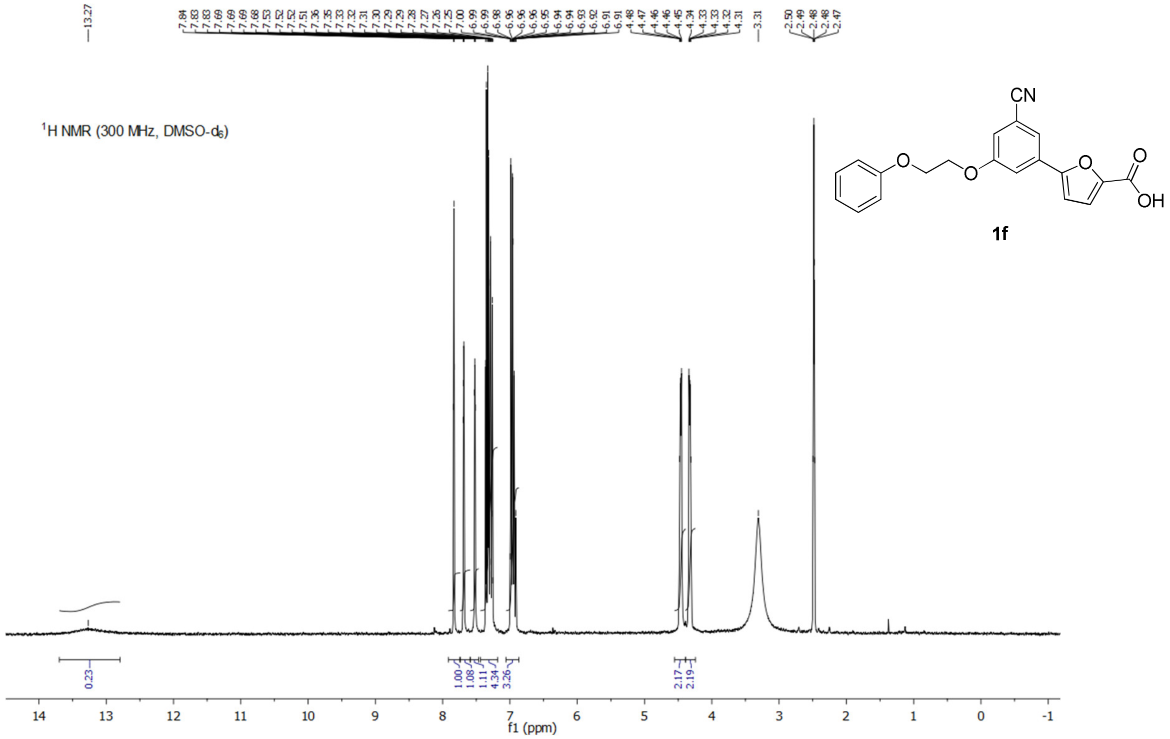

Figure S27. ^{1}H NMR spectrum of **1f**.

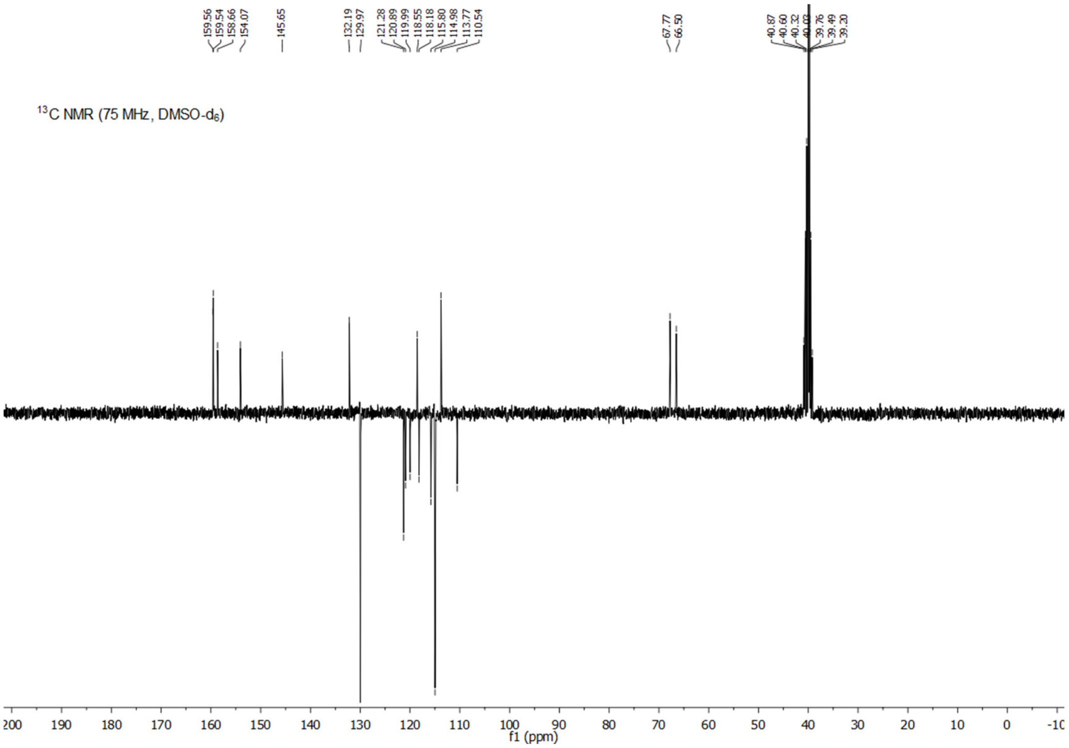

Figure S28. ^{13}C NMR spectrum of **1f**.

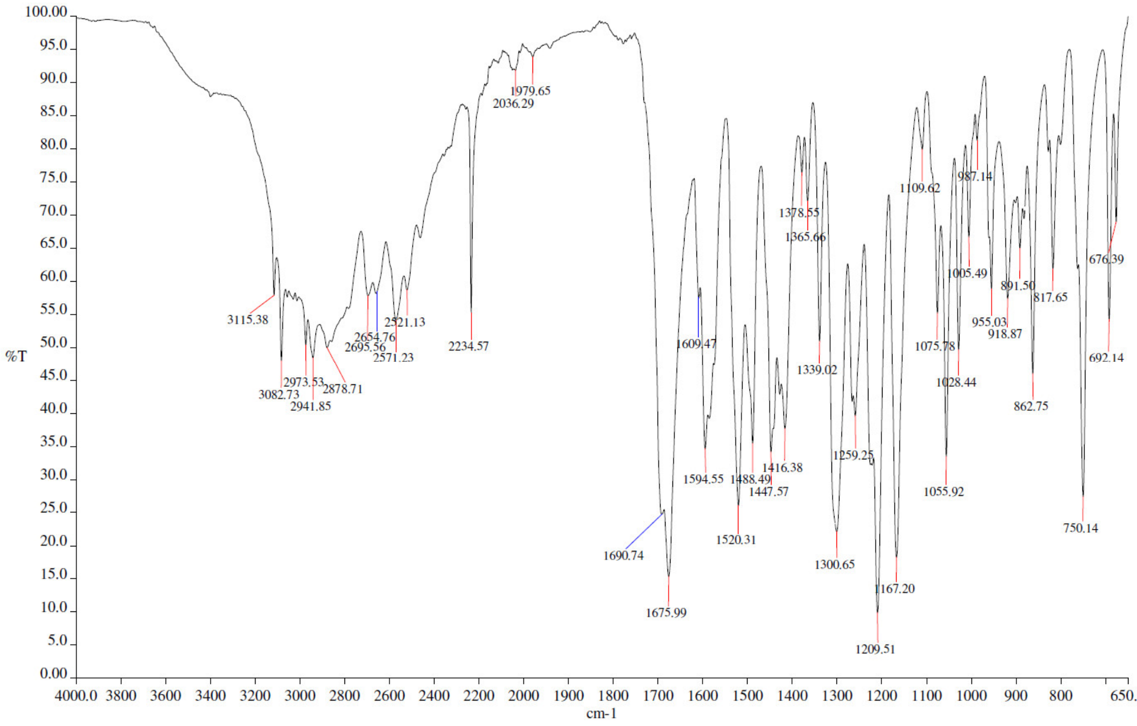

Figure S29. FT-IR spectrum of **1f**.

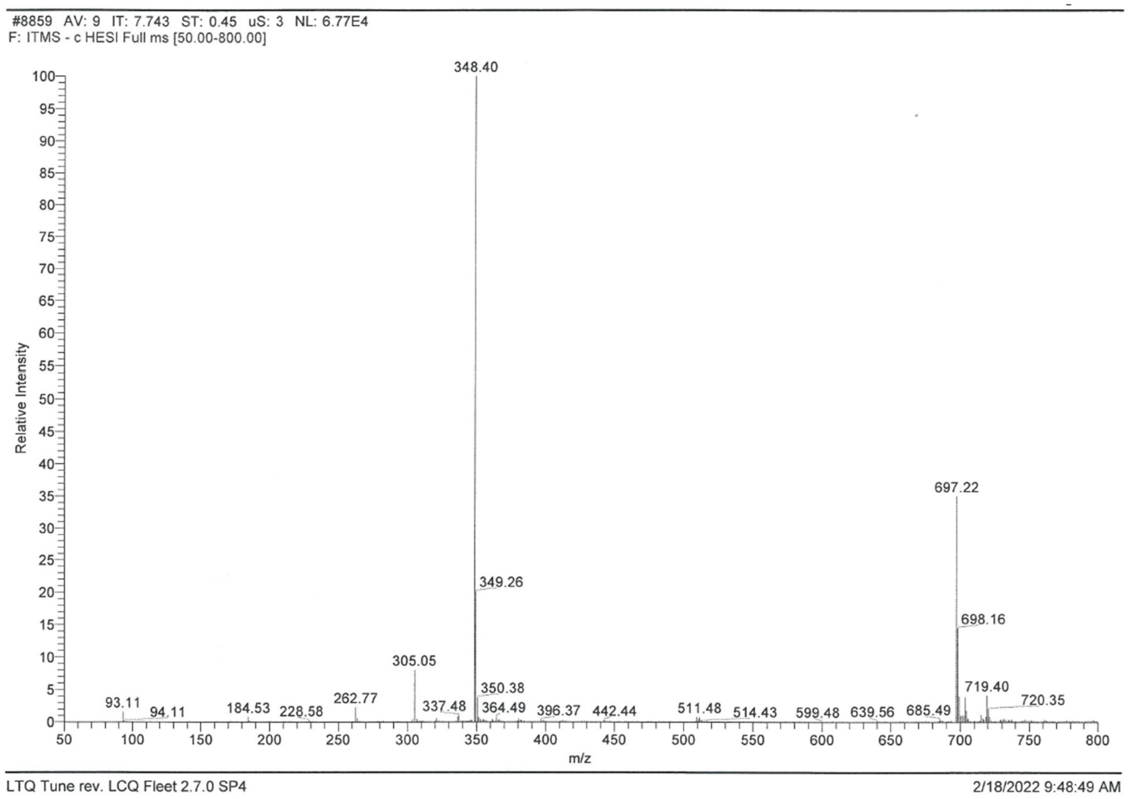

Figure S30. ESI-MS spectrum of **1f**.

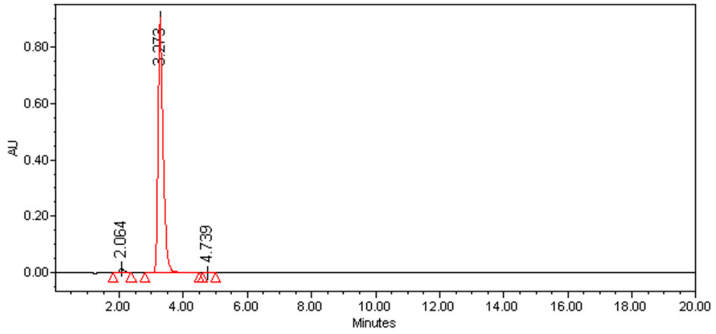

**1f**	
TR (min)	***A (%)***
2.064	1.53
3.273	98.40
4.739	0.07

Figure S31. HPLC chromatogram of **1f**.

5-(3-Cyano-5-(2-(phenylamino)ethoxy)phenyl)furan-2-carboxylic acid (1g)

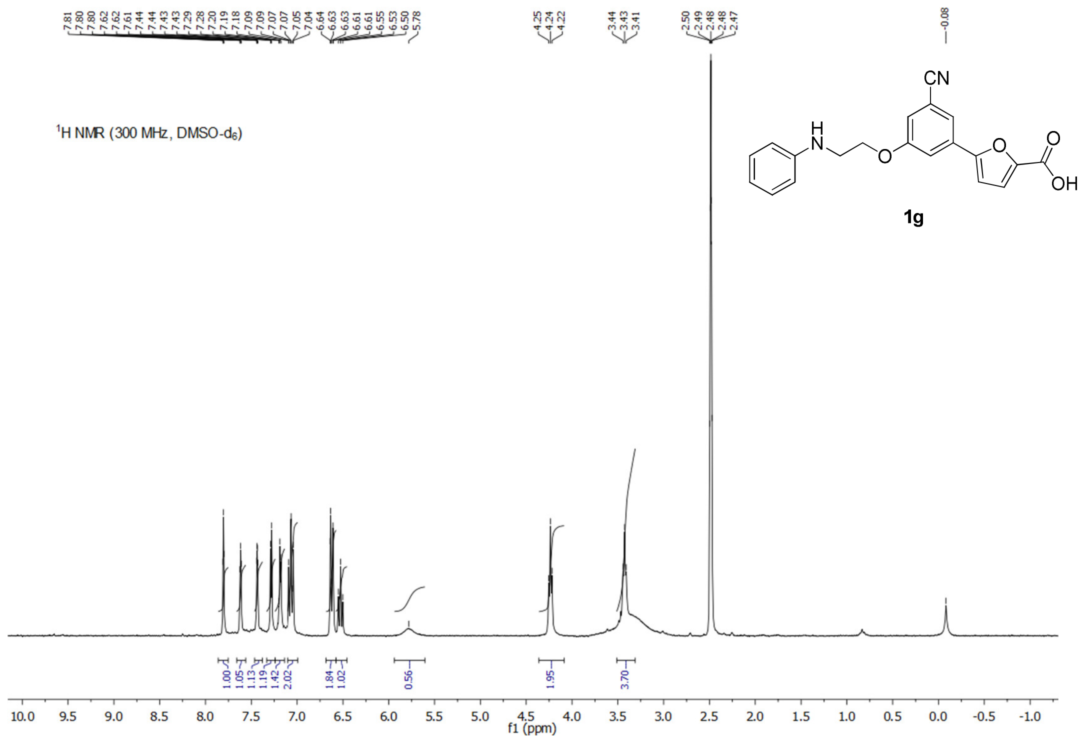

Figure S32. ^{1}H NMR spectrum of **1g**.

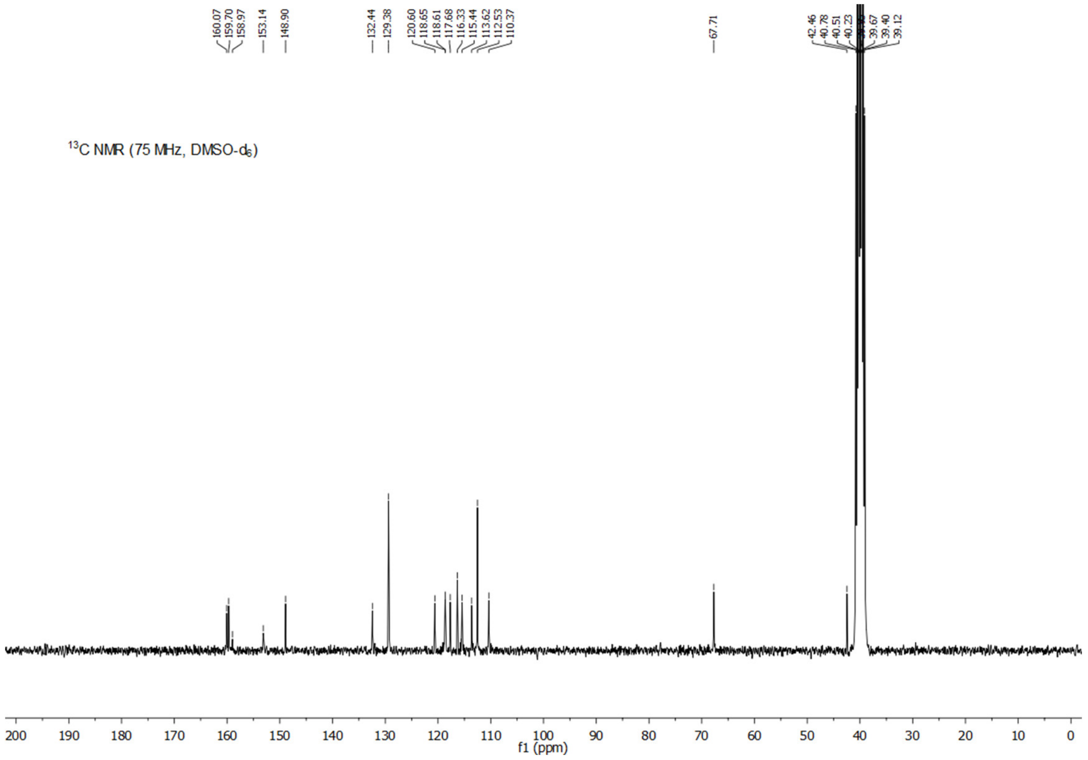

Figure S33. ^{13}C NMR spectrum of **1g**.

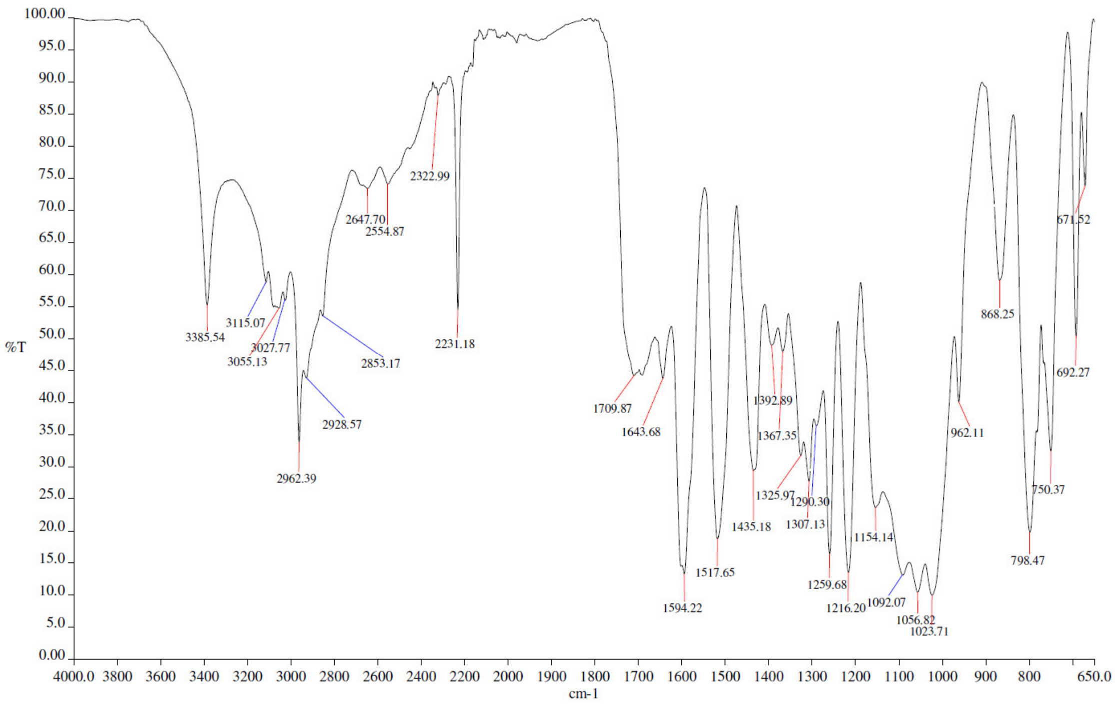

Figure S34. FT-IR spectrum of **1g**.

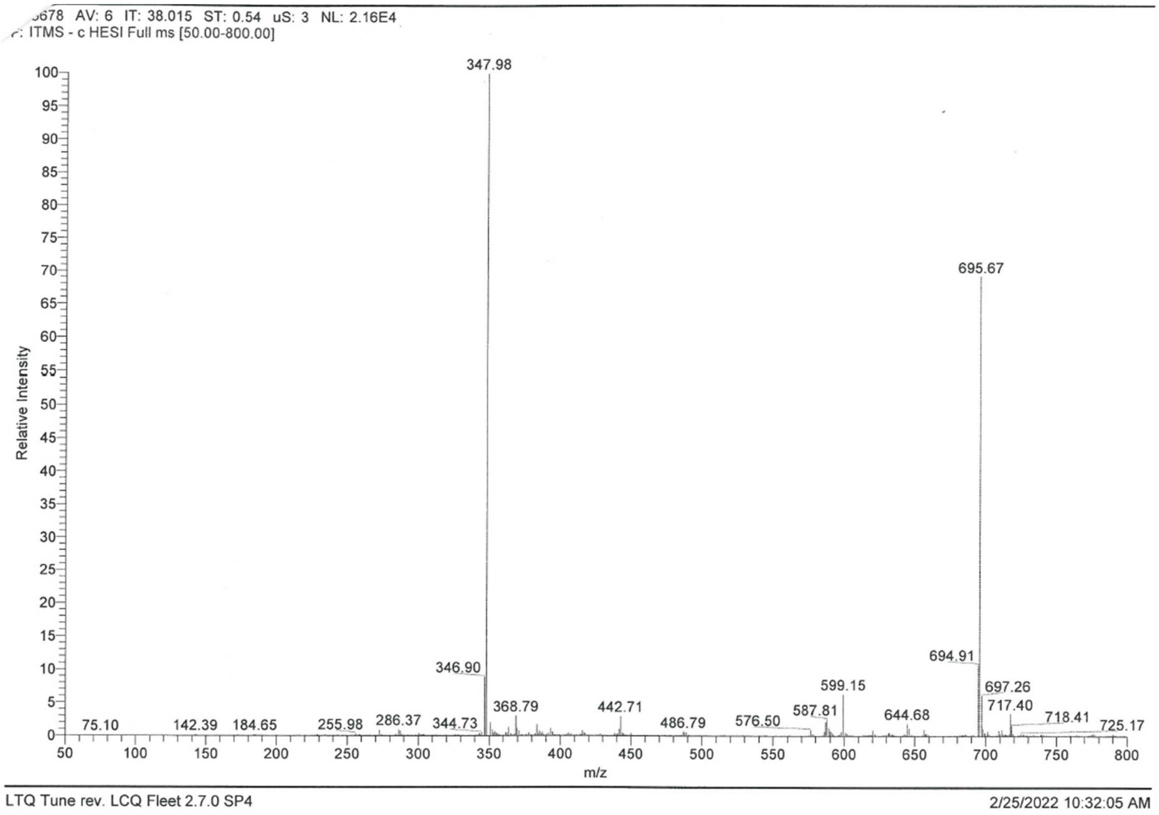

Figure S35. ESI-MS spectrum of **1g**.

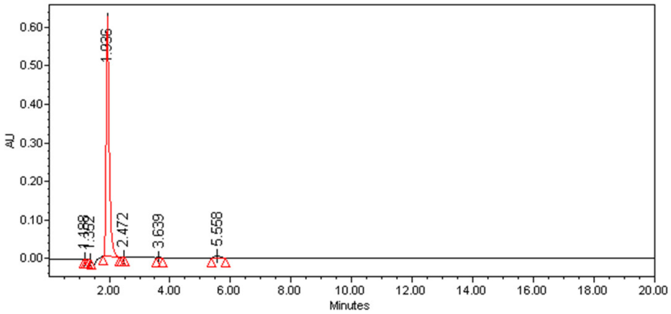

**1g**	
TR (min)	***A (%)***
1.188	0.07
1.352	0.34
1.936	97.64
2.472	0.09
3.639	0.09
5.558	1.77

Figure S36. HPLC chromatogram of **1g**.

5-(3-Cyano-5-(naphthalen-2-yloxy)phenyl)furan-2-carboxylic acid (1h)

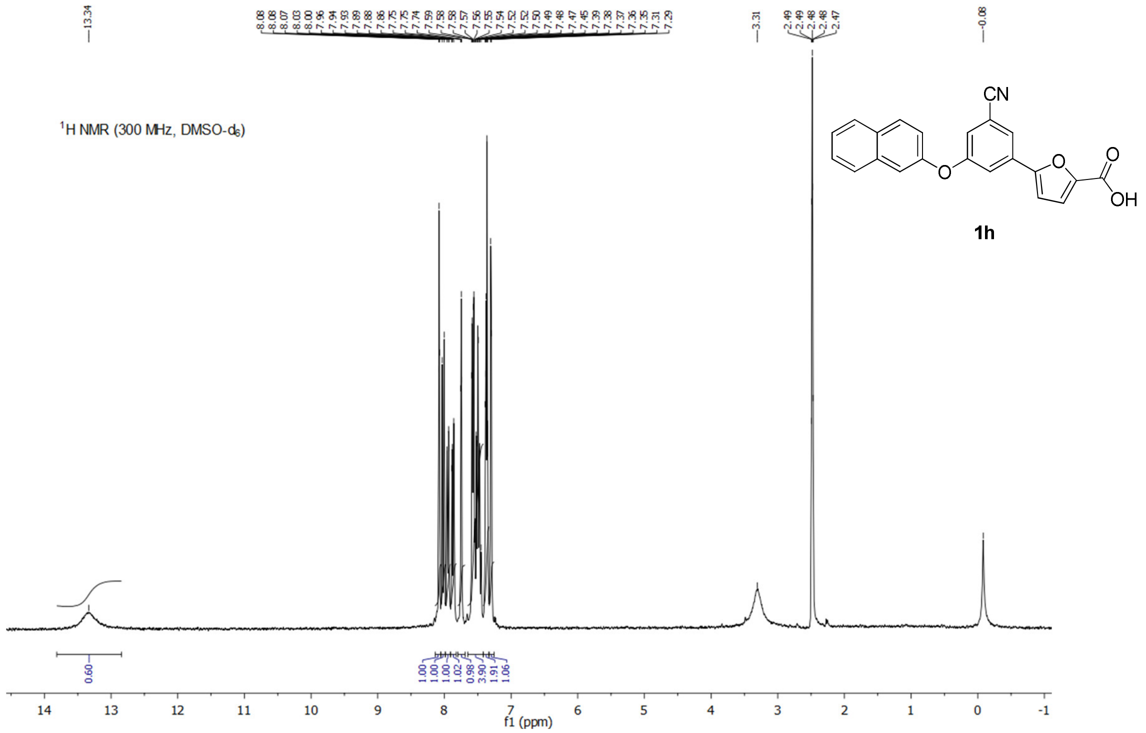

Figure S37. ^{1}H NMR spectrum of **1h**.

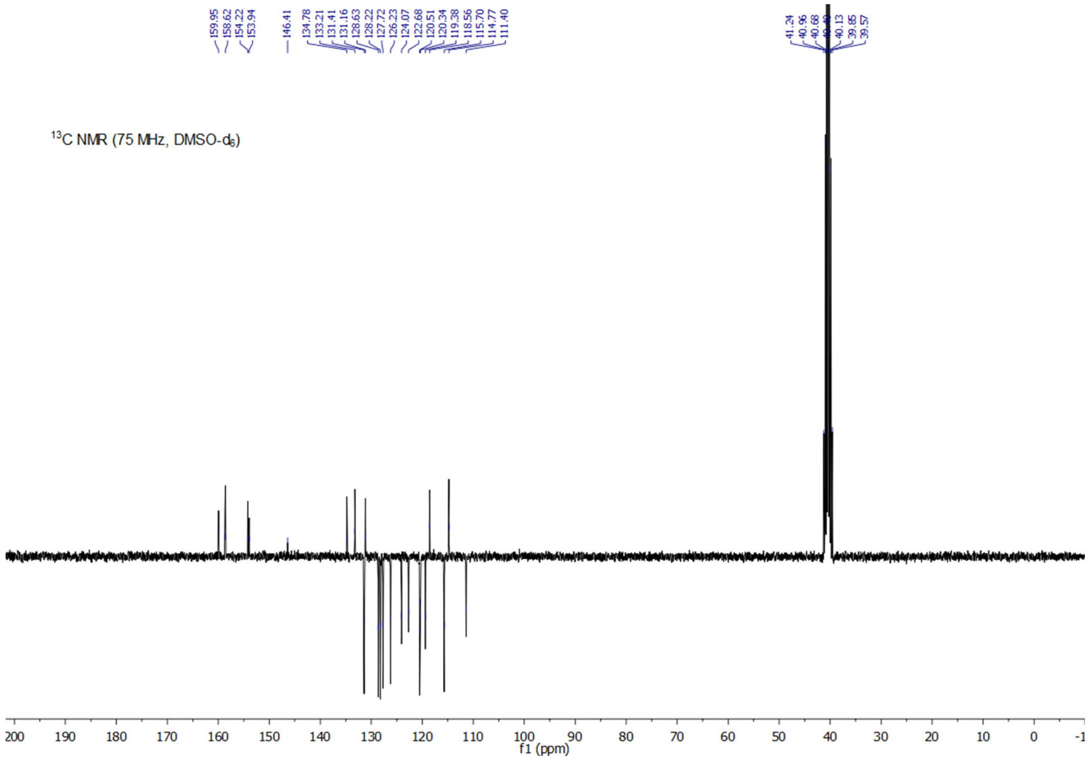

Figure S38. ^{13}C NMR spectrum of **1h**.

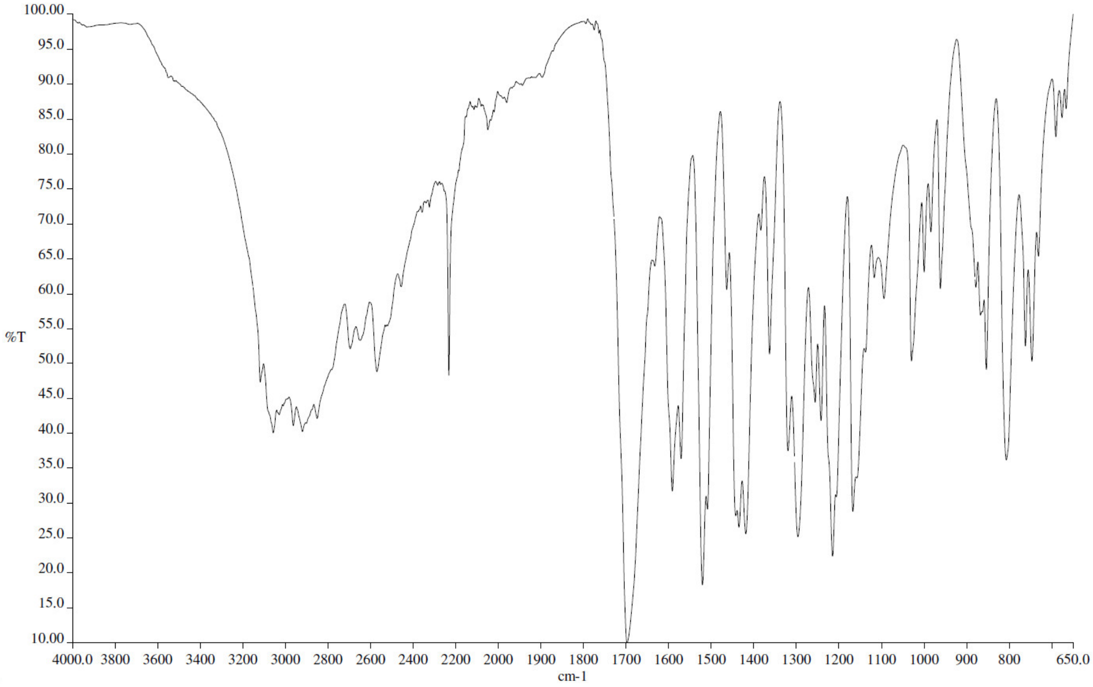

Figure S39. FT-IR spectrum of **1h**.

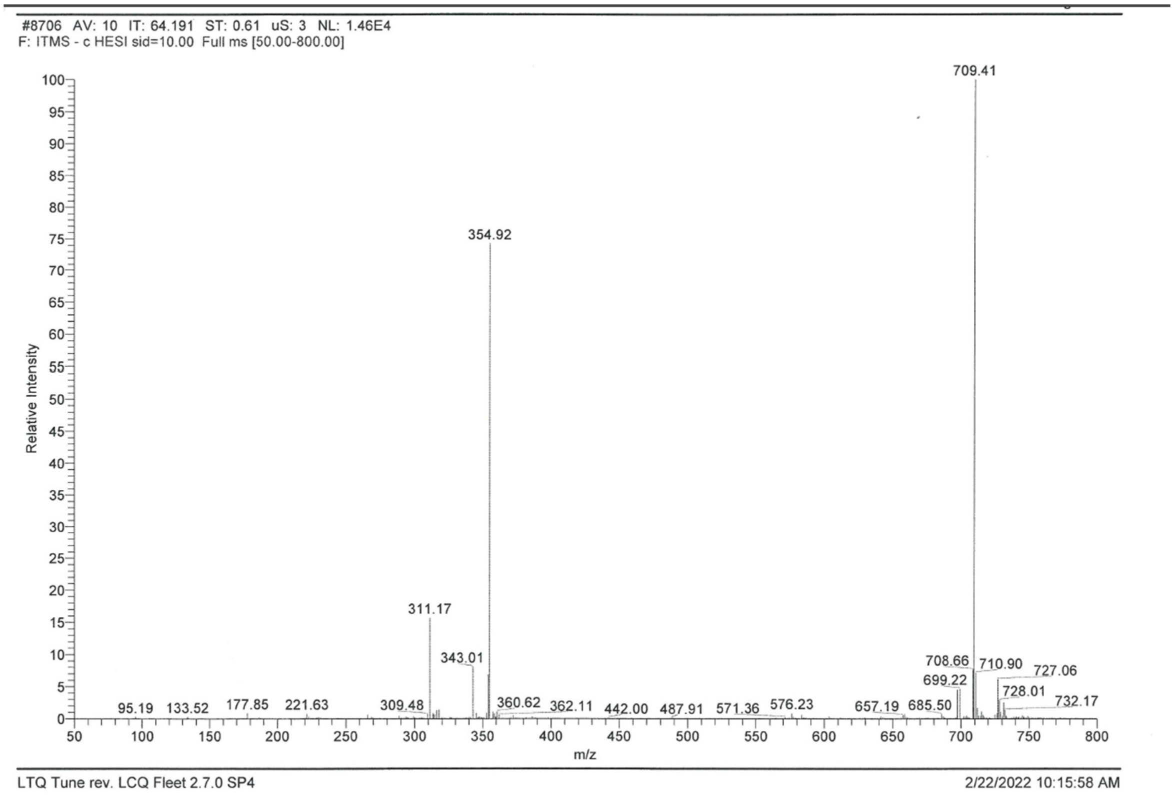

Figure S40. ESI-MS spectrum of **1h**.

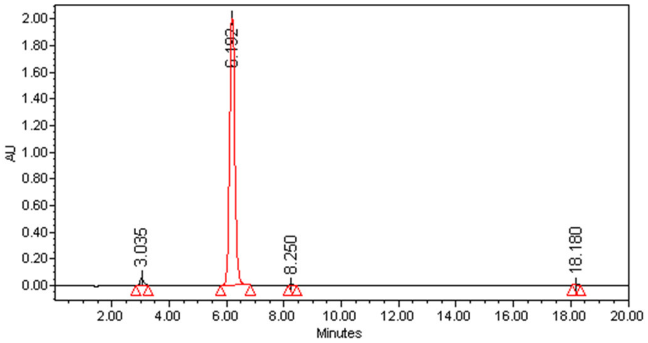

1h	
TR (min)	***A (%)***
3.035	1.88
6.192	97.89
8.250	0.12
18.180	0.10

Figure S41. HPLC chromatogram of **1h**.

5-(3-Cyano-5-(quinolin-7-yloxy)phenyl)furan-2-carboxylic acid (1i)

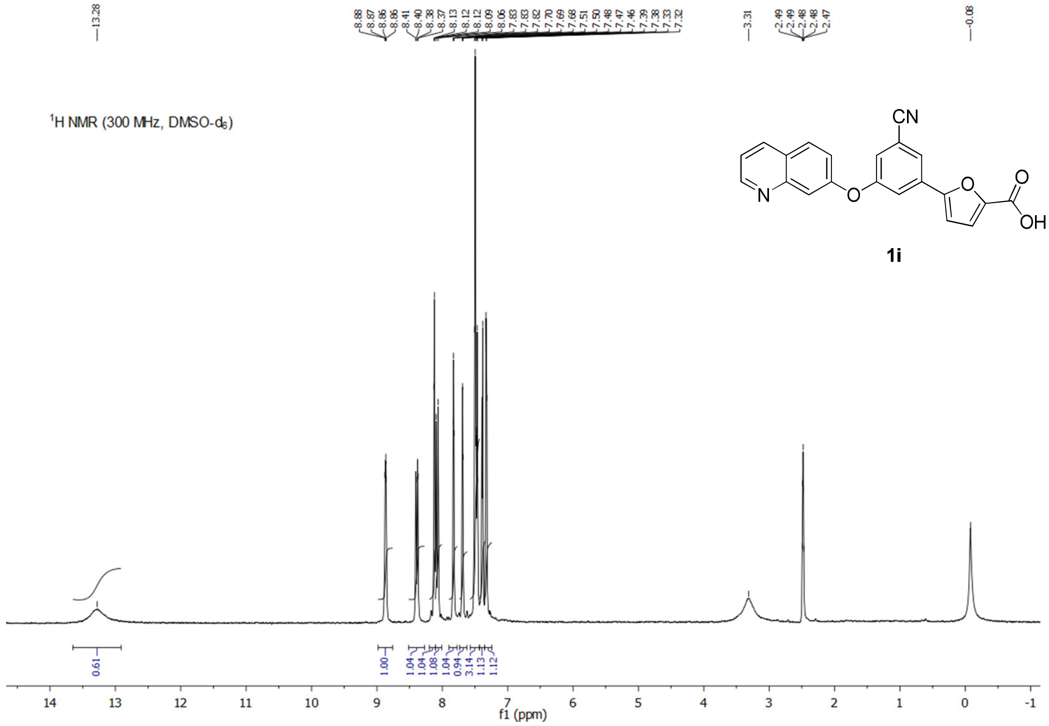

Figure S42. ^{1}H NMR spectrum of **1i**.

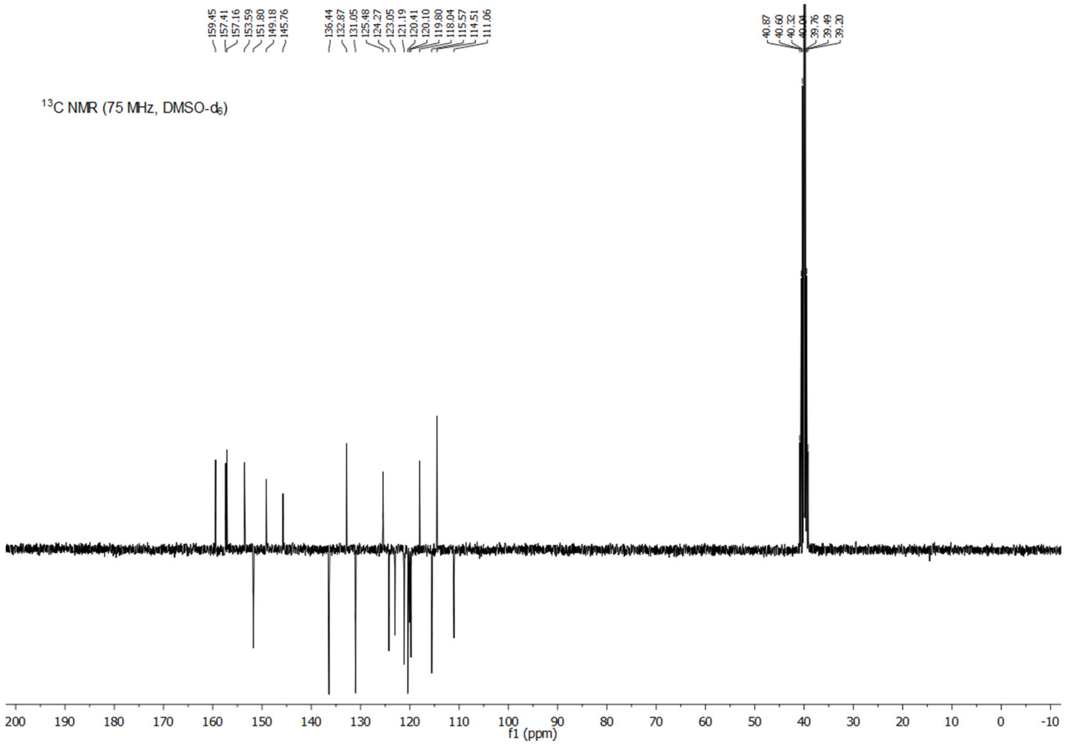

Figure S43. ^{13}C NMR spectrum of **1i**.

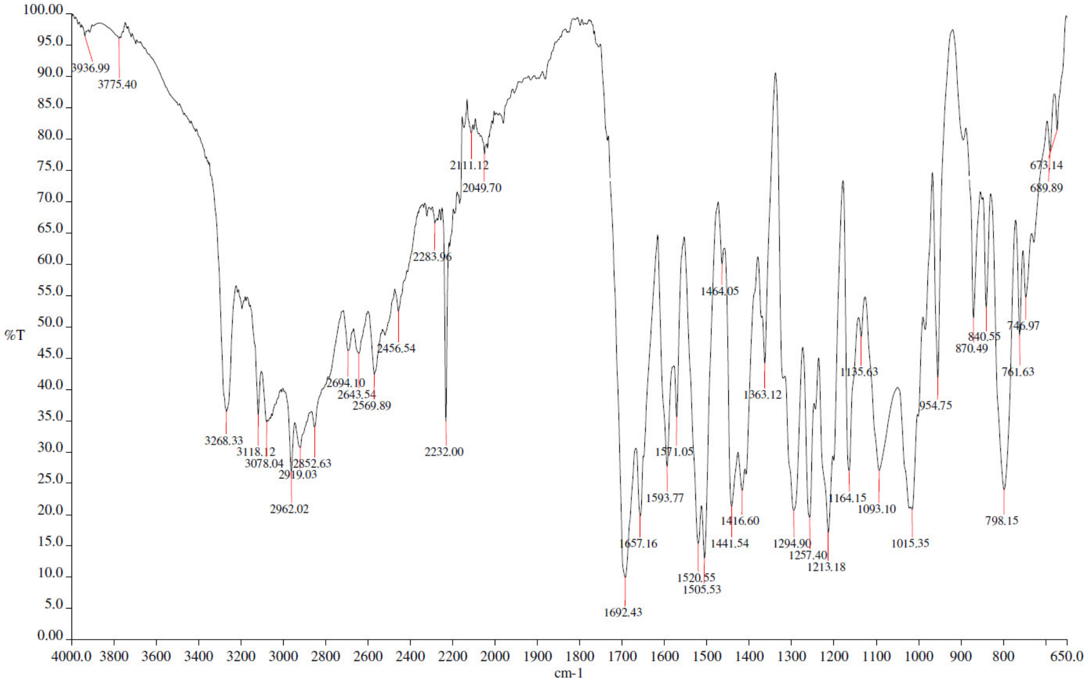

Figure S44. FT-IR spectrum of **1i**.

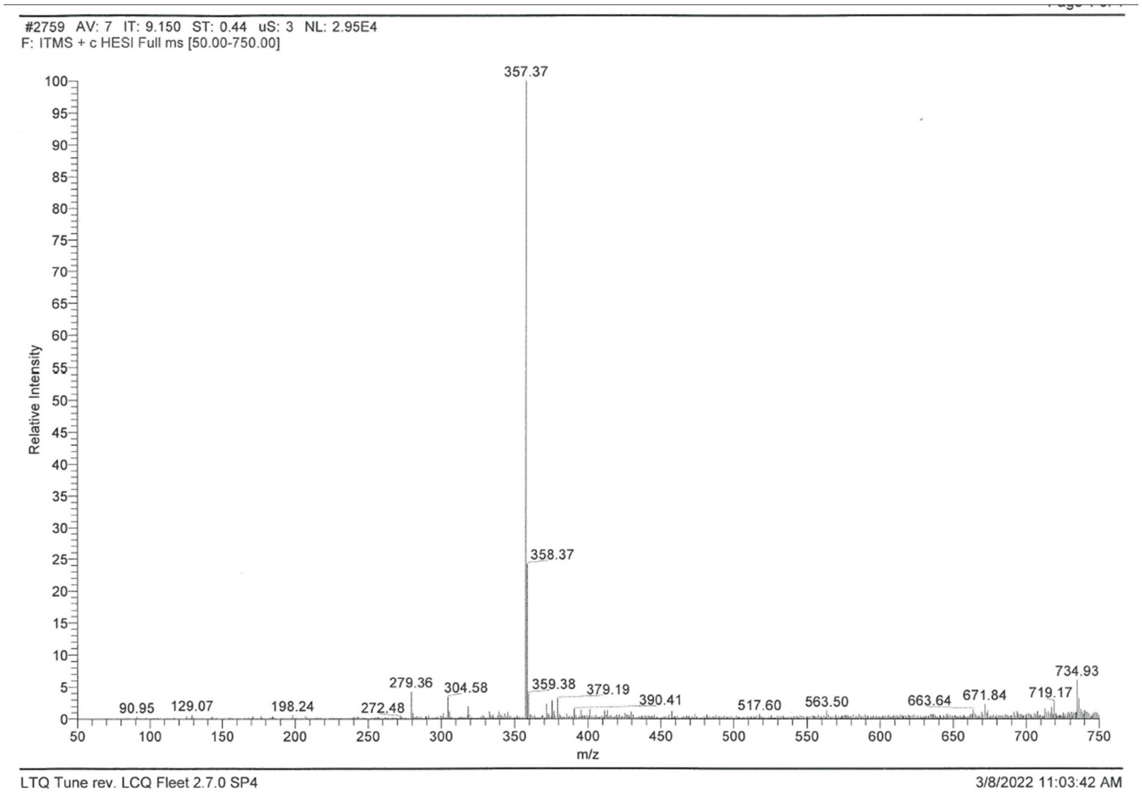

Figure S45. ESI-MS spectrum of **1i**.

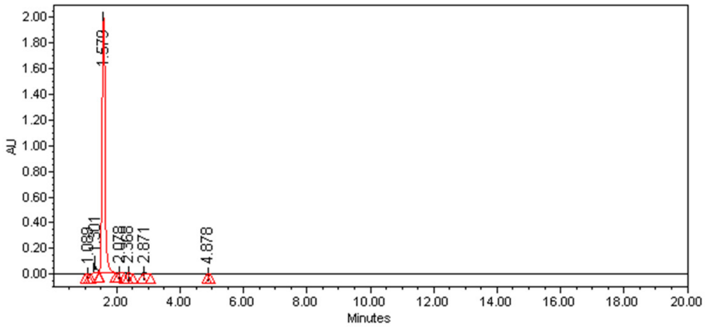

1i	
TR (min)	***A (%)***
1.089	0.04
1.301	3.05
1.579	96.53
2.078	0.07
2.368	0.03
2.871	0.26
4.878	0.02

Figure S46. HPLC chromatogram of **1i**.

5-(3-Cyano-5-(naphthalen-2-ylmethoxy)phenyl)furan-2-carboxylic acid (1j)

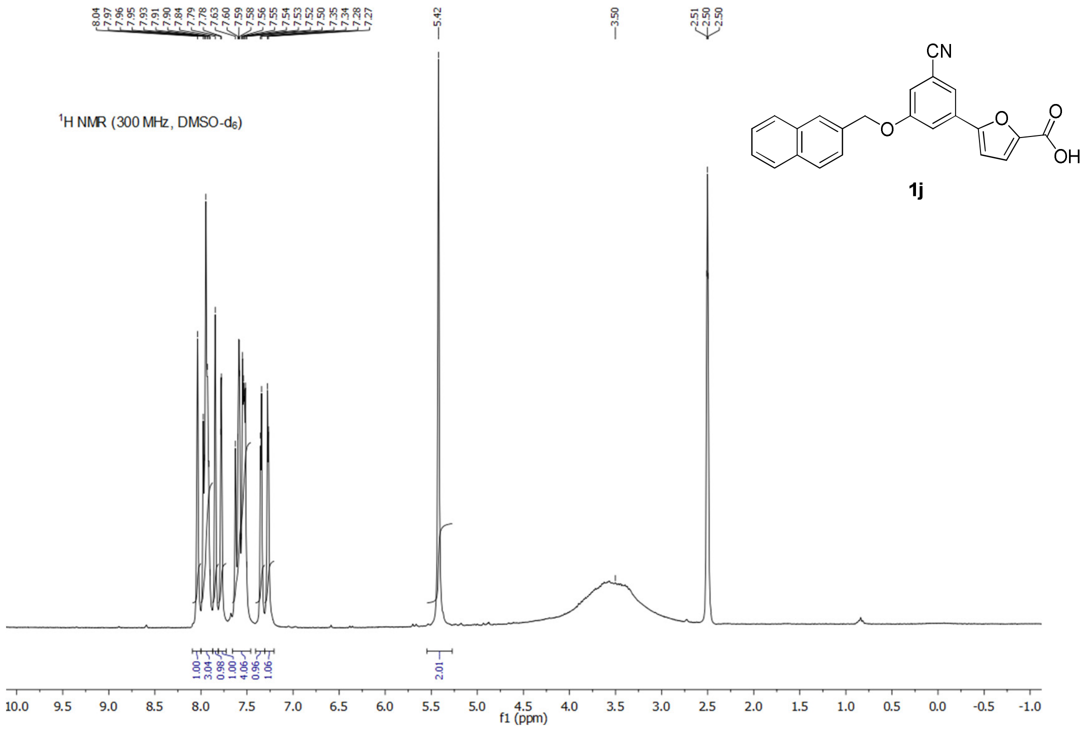

Figure S47. ^{1}H NMR spectrum of **1j**.

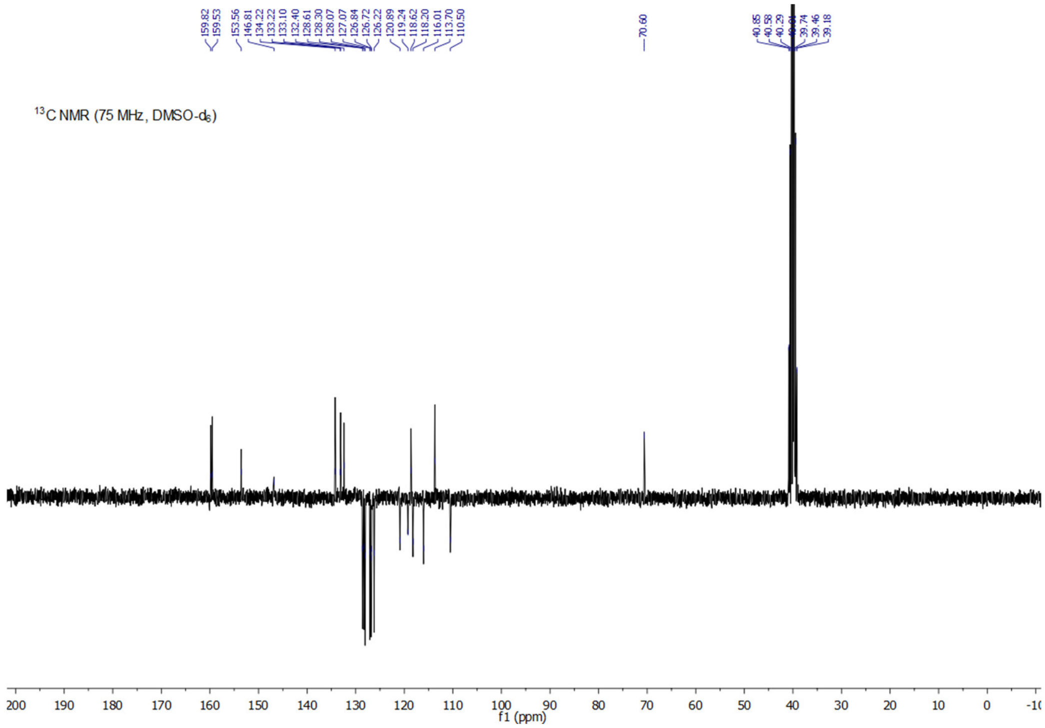

Figure S48. ^{13}C NMR spectrum of **1j**.

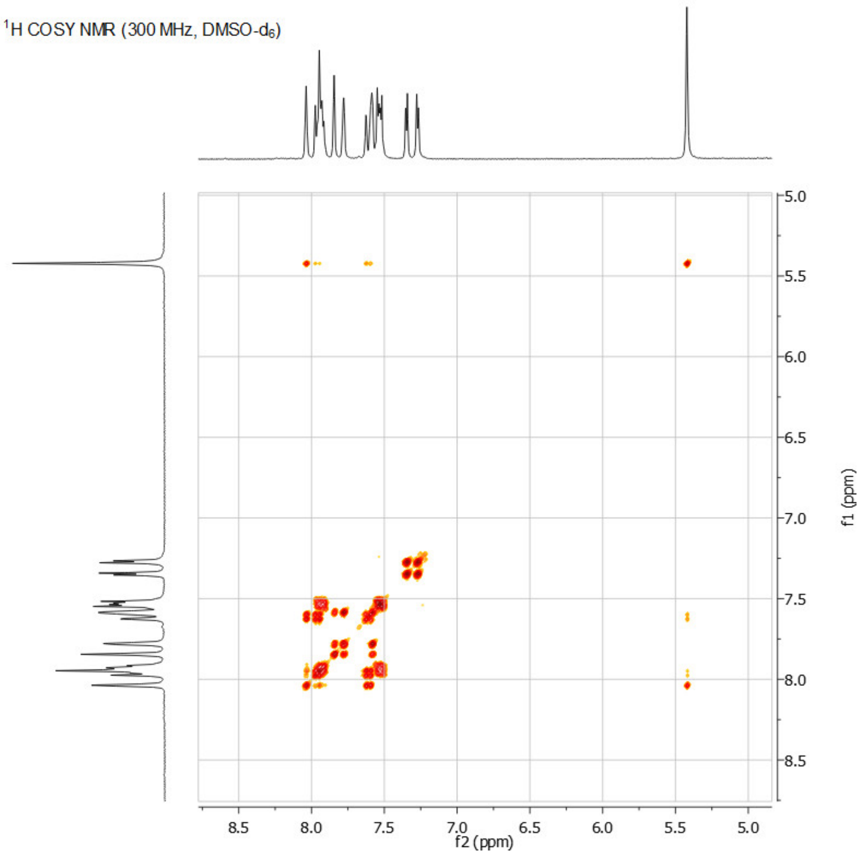

Figure S49. ^{1}H-^{1}H COSY NMR spectrum of **1j**.

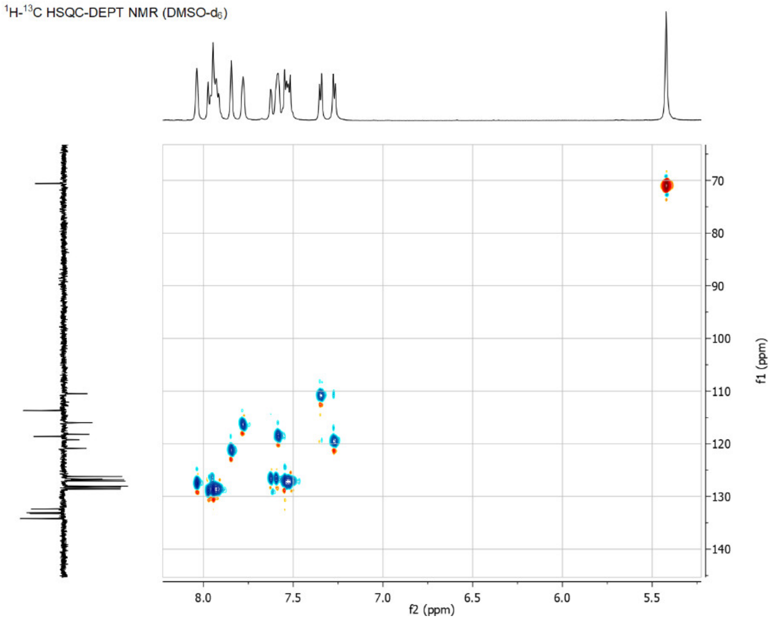

Figure S50. ^{1}H-^{13}C HSQC-DEPT NMR spectrum of **1j**.

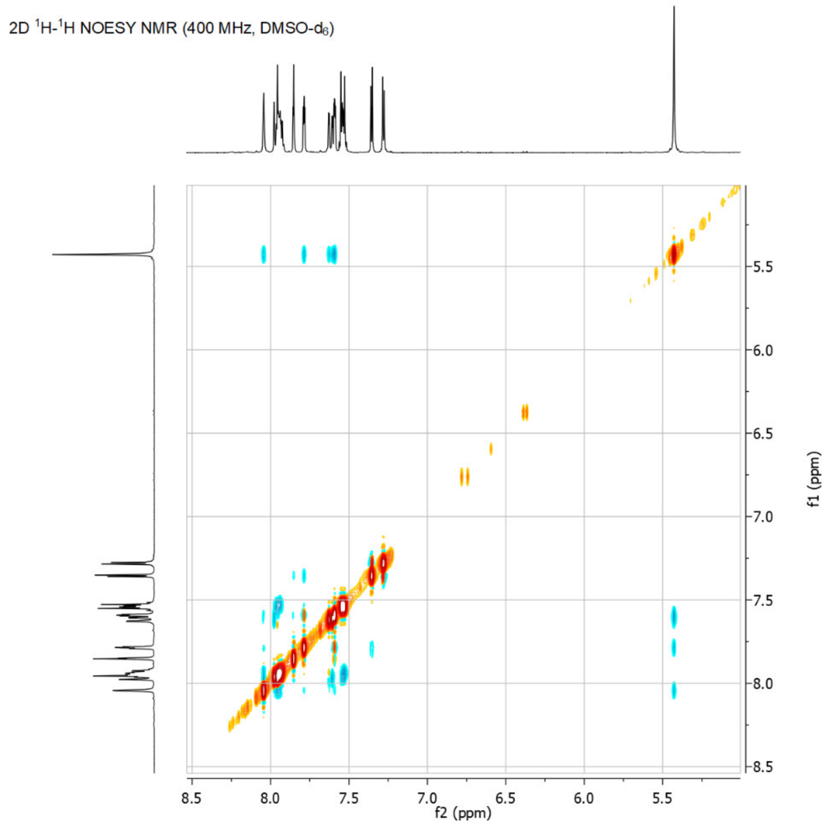

Figure S51. 2D ^{1}H-^{1}H NOESY NMR spectrum of **1j**.

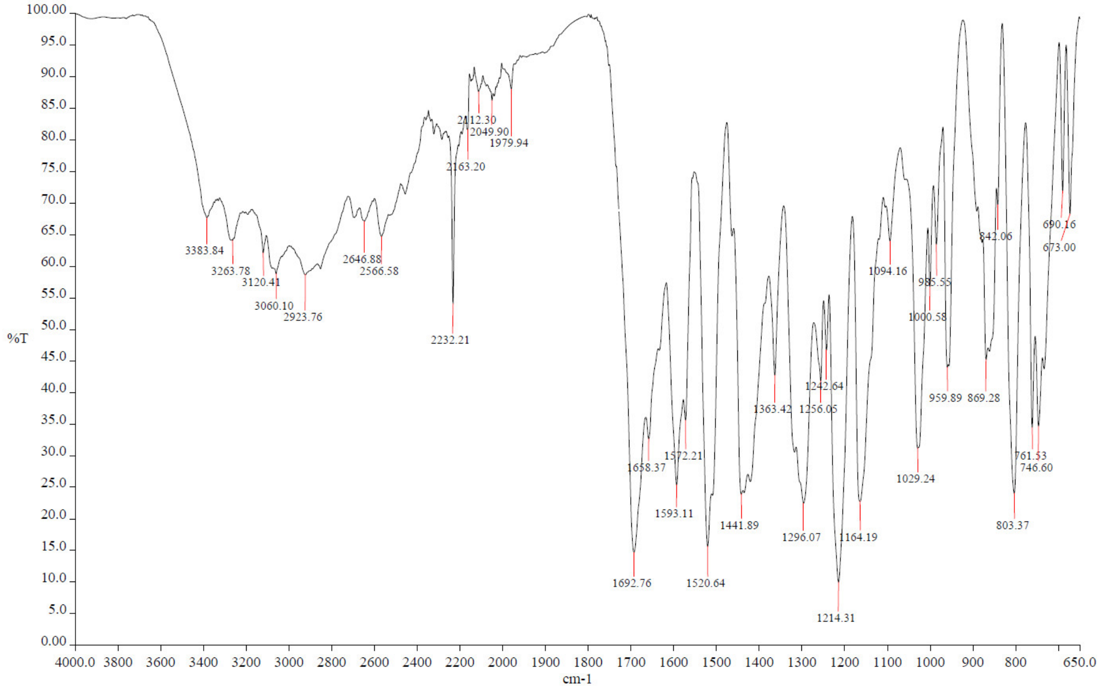

Figure S52. FT-IR spectrum of **1j**.

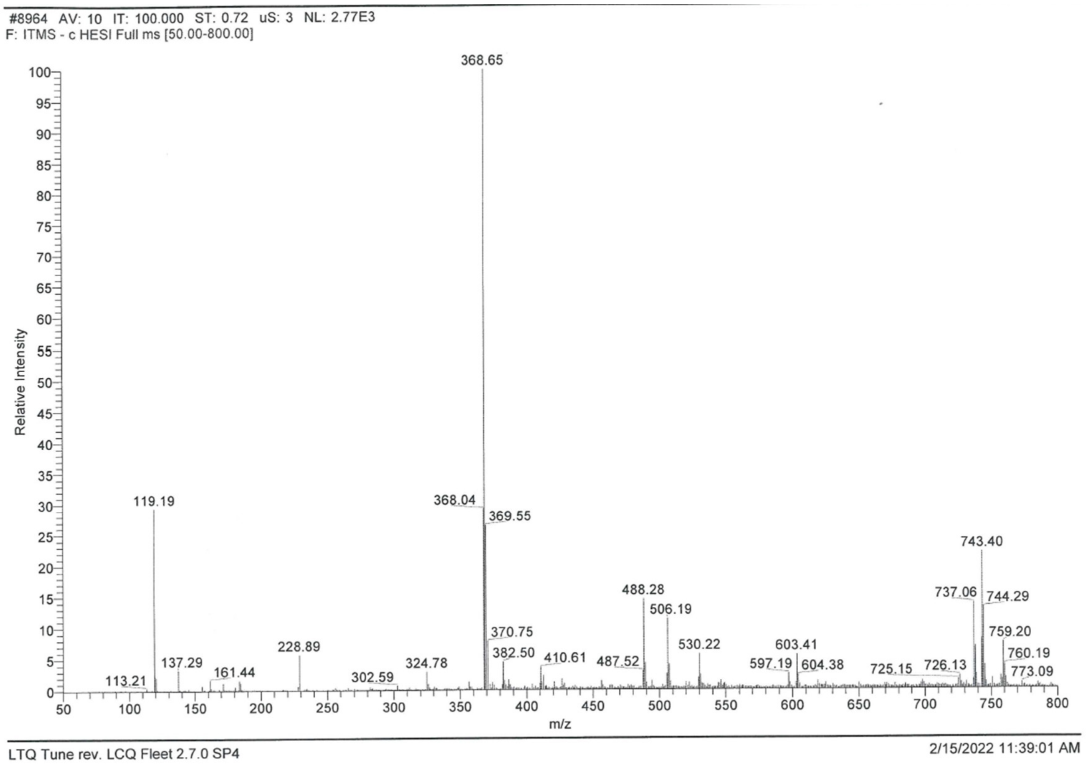

Figure S53. ESI-MS spectrum of **1j**.

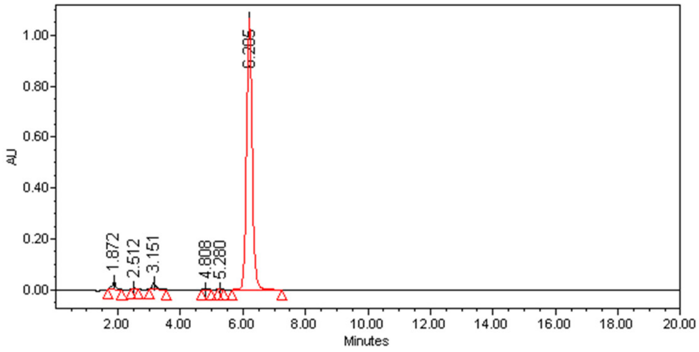

1j	
TR (min)	***A (%)***
1.872	1.78
2.512	0.26
3.151	1.65
4.808	0.15
5.280	0.06
6.205	96.07

Figure S54. HPLC chromatogram of **1j**.

Methyl 5-(3-cyano-5-phenoxyphenyl)furan-2-carboxylate (2a)

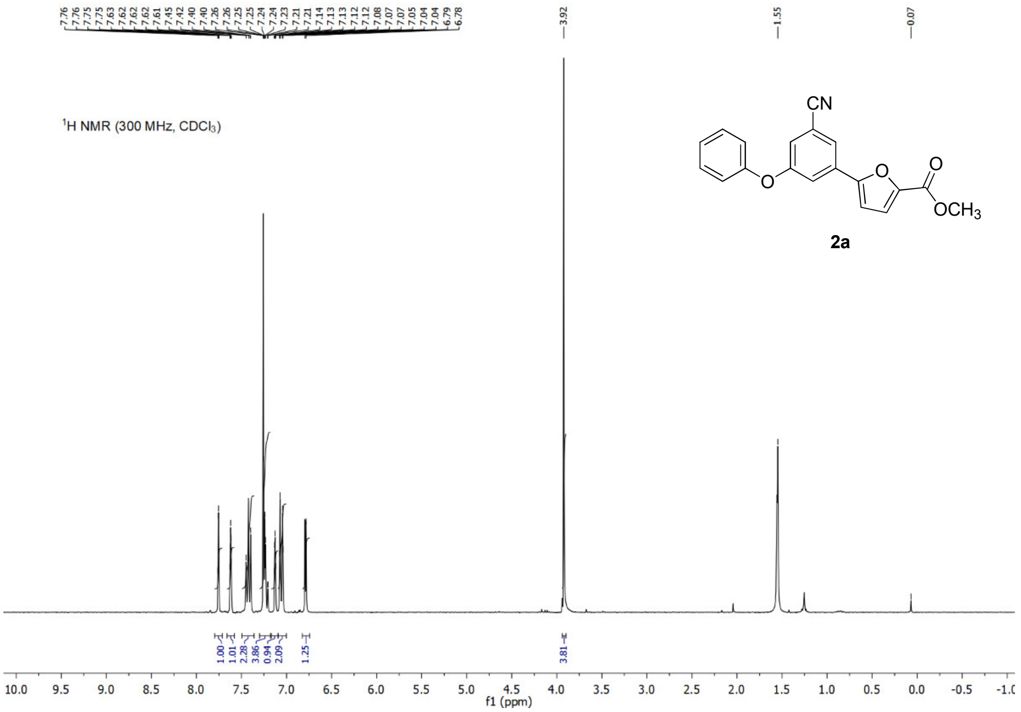

Figure S55. ^{1}H NMR spectrum of **2a**.

Methyl 5-(3-(benzyloxy)-5-cyanophenyl)furan-2-carboxylate (2b)

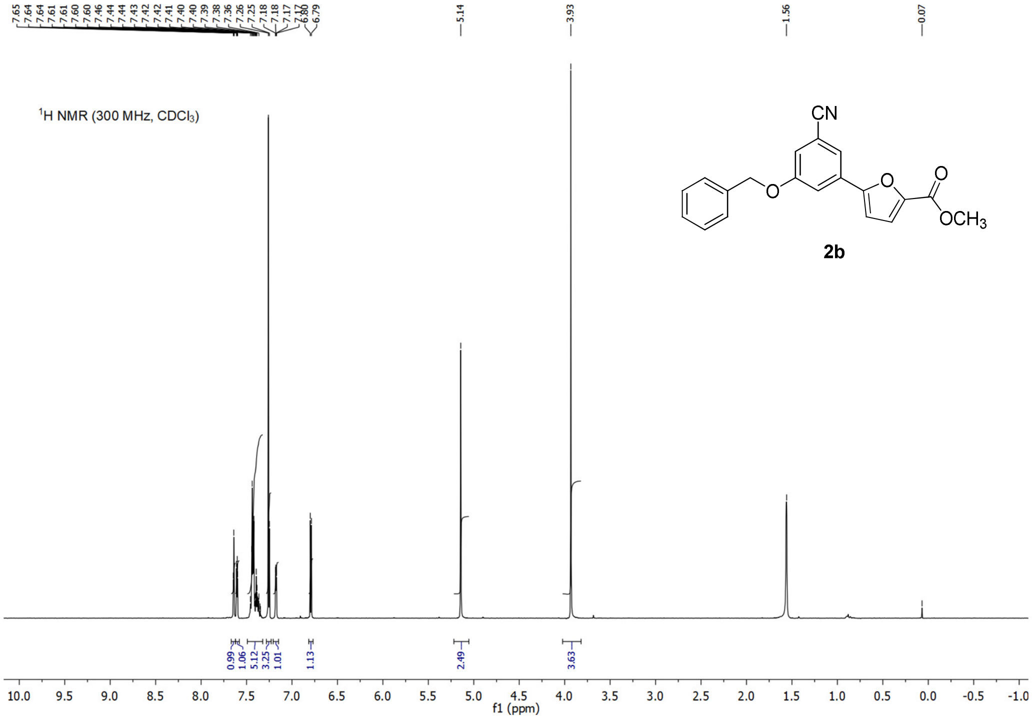

Figure S56. ^{1}H NMR spectrum of **2b**.

Methyl 5-(3-cyano-5-phenethoxyphenyl)furan-2-carboxylate (2c)

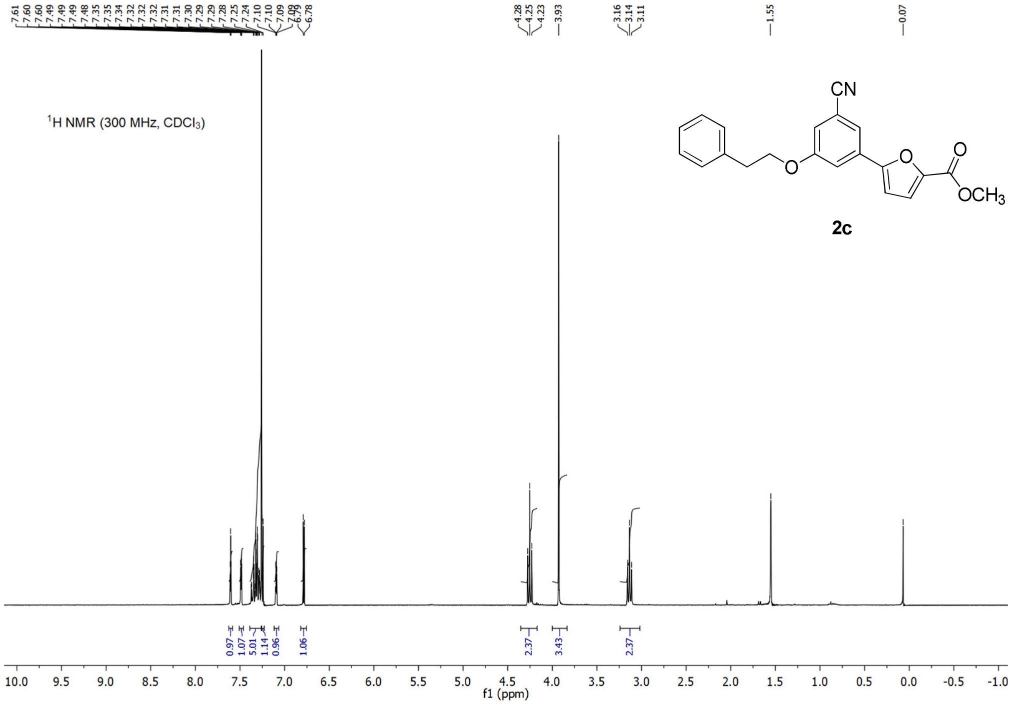

Figure S57. ^{1}H NMR spectrum of **2c**.

Methyl 5-(3-cyano-5-(3-phenylpropoxy)phenyl)furan-2-carboxylate (2d)

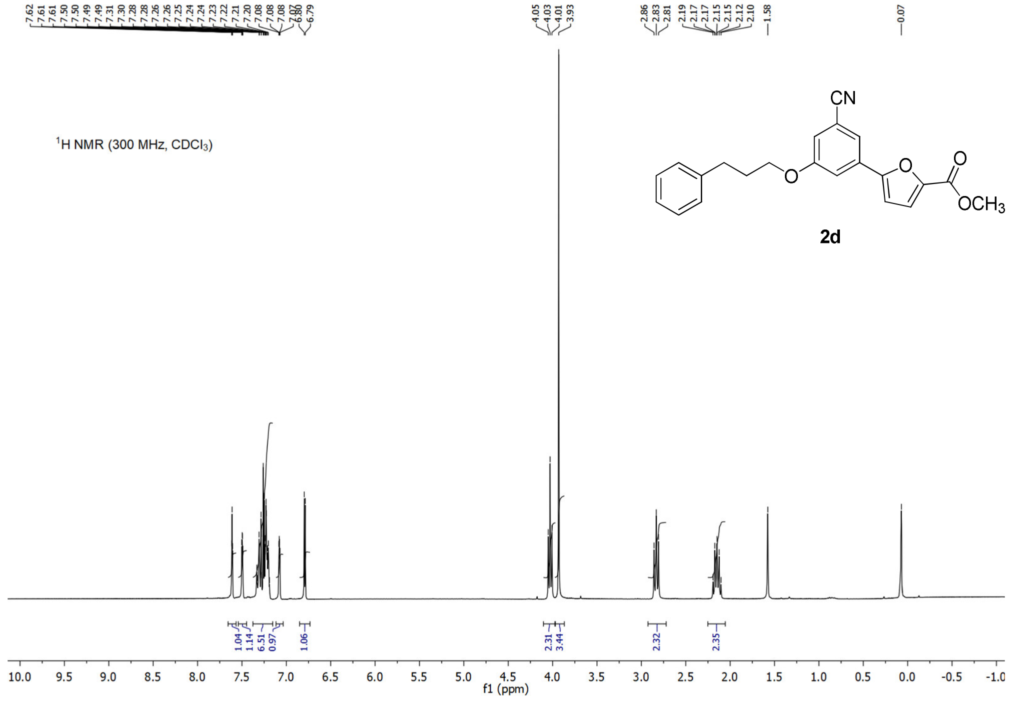

Figure S58. ^{1}H NMR spectrum of **2d**.

(*E*)-Methyl 5-(3-(cinnamyloxy)-5-cyanophenyl)furan-2-carboxylate (2e)

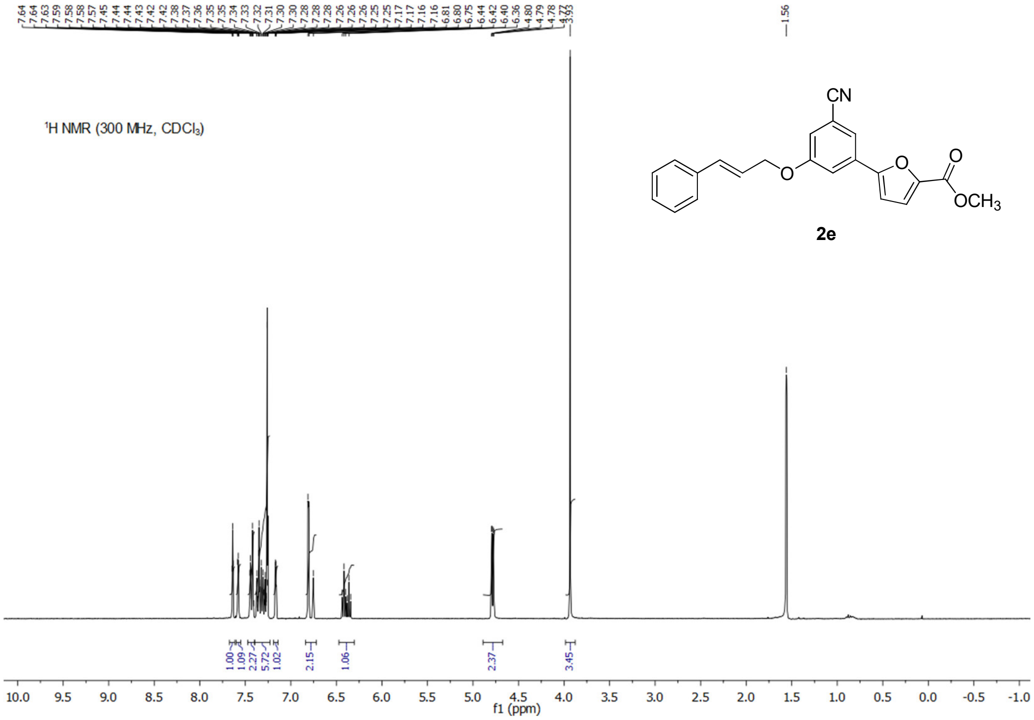

Figure S59. ^{1}H NMR spectrum of **2e**.

Methyl 5-(3-cyano-5-(2-phenoxyethoxy)phenyl)furan-2-carboxylate (2f)

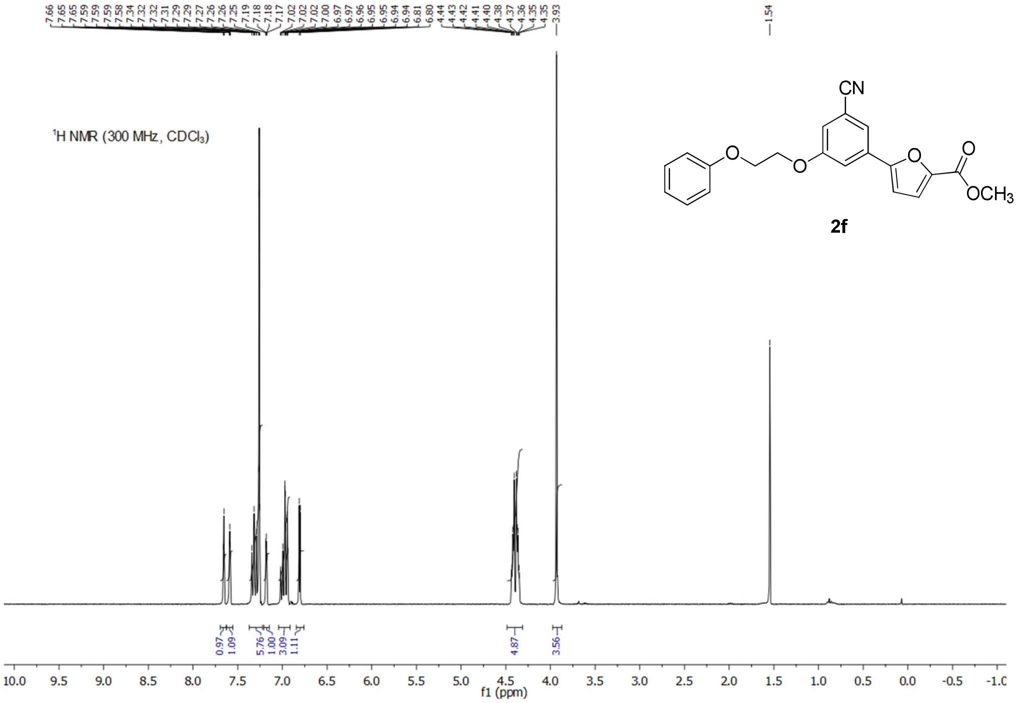

Figure S60. ^{1}H NMR spectrum of **2f**.

Methyl 5-(3-cyano-5-(2-(phenylamino)ethoxy)phenyl)furan-2-carboxylate (2g)

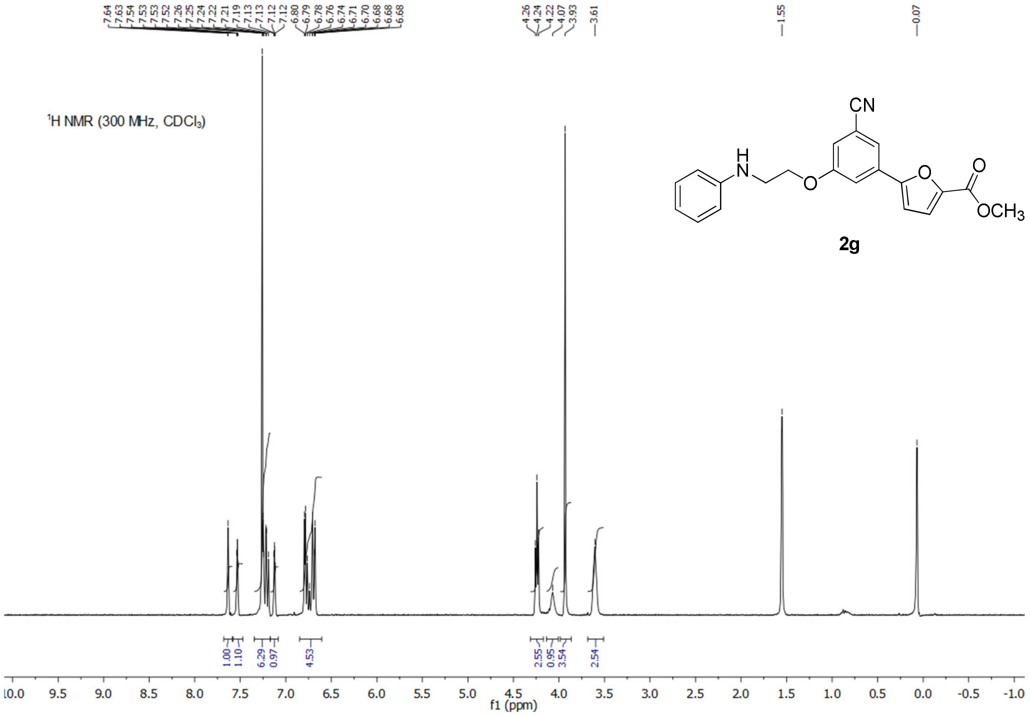

Figure S61. ^{1}H NMR spectrum of **2g**.

Methyl 5-(3-cyano-5-(naphthalen-2-yloxy)phenyl)furan-2-carboxylate (2h)

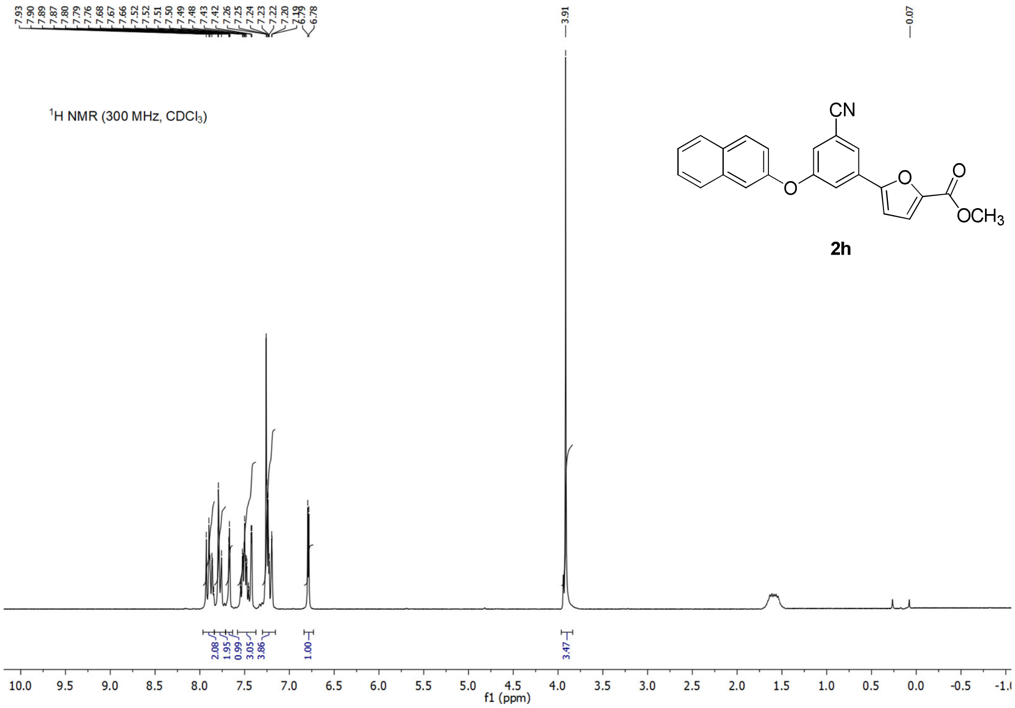

Figure S62. ^{1}H NMR spectrum of **2h**.

Methyl 5-(3-cyano-5-(quinolin-7-yloxy)phenyl)furan-2-carboxylate (2i)

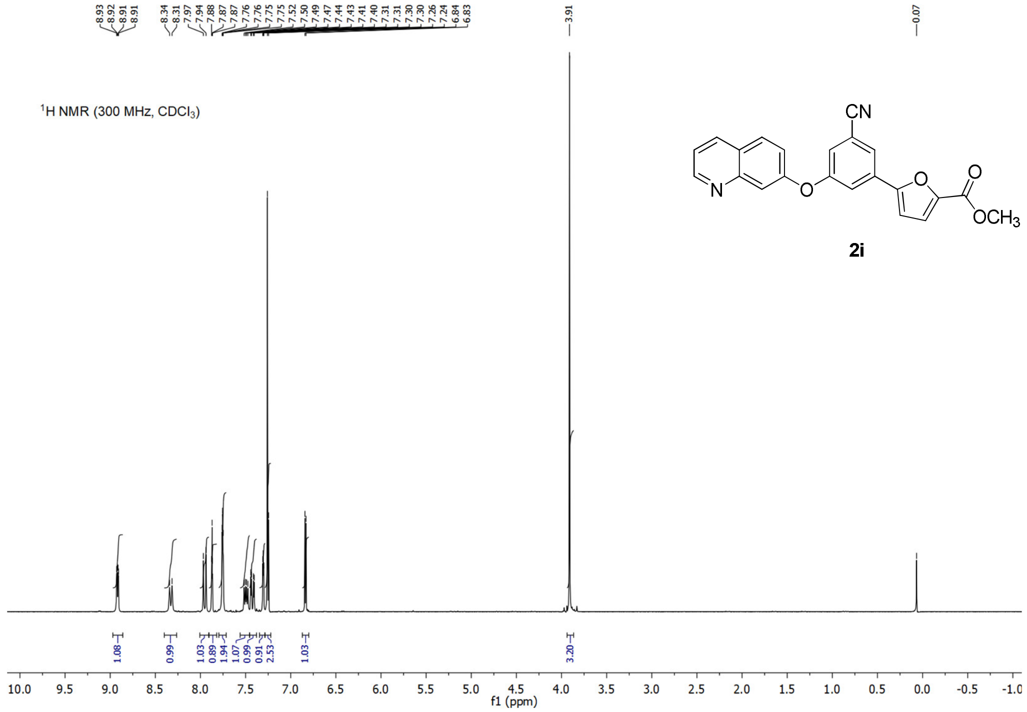

Figure S63. ^{1}H NMR spectrum of **2i**.

Methyl 5-(3-cyano-5-(naphthalen-2-ylmethoxy)phenyl)furan-2-carboxylate (2j)

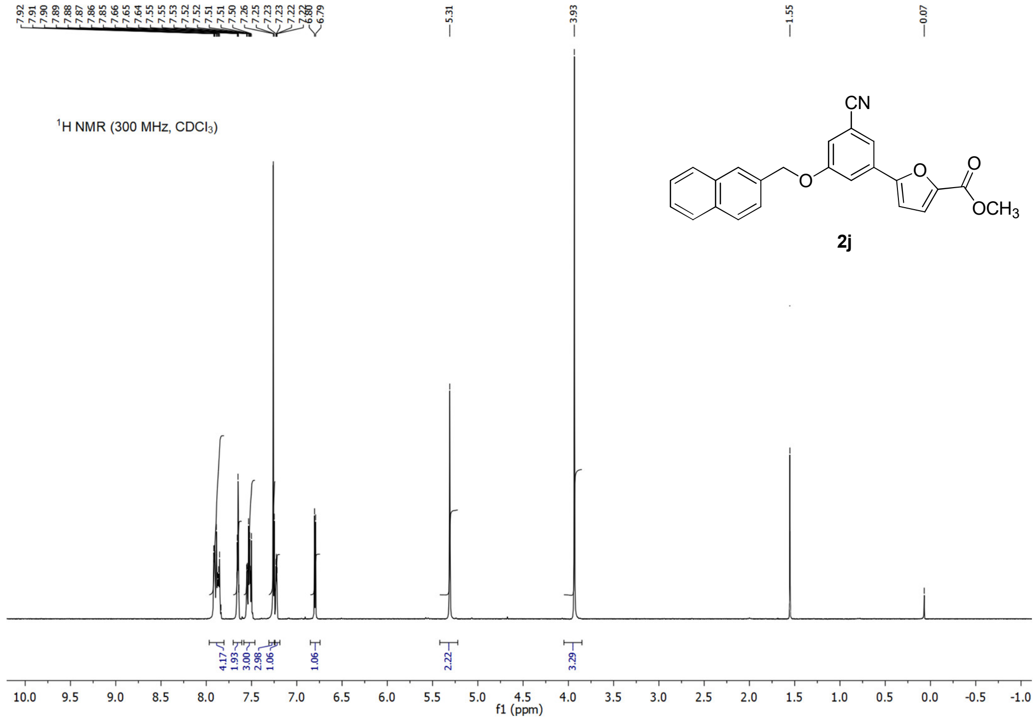

Figure S64. ^{1}H NMR spectrum of **2j**.

Methyl 5-(3-cyano-5-hydroxyphenyl)furan-2-carboxylate (3)

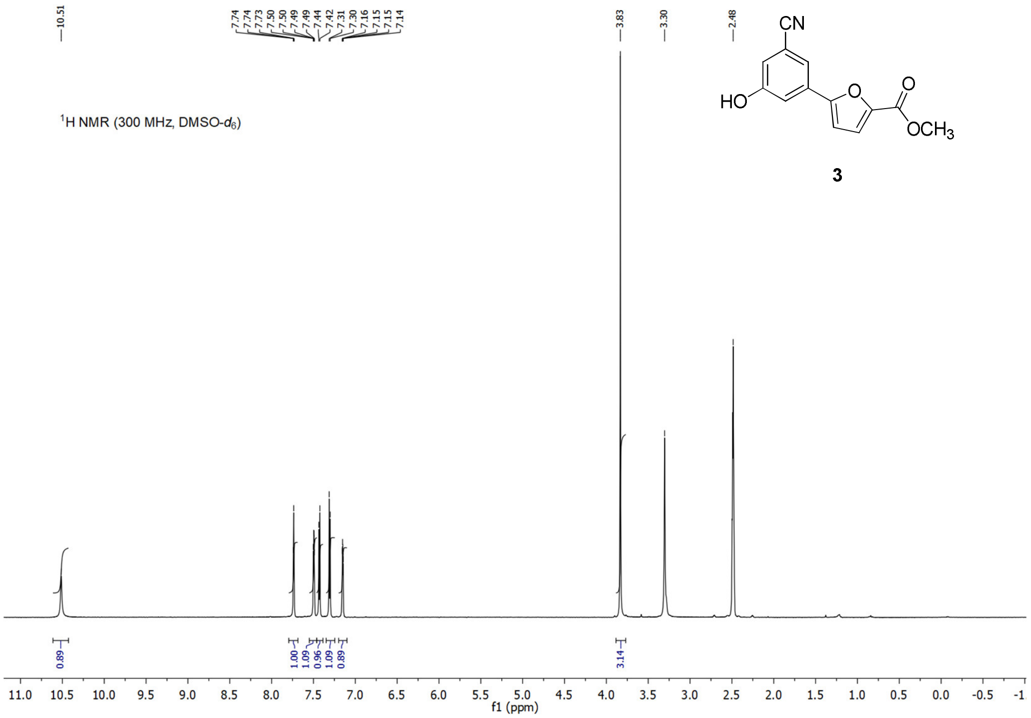

Figure S65. ^{1}H NMR spectrum of **3**.

3-Bromo-5-phenoxybenzonitrile (4a)

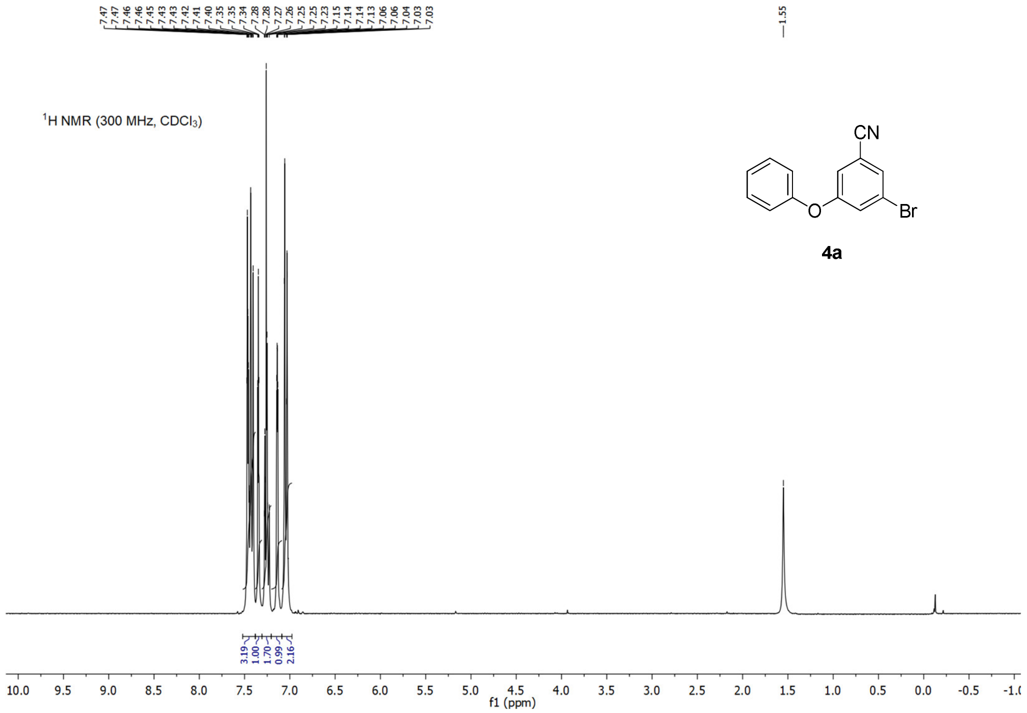

Figure S66. ^{1}H NMR spectrum of **4a**.

(*E*)-(3-Bromoprop-1-en-1-yl)benzene (4e)

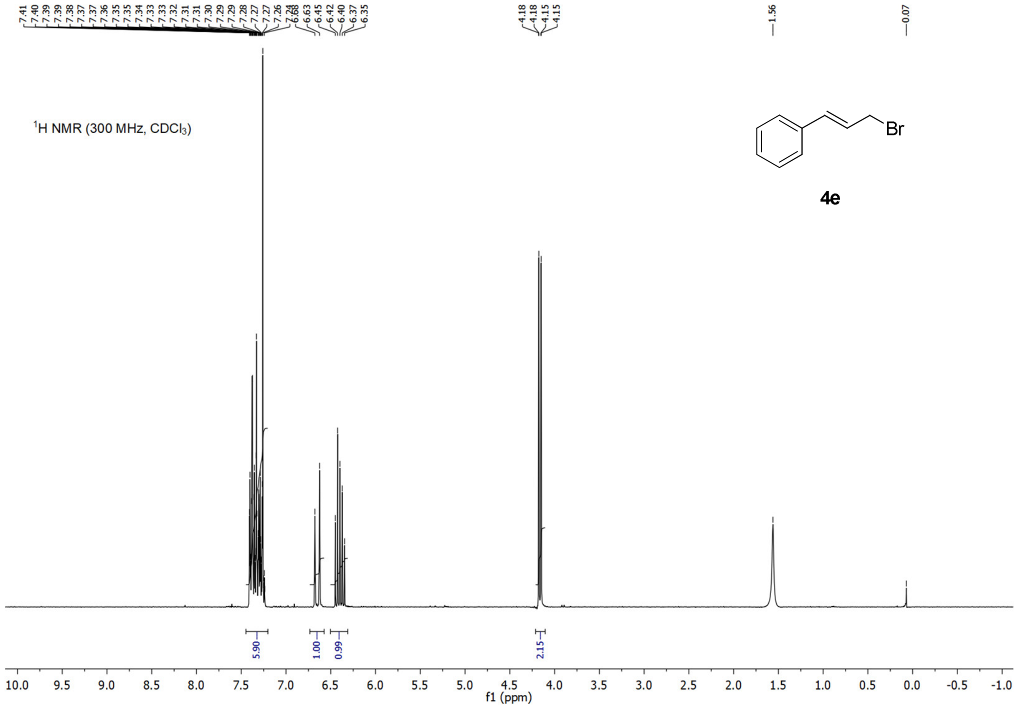

Figure S67. ^{1}H NMR spectrum of **4e**.

3-Bromo-5-(2-phenoxyethoxy)benzonitrile (4f)

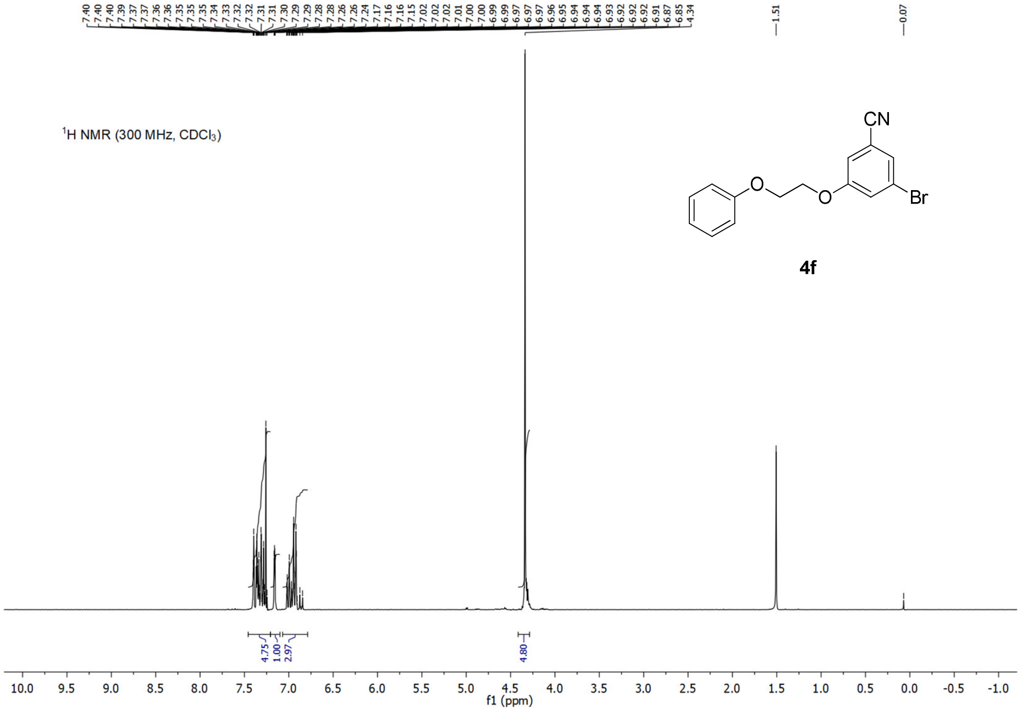

Figure S68. ^{1}H NMR spectrum of **4f**.

3-Bromo-5-(2-(phenylamino)ethoxy)benzonitrile (4g)

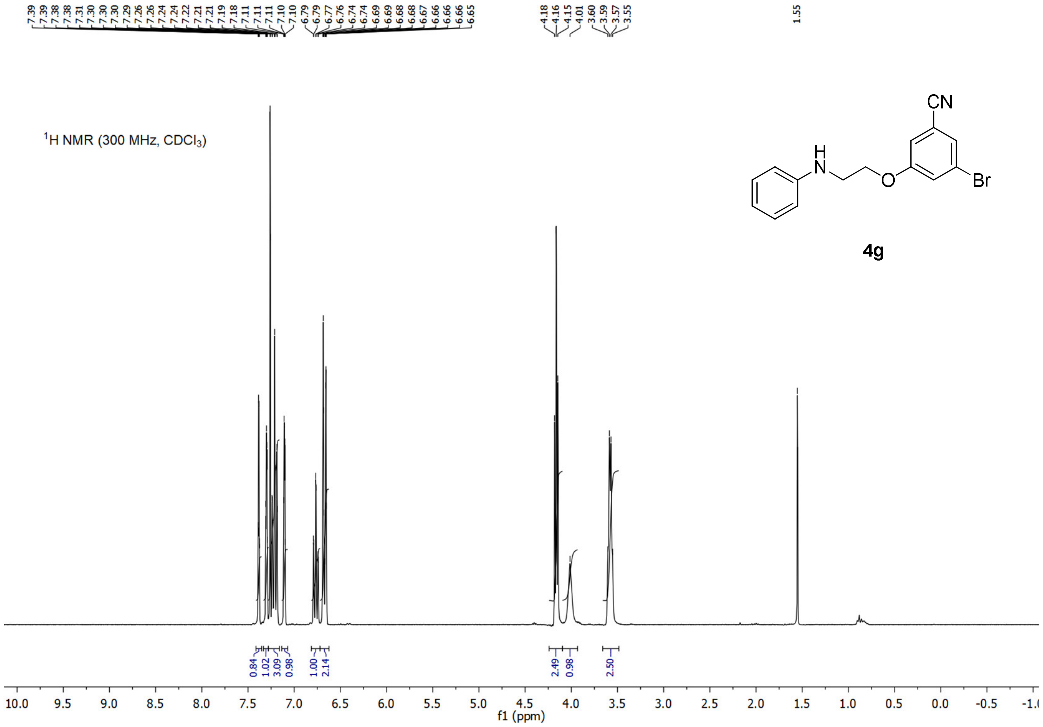

Figure S69. ^{1}H NMR spectrum of **4g**.

3-Bromo-5-(naphthalen-2-yloxy)benzonitrile (4h)

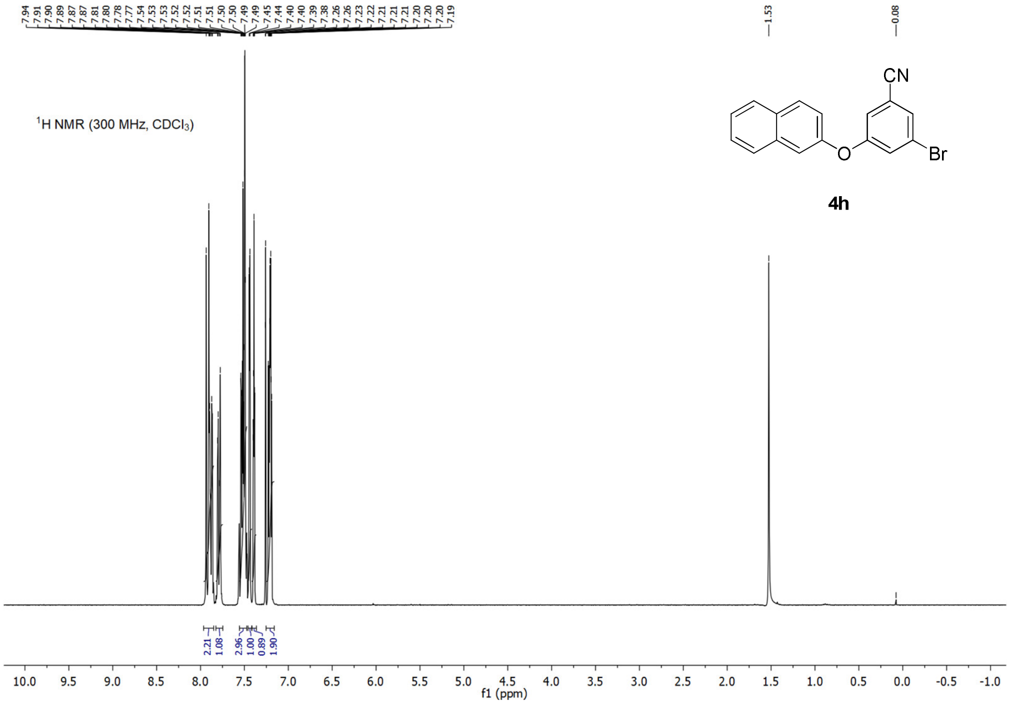

Figure S70. ^{1}H NMR spectrum of **4h**.

3-Bromo-5-(quinolin-7-yloxy)benzonitrile (4i)

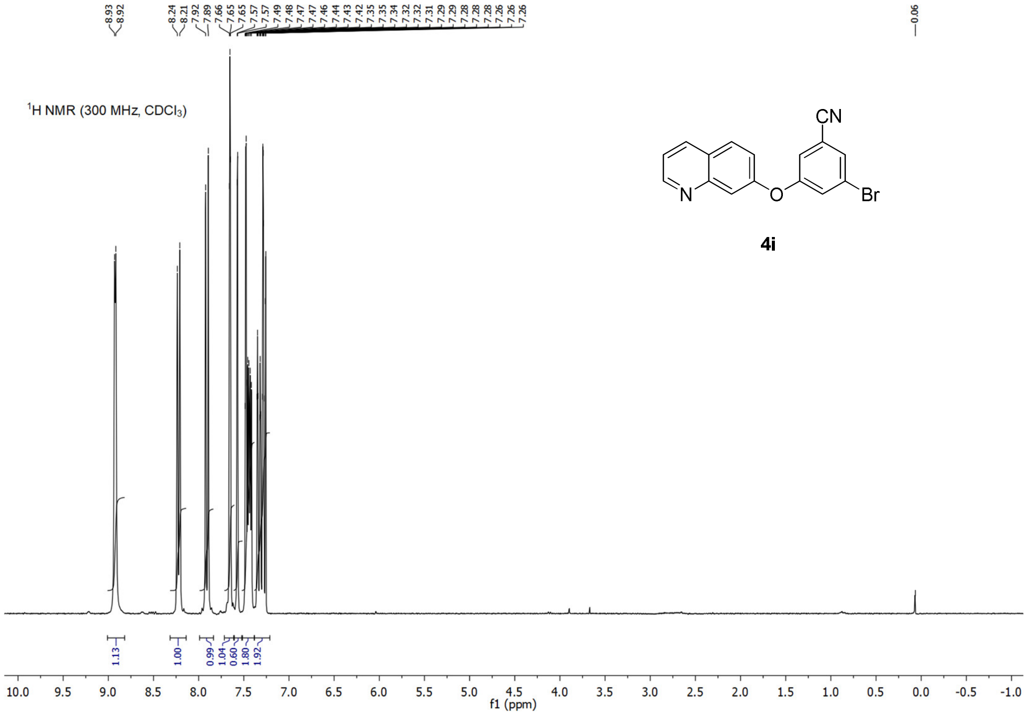

Figure S71. ^{1}H NMR spectrum of **4i**.

2-(Bromomethyl)naphthalene (4j)

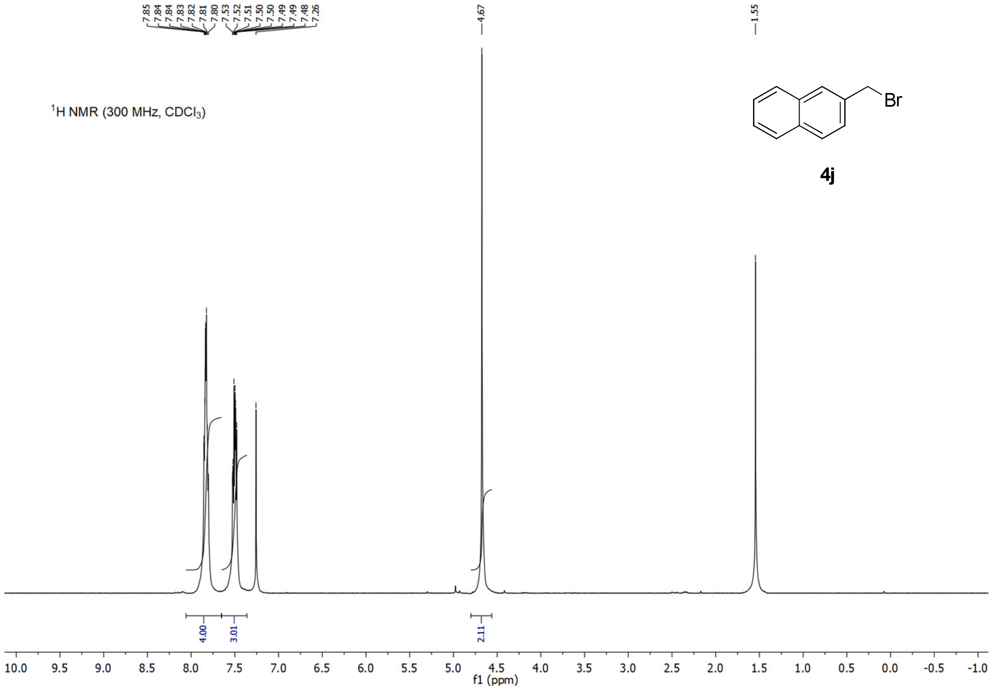

Figure S72. ^{1}H NMR spectrum of **4j**.

3-Bromo-5-hydroxybenzonitrile (5)

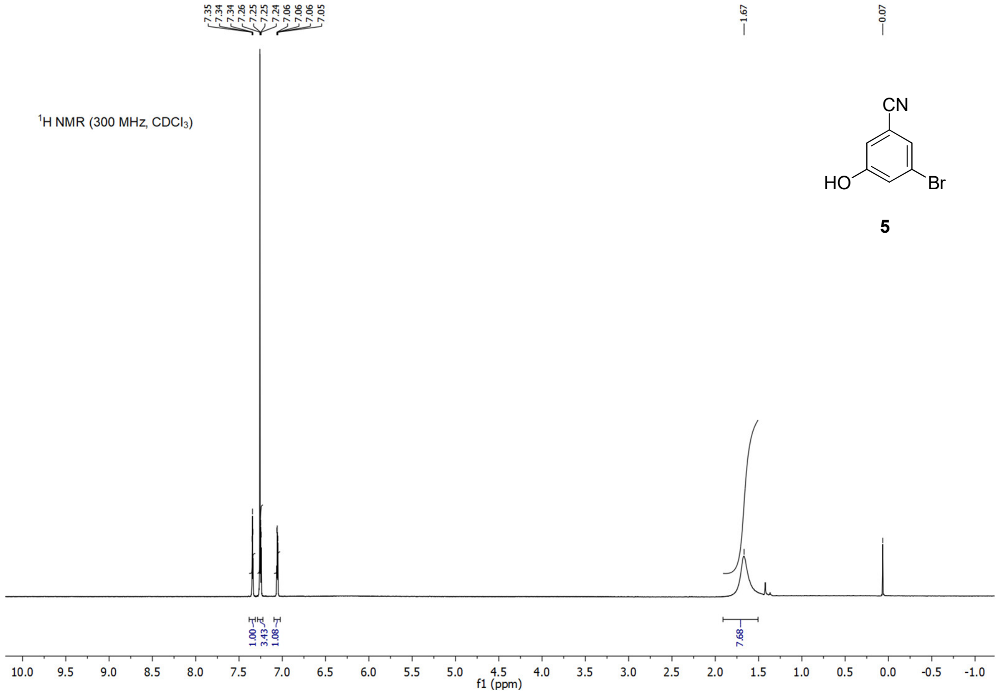

Figure S73. ^{1}H NMR spectrum of **5**.

2-(Phenylamino)-ethanol (6)

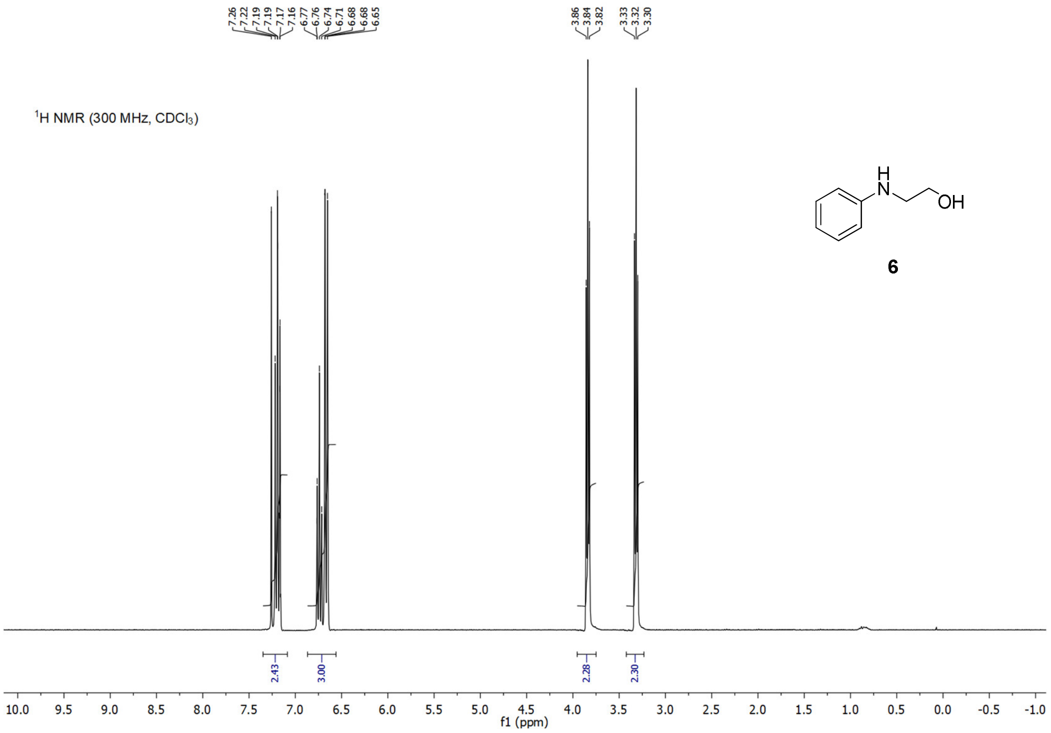

Figure S74. ^{1}H NMR spectrum of **6**.

3. Calculated LogP (cLogP) values

Table S1. Calculated LogP (cLogP) values for compounds **I** and **1a-j**. Data were obtained through the *DataWarrior* software (Sander, T. *et al. J. Chem. Inf. Model.* 2015, 55(2), 460–473).

Entry	cLogP
**I**	1.9192
**1a**	3.3148
**1b**	3.2673
**1c**	3.6974
**1d**	4.1518
**1e**	4.0285
**1f**	3.2742
**1g**	2.9912
**1h**	4.5092
**1i**	3.6308
**1j**	4.4617

4. Siderophore production assay

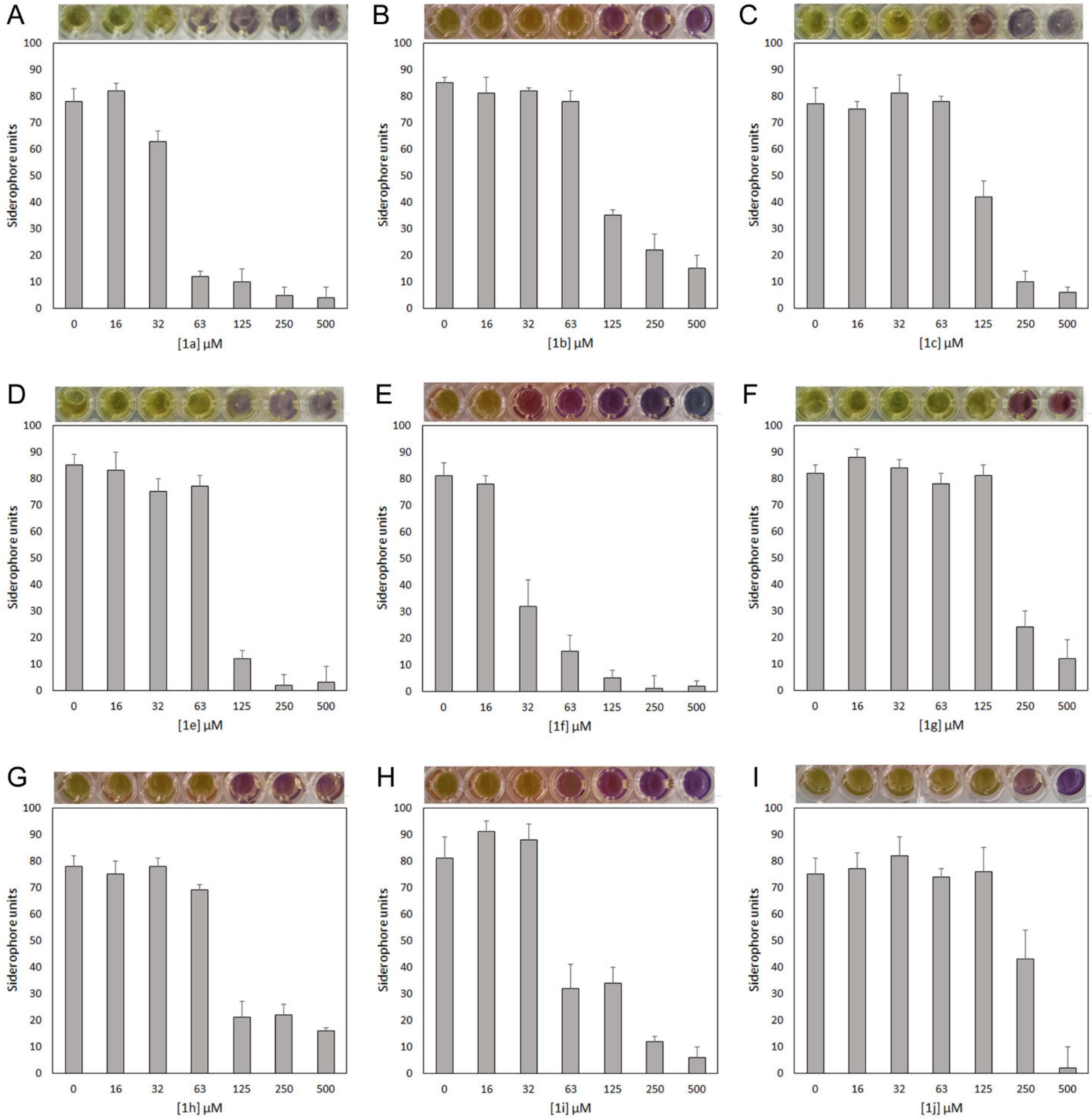

Figure S75. Universal CAS assay performed on *M. bovis* BCG cells grown in chelated Sauton's medium, in the presence of different concentrations of the compounds (**1a**-**j**). If siderophores are released, they remove iron from the CAS dye complex, leading to a color change from blue to orange. The absorbance at 630 nm of the iron-CAS complex is read, and the siderophore activity is determined using the equation reported in Materials and Methods. Values are mean and standard deviation of two independent experiments.

5. Infection assays

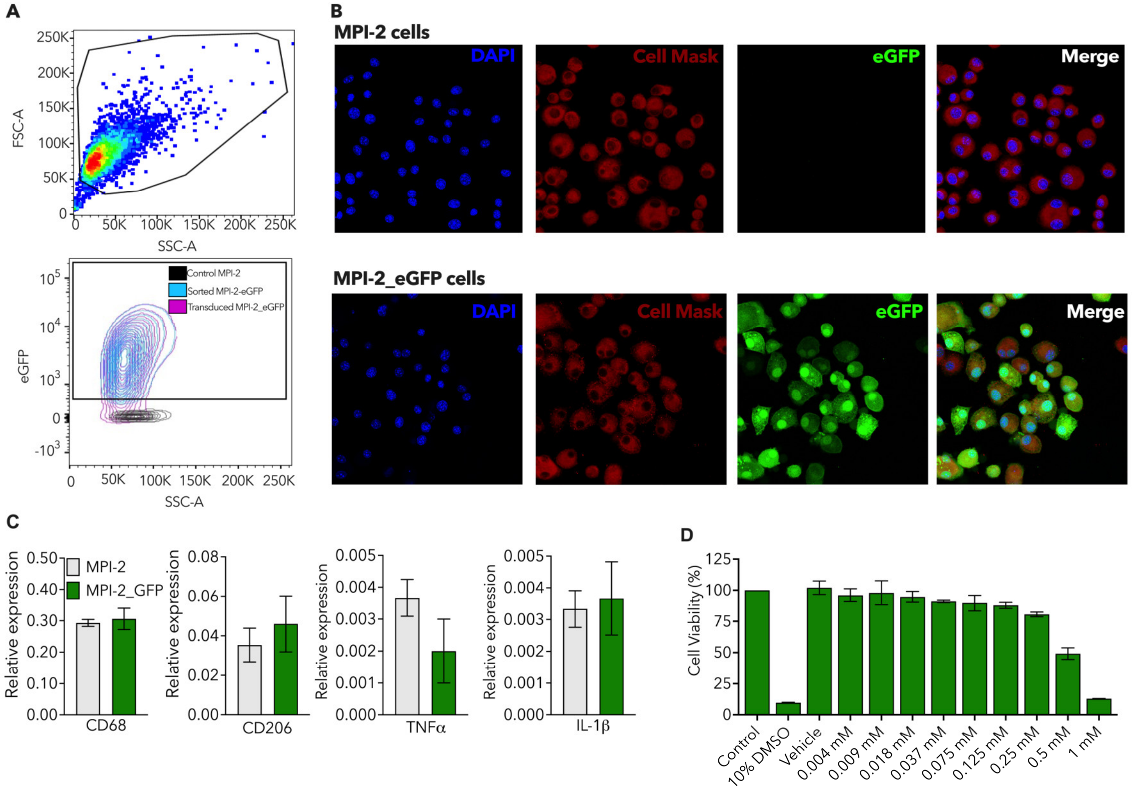

Figure S76. Generation and characterization of eGFP mAMs cells. **(A) (B)** Representative snapshot images of both MPI-2 wild type cells and MPI-2_eGFP. Nuclear (DAPI), cellular membrane (Cell Mask), eGFP (eGFP) are reported both as single channel and as a merge. Scale bar 50 µm. **(C)** qRT-PCR analysis of mAM immunogenic relating gene in both MPI-2 wild type (gray) and MPI-2_eGFP (green). Transcripts are normalized to total RNA and *gapdh* relative expression. Black lines indicate mean ±SD. Unpaired *t*-test with Welch's correction was performed to analyze statistical significance between multiple groups. Data are from three independent experiments. **(D)** MPI-2_EGFP viability following 24 h-treatment with increasing concentration of **1f**. Black lines indicate mean ±SD. Data are from three independent experiments.

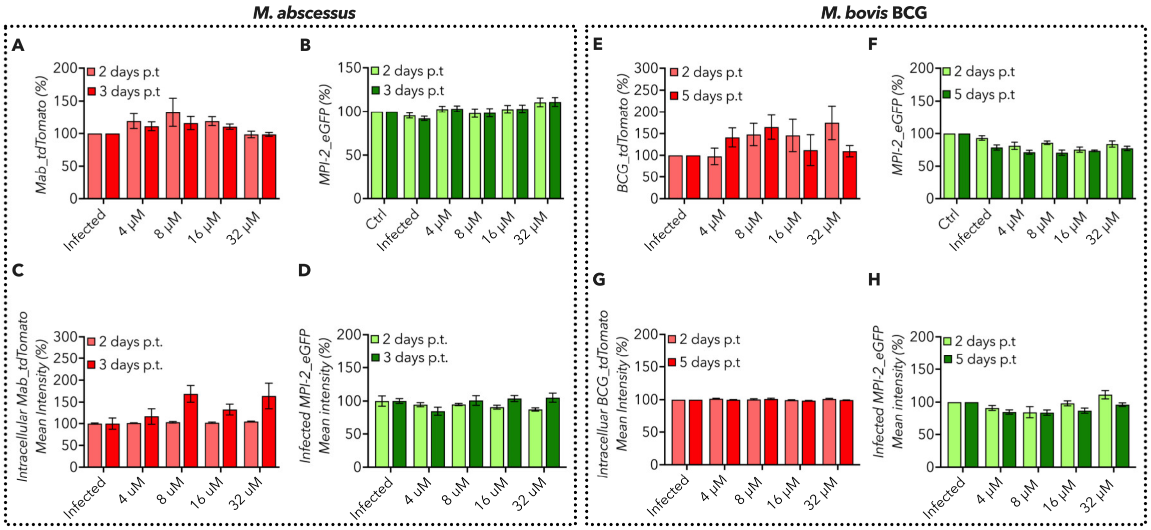

Figure S77. Evaluation of the antimycobacterial activity of **1f** in mAM cells. **(A, E)** *Mab_tdTomato* (**A**) and *M. bovis BCG_tdTomato* (**E**) fluorescence expressed in percentage with or without the treatment with increasing concentrations of **1f**. Black lines indicate mean ±SEM. Data are from five independent experiments. **(B, F)** *Mab_tdTomato* (**B**) and *M. bovis BCG_tdTomato* (**F**) infected MPI-2_eGFP fluorescence expressed in percentage with or without the treatment with increasing concentrations of **1f**. Black lines indicate mean ±SEM. Data are from five independent experiments. **(C, G)** Intracellular *Mab_tdTomato* (**C**) and *M. bovis BCG_tdTomato* (**G**) fluorescence expressed in percentage with or without the treatment with increasing concentrations of **1f**. Black lines indicate mean ±SEM. Data are from five independent experiments. **(D, H)** Only *Mab_tdTomato* (**B**) and *M. bovis BCG_tdTomato* (**F**) infected MPI-2_eGFP fluorescence expressed in percentage with or without the treatment with increasing concentrations of **1f**. Black lines indicate mean ±SEM. Data are from five independent experiments.
